# Recessive *TMEM167A* variants cause neonatal diabetes, microcephaly, and epilepsy syndrome

**DOI:** 10.1172/JCI195756

**Published:** 2025-09-09

**Authors:** Enrico Virgilio, Sylvia Tielens, Georgia Bonfield, Fang-Shin Nian, Toshiaki Sawatani, Chiara Vinci, Molly Govier, Hossam Montaser, Romane Lartigue, Anoop Arunagiri, Alexandrine Liboz, Flavia Natividade Da Silva, Maria Lytrivi, Theodora Papadopoulou, Matthew N. Wakeling, James Russ-Silsby, Pamela Bowman, Matthew B. Johnson, Thomas W. Laver, Anthony Piron, Xiaoyan Yi, Federica Fantuzzi, Sirine Hendrickx, Mariana Igoillo-Esteve, Bruno J. Santacreu, Jananie Suntharesan, Radha Ghildiyal, Darshan Hegde, Nikhil Shah, Sezer Acar, Beyhan Özkaya Dönmez, Behzat Özkan, Fauzia Mohsin, Iman M. Talaat, Mohamed Tarek Abbas, Omar Tarek Abbas, Hamed Ali Alghamdi, Nurgun Kandemir, Sarah E. Flanagan, Raphael Scharfmann, Peter Arvan, Matthieu Raoux, Laurent Nguyen, Andrew T. Hattersley, Miriam Cnop, Elisa De Franco

**Affiliations:** 1 ULB Center for Diabetes Research, Université Libre de Bruxelles, Brussels, Belgium.; 2 Laboratory of Molecular Regulation of Neurogenesis, GIGA Institute, University of Liège, Liège, Belgium.; 3 Department of Clinical and Biomedical Sciences, Faculty of Health and Life Sciences, University of Exeter, Exeter, United Kingdom.; 4 Stem Cells and Metabolism Research Program, Faculty of Medicine, University of Helsinki, Helsinki, Finland.; 5 University of Bordeaux, CNRS, Bordeaux INP, Chemistry & Biology of Membranes & Nano-objects, UMR 5248, Pessac, France.; 6 Division of Metabolism, Endocrinology & Diabetes, University of Michigan Medical Center, Ann Arbor, Michigan, USA.; 7 Division of Endocrinology, ULB Erasmus Hospital, Brussels University Hospital, Université Libre de Bruxelles, Brussels, Belgium.; 8 Sirimavo Bandaranayake Specialized Children’s Hospital, Peradeniya, Sri Lanka.; 9 Department of Pediatrics, Lokmanya Tilak Municipal Medical College and Lokmanya Tilak Municipal General Hospital, Mumbai, India.; 10 Division of Pediatric Endocrinology, Dr. Behçet Uz Children’s Education and Research Hospital, Izmir, Turkey.; 11 Department of Paediatrics, BIRDEM General Hospital, Dhaka, Bangladesh.; 12 Pediatric Department and; 13 Internal medicine, Faculty of Medicine, Ain Shams University, Cairo, Egypt.; 14 Northern Area Armed Forces Hospital, King Khalid Military City, Saudi Arabia.; 15 Hacettepe University, Faculty of Medicine, Department of Pediatric Endocrinology, Ankara, Turkey.; 16 Université Paris Cité, Institut Cochin, INSERM, Paris, France.; 17 WEL Research Institute, Wavre, Belgium.

**Keywords:** Cell biology, Endocrinology, Genetics, Beta cells, Genetic diseases, Neurodevelopment

## Abstract

Understanding the genetic causes of diseases that affect pancreatic β cells and neurons can give insights into pathways essential for both cell types. Microcephaly, epilepsy, and diabetes syndrome (MEDS) is a congenital disorder with two known etiological genes, *IER3IP1* and *YIPF5*. Both genes encode proteins involved in endoplasmic reticulum (ER) to Golgi trafficking. We used genome sequencing to identify 6 individuals with MEDS caused by biallelic variants in the potentially novel disease gene *TMEM167A*. All had neonatal diabetes (diagnosed at <6 months) and severe microcephaly, and 5 also had epilepsy. *TMEM167A* is highly expressed in developing and adult human pancreas and brain. To gain insights into the mechanisms leading to diabetes, we silenced *TMEM167A* in EndoC-βH1 cells and knocked-in one patient’s variant, p.Val59Glu, in induced pluripotent stem cells (iPSCs). Both *TMEM167A* depletion in EndoC-βH1 cells and the p.Val59Glu variant in iPSC-derived β cells sensitized β cells to ER stress. The p.Val59Glu variant impaired proinsulin trafficking to the Golgi and induced iPSC-β cell dysfunction. The discovery of *TMEM167A* variants as a genetic cause of MEDS highlights a critical role of TMEM167A in the ER to Golgi pathway in β cells and neurons.

## Abstract 2

We discovered TMEM167A variants as a cause of neonatal diabetes and microcephaly. Functional experiments show that TMEM167A is involved in protein trafficking in pancreatic beta-cells.

## Introduction

Understanding the genetic causes of rare diseases that affect the pancreatic β cells and neurons can give important insights into the pathways essential for development and function of both cell types. Pathogenic variants in over 20 genes have been reported to cause monogenic forms of diabetes with neurological features, ranging from developmental delay to structural abnormalities such as microcephaly. These include genes essential for the development of both pancreatic and brain structures (e.g., *PTF1A* [ref. 1]), genes with essential mitochondrial functions (e.g., *TRMT10A* [ref. 2], *TARS2* [ref. 3]), and, most commonly, genes involved in function and maintenance of protein homeostasis within the endoplasmic reticulum (ER) (e.g., *WFS1* [ref. 4], *EIF2AK3* [ref. 5]).

Microcephaly, epilepsy, and diabetes syndrome (MEDS) is a rare disorder with onset in the neonatal period. Two genetic etiologies of MEDS have been described to date, both autosomal recessive. MEDS1 (MIM 614231) is caused by recessive variants in the *IER3IP1* gene (6). This condition has been reported in 11 individuals so far (6–14), and, in addition to the classical MEDS features, a simplified gyral pattern has been described in some individuals. More recently, partial loss-of-function variants in *YIPF5* were identified in 6 individuals with MEDS2 (MIM 619278) (15). In vitro studies of *YIPF5* (15) and *IER3IP1* (16) variants in stem cell–derived β cells showed that they result in increased β cell susceptibility to ER stress–induced cell death due to impaired ER to Golgi trafficking of proinsulin. These studies showed that YIPF5 and IER3IP1 both play key roles in ER to Golgi transport and highlighted the critical role of this pathway for human β cell function and survival.

In this study, we report the identification of biallelic variants in the *TMEM167A* gene as a third genetic cause of MEDS. Functional characterization of this disease gene highlights its role in ER to Golgi transport in human β cells.

## Results

### Identification of TMEM167A variants in 6 individuals with MEDS.

**Figure 1:**
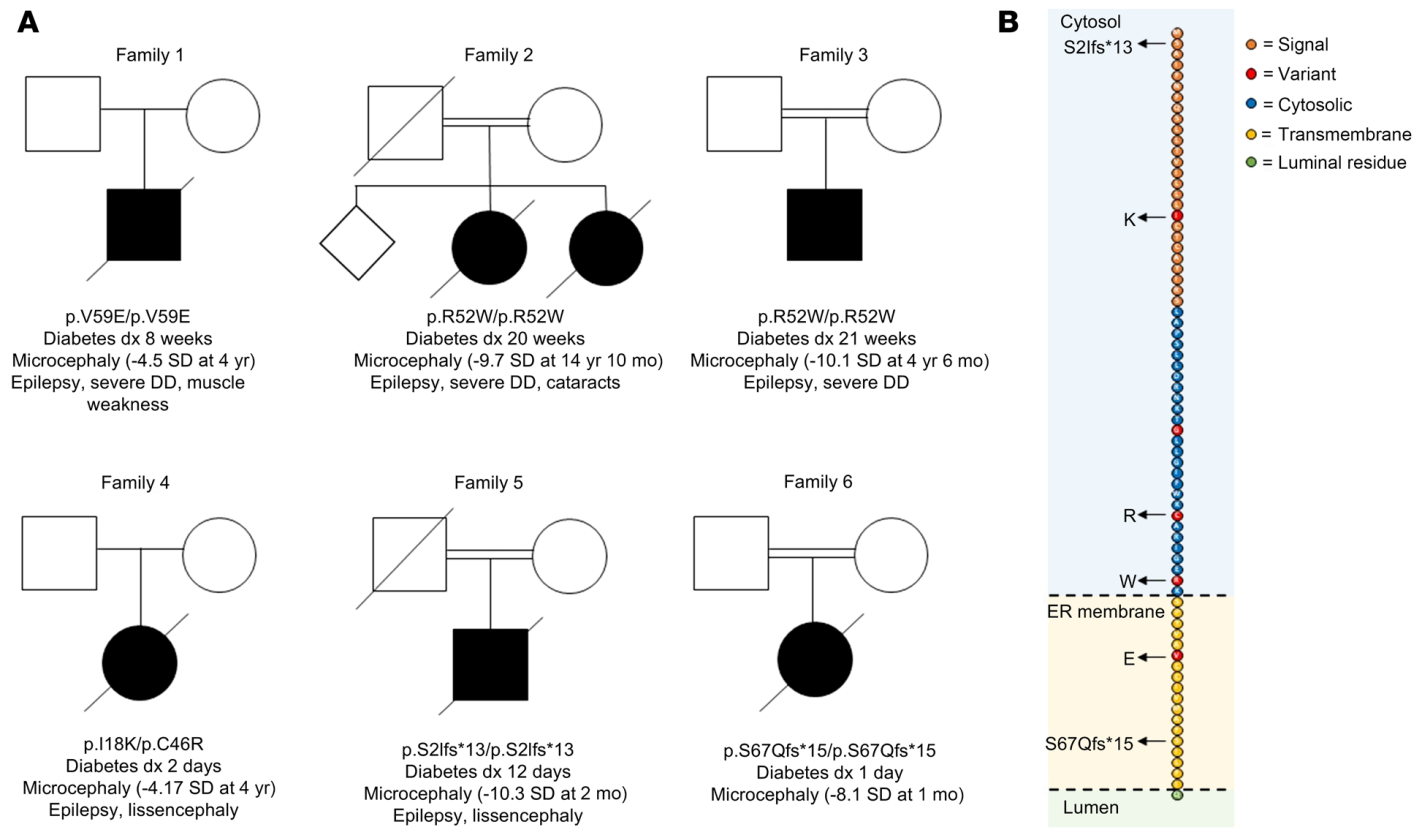
Identification of recessive *TMEM167A* variants in individuals with MEDS. (**A**) Partial pedigrees and summary of clinical features of the 6 patients with recessive *TMEM167A* variants. Head circumference standard deviation (SD) below the mean is given in parentheses. DD, developmental delay. (**B**) Schematic representation of the TMEM167A transmembrane protein with variant positions indicated by black arrows. Domains are represented as predicted in UniProtKB (https://www.uniprot.org/).

We performed genome sequencing to identify the genetic cause of disease in 2 individuals with neonatal diabetes (diagnosed before the age of 6 months), microcephaly, and epilepsy. All known genetic causes of neonatal diabetes, including recessive variants in the 2 MEDS genes *IER3IP1* and *YIPF5*, had been previously excluded. Homozygosity analysis from genome data revealed a homozygosity of 1.23% and 8.04% in individual 1 (I-1) and I-2, respectively. Analysis of autosomal recessive, rare, shared coding variants between the 2 individuals identified missense variants in the *TMEM167A* gene in both probands: p.Val59Glu in I-1 and p.Arg52Trp in I-2 (Figure 1, A and B, and Supplemental Table 1; supplemental material available online with this article; https://doi.org/10.1172/JCI195756DS1). The p.Val59Glu was absent in the Genome Aggregation Database (gnomAD) v4 (17), while the p.Arg52Trp was found to be rare (10 heterozygotes, no homozygotes, minor allele frequency = 0.000006205). No other rare, coding, homozygous variants affecting the same gene in both individuals were identified (Supplemental Table 1).

Replication studies in 284 additional individuals diagnosed with diabetes before 6 months of age, 7 of whom had microcephaly, identified biallelic variants in 4 additional individuals (Figure 1, A and B). I-3 was homozygous for the same p.Arg52Trp variant identified in I-2. I-4 was compound heterozygous for 2 missense variants, p.Ile18Lys and p.Cys46Arg. I-5 was homozygous for a c.113G>A variant affecting the last nucleotide of exon 2. An exon trapping assay confirmed that this variant resulted in skipping of exon 2 and is predicted to result in a frameshift, p.Ser2Ilefs*13 (Supplemental Figure 1). This frameshift is not predicted to undergo nonsense-mediated decay, but it completely disrupts the amino acid sequence from residue 2, consistent with a complete loss of TMEM167A protein function. I-6 was homozygous for a frameshift variant in the last exon (p.Ser67Glnfs*15), which affected 5 residues within the TMEM167A transmembrane domain and resulted in translation of an elongated protein. While this variant is also predicted to escape nonsense-mediated decay, its effect on transmembrane residues is consistent with the resulting protein not localizing to the transmembrane (Supplemental Figure 2), again consistent with complete loss of function.

All variants were rare or absent in gnomAD v4. Using the available evidence, all identified variants were classified as likely pathogenic according to the American College of Medical Genetics and Genomics (ACMG) variant interpretation guidelines (18–20) (Supplemental Table 2).

### Clinical features of individuals with recessive TMEM167A variants.

The clinical features of the 6 individuals with biallelic *TMEM167A* variants are summarized in Table 1. All 6 individuals were diagnosed with diabetes in the neonatal period (median age at diagnosis 34 days, range 1 day to 22 weeks) and were treated with insulin from diagnosis (median insulin dose 1.13 U/kg/d, range 0.6–1.7 U/kg/d). Plasma C-peptide measurement was not available for any of the affected individuals. Birth weight was variable with 3 of 6 individuals having intrauterine growth retardation (median *z* score –1.45, range –2.87 to 1.99).

All individuals had severe microcephaly (median head circumference –8.9 SD, range –4.17 to –10.3), and 5 of 6 had been diagnosed with epilepsy in the neonatal period. I-6 was the only individual not confirmed to have epilepsy before she died aged 59 days. In I-4 and I-5, brain imaging was consistent with a diagnosis of lissencephaly. Five patients were deceased (age of death range 2 months to 14 years). I-2 had a sister who was similarly affected and died in the neonatal period. Her DNA was not available for testing.

A family history of diabetes was reported for I-2, with both parents diagnosed with diabetes (mother at 48 years and father at 40).

### TMEM167A expression in the developing mouse and human brain.

To gain insights into the mechanism linking loss-of-function variants in *TMEM167A* to MEDS, we analyzed *Tmem167*/*TMEM167A* mRNA levels in mouse and human brain (Supplemental Figure 3A). In mouse, *Tmem167* was stably expressed in the pallium from embryonic day (E) 12.5 to adulthood, while its expression in the subpallium increased in adult brain (Supplemental Figure 3, B and C). At E12.5, E14.5, and E16.5 *Tmem167* mRNA was present in Sox2-positive progenitors, Tbr2-positive intermediate progenitors, and Tbr1-positive neurons (Supplemental Figure 3D).

**Figure 2:**
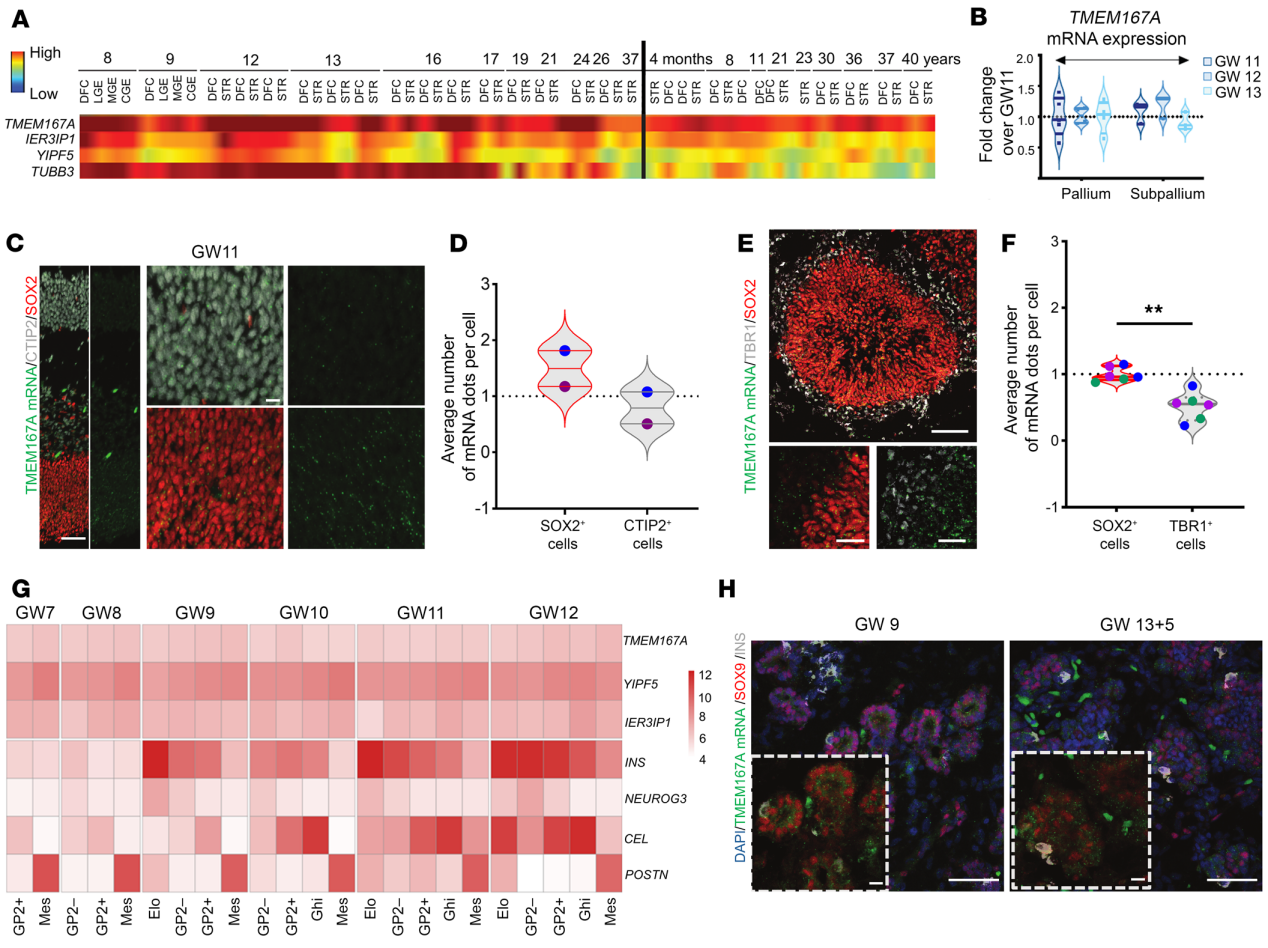
Expression of *TMEM167A* in human brain and pancreas. (**A**) TMEM167A, IER3IP1, and YIPF5 expression from the BrainSpan Atlas (https://www.brainspan.org/) across development in dorsofrontal cortex (DFC); lateral (LGE), medial (MGE), and caudal (CGE) ganglionic eminences; and striatum (STR). (**B**) *TMEM167A* mRNA expression in human pallium and subpallium at gestational weeks (GW) 11–13. Expression at GW11 was set to 1.0; other stages are relative. Violin plots show median ± quartiles of 4 pallium and 3 subpallium samples. (**C**) RNAscope and immunolabeling of GW11 cortex showing *TMEM167A* mRNA (green), CTIP2 (gray), and SOX2 (red). The left panel shows merged staining (left) and TMEM167A alone (right). Magnified views of CTIP2-positive (top) and SOX2-positive (bottom) regions are shown in the right panels, with adjacent images displaying TMEM167A only. Scale bars: 50 μm, 10 μm (close-up). (**D**) Quantification of *TMEM167A* mRNA dots per cell in SOX2- and CTIP2-positive cells within a square with 100 μmlong sides. Violin plots show median ± quartiles from two GW11 samples. (**E**) Immunolabeling of rosettes in cerebral organoids showing *TMEM167A* mRNA (green), TBR1 (gray), and SOX2 (red). Scale bars: 50 μm, 20 μm (close-up). (**F**) Quantification of *TMEM167A* mRNA dots per cell in SOX2- and TBR1-positive rosette cells from 3 organoids. Violin plots show median ± quartiles from 3 biological replicates, with each rosette per organoid represented by a uniquely colored dot. **P < 0.01. (**G**) Heatmap of *TMEM167A*, *YIPF5*, and *IER3IP1* expression in human embryonic pancreas (GW7–12) based on microarray data from FACS-sorted epithelial and mesenchymal populations. GP2+ indicates pancreatic progenitors; Mes, mesenchymal cells; GP2−, ductal/endocrine progenitors; Elo, endocrine cells; and Ghi, acinar cells. INS, NEUROG3, CEL, and POSTN serve as markers of β, pancreatic progenitor, acinar, and mesenchymal cells, respectively. (**H**) RNAscope and immunolabeling of GW9 and GW13 + 5 days human pancreas showing expression of *TMEM167A* mRNA (green), insulin (gray), and SOX9 (red) and nuclear counterstaining (DAPI, blue). Scale bars: 50 μm, 10 μm (close-up).

In the developing human brain, *TMEM167A* is highly expressed in the pallial dorsofrontal cortex, subpallial ganglionic eminences, and striatal area at every stage, with the highest expression noted during critical phases of embryonic brain development (Figure 2A), according to the BrainSpan database (https://www.brainspan.org/). *YIPF5* and *IER3IP1* were also detected in all aforementioned regions, albeit with lower expression. To validate this, we assessed *TMEM167A* mRNA expression by quantitative PCR (qPCR) in human embryonic brain at gestational week (GW) 11 to GW13, showing that *TMEM167A* was consistently expressed in pallium and subpallium regions (Figure 2B). To probe *TMEM167A* expression in different brain cell types, we labeled its mRNA with RNAscope on GW11 brain slices, since no TMEM167A antibody is available. The expression profiles of the positive and negative control probes in mouse and human fetal brain tissues are provided (Supplemental Figure 4). *TMEM167A* mRNA dots were more abundant in SOX2-positive progenitors than in CTIP2-positive neurons at this stage (Figure 2, C and D, and Supplemental Figure 5).

As an alternative model to study early brain development, we established the differentiation of human embryonic stem cells into cerebral organoids. In the rosette of the cerebral organoid at day 42 of culture, *TMEM167A* mRNA dots appeared more abundant in SOX2-positive progenitors than in TBR1-positive neurons (Figure 2, E and F), suggesting that *TMEM167A* is more expressed in neural progenitors than in neurons, consistent with the findings in human brain sections (Figure 2, C and D).

### TMEM167A expression in the developing mouse and human pancreas.

In mouse pancreas sections at stages E14.5, E16.5, and adulthood, *Tmem167* mRNA staining was present in both insulin-positive β cells and Sox9-positive ductal cells (Supplemental Figure 6A). Mouse pancreas *Tmem167* mRNA levels, assessed by qPCR, were stable from E12.5 to adulthood (Supplemental Figure 6B). *Tmem167* mRNA is ubiquitously and uniformly expressed in neonatal, adult, and old mouse α, β, and δ cells (21) (Supplemental Figure 6C).

In adult human tissues, *TMEM167A* is ubiquitously expressed, with particularly high expression in islets (TIGER data portal, ref. 22). To gain insights into *TMEM167A* expression in the developing human pancreas, we examined *TMEM167A* mRNA expression in distinct cell populations of human embryonic pancreas from microarray data of FACS-sorted epithelial and mesenchymal populations from GW7 to GW12 (23). *TMEM167A* was ubiquitously expressed in mesenchymal, acinar (glycoprotein 2–high), ductal/endocrine progenitor (glycoprotein 2–negative E-cadherin–positive), and endocrine (glycoprotein 2–negative E-cadherin–low) cells (Figure 2G). *YIPF5* and *IER3IP1* were also present in all pancreatic cell populations, with somewhat higher expression in mesenchymal and acinar cells. To validate the microarray data, we assessed *TMEM167A* mRNA expression by in situ hybridization of human pancreas sections at GW9 and GW13 + 5 days. *TMEM167A* was detected in both insulin-positive and SOX9-positive cells (Figure 2H), consistent with the mouse and human microarray data and pointing to ubiquitous *TMEM167A* expression during pancreatic development.

### TMEM167A silencing sensitizes clonal human β cells to ER stress.

To evaluate the impact of TMEM167A deficiency in human pancreatic β cells, we silenced *TMEM167A* by RNA interference in the human β cell line EndoC-βH1. *TMEM167A* mRNA expression was knocked down by 60%–70% using 2 different siRNAs (Supplemental Figure 7, A and B).

Treatment with the chemical ER stressor brefeldin A, which induces ER stress by blocking ER to Golgi transport (Supplemental Figure 7A), or thapsigargin, which induces ER stress by depleting ER Ca^2+^ (Supplemental Figure 7B), induced *TMEM167A* expression, albeit to a lower extent with thapsigargin than with brefeldin A. A mild increase in apoptosis was detected in *TMEM167A*-depleted cells upon treatment with these ER stressors, but not under basal conditions (Supplemental Figure 7, C and D). mRNA expression of the ER stress marker *CHOP* was increased upon exposure of *TMEM167A*-depleted β cells to brefeldin A; there was no change in expression of the ER chaperone BiP (Supplemental Figure 7, E–H).

*TMEM167A* silencing did not affect EndoC-βH1 cell function, as insulin content and insulin secretion induced by high glucose or high glucose plus forskolin were comparable in *TMEM167A*-depleted and *TMEM167A*-competent cells (Supplemental Figure 7, I–K).

### The TMEM167A variant p.Val59Glu does not prevent β cell development.

We sought to generate a more patient- and variant-relevant β cell model to assess the impact of the *TMEM167A* variants. Since it was not possible to obtain cells from any of the individuals with *TMEM167A* recessive variants, we used CRISPR/Cas9 gene editing to insert the homozygous c.176T>A, p.Val59Glu variant (present in I-1) in the induced pluripotent stem cell (iPSC) line 1.023, generating two iPSC clones, called V59E.37 and V59E.48 (Supplemental Figure 8, A and B). These iPSCs had a normal karyotype (Supplemental Figure 8C), no evidence of CRISPR/Cas9–induced off-target indels, and normal morphology. They expressed pluripotency markers and differentiated into the 3 germ layers in an embryoid body assay (Supplemental Figure 9), showing they fully retained their stemness.

We differentiated the wild-type and 2 mutant *TMEM167A* iPSC lines into β-like cells, a powerful model to study β cell development, function, and survival. We used a previously described 7-stage protocol (24–26) and a long-term protocol, extended by a 4-week culture of iPSC-derived β cell aggregates to enhance their functional maturation (27) (Supplemental Figure 10). *TMEM167A* mRNA was expressed at all stages of differentiation in wild-type and mutant cells, and key differentiation markers were similar, except for reduced *SOX9* expression in pancreatic progenitor cells (stage 4 [S4]) carrying the variant (Supplemental Figure 11). We also examined *TMEM167A* mRNA expression by bulk RNA sequencing along the differentiation from pancreatic progenitor S4 to 4-week extended culture (see below). *TMEM167A* was expressed at all stages of differentiation, without difference between wild-type and mutant cells (Supplemental Figure 12A). *TMEM167A* expression in iPSC-derived aggregates was similar to that of adult organ donor human islets from ref. 28 and FACS-sorted β and α cells from the Human Pancreas Analysis Program (29, 30) (Supplemental Figure 12B). *TMEM167A* was more abundant in β cells than whole islets (Supplemental Figure 12B).

**Figure 3:**
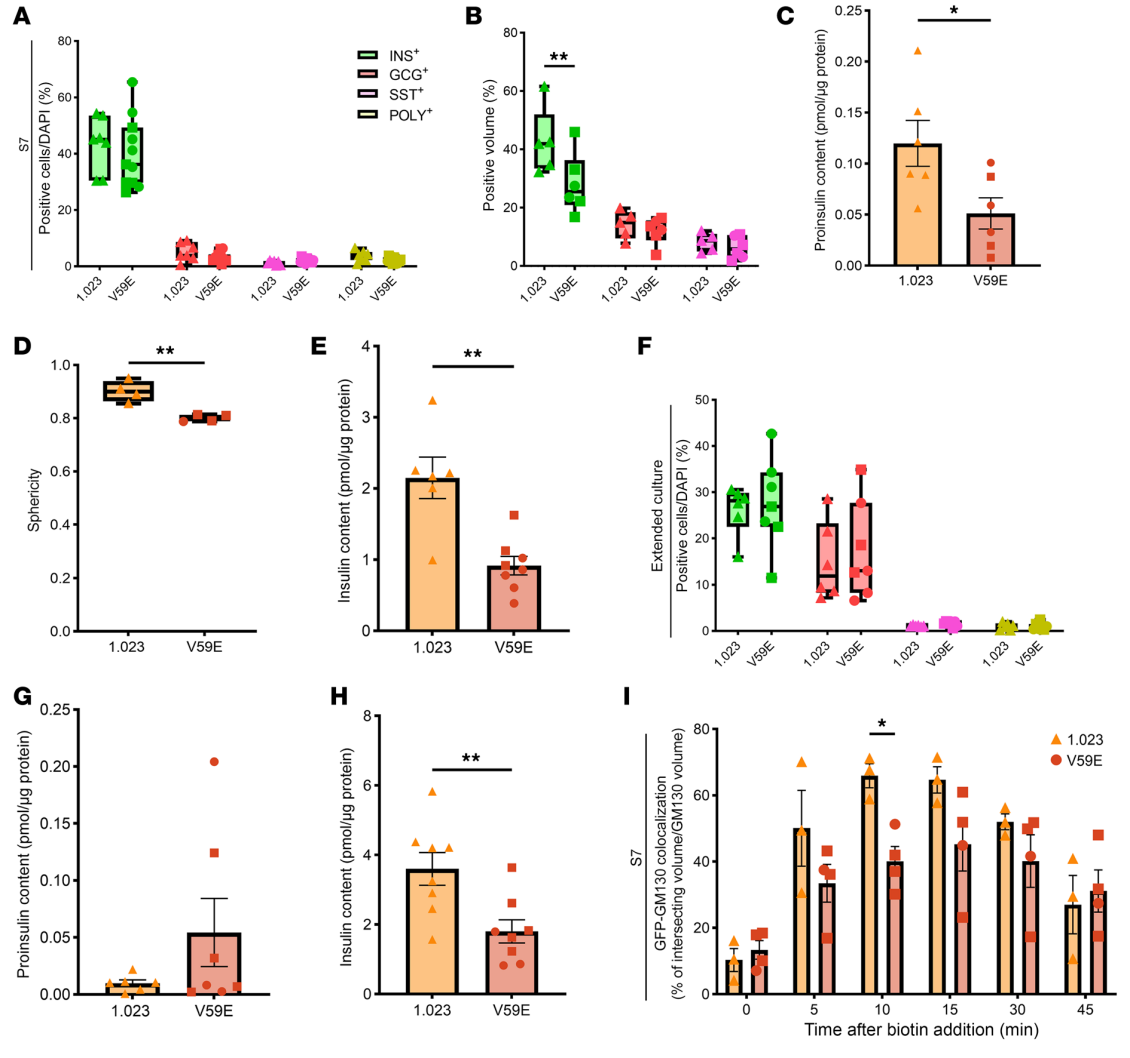
The *TMEM167A* V59E variant does not impact β cell differentiation but lowers insulin content. (**A**) Quantification of dispersed S7 iPSC-β cell aggregates stained for insulin (green), glucagon (red), and somatostatin (pink); nuclei were stained with DAPI (*n* = 7–11). Polyhormonal cells are shown in yellow. (**B** and **C**) Quantification of hormonal volume (**B**) and sphericity (**C**) by light sheet microscopy of S7 iPSC-β cell aggregates stained for insulin, glucagon, and somatostatin (*n* = 4). (**D** and **E**) Proinsulin (**D**) and insulin (**E**) content normalized to total protein content of S7 iPSC-β cell aggregates differentiated from control and mutant iPSCs (*n* = 6–8). (**F**) Quantification of dispersed long-term differentiated iPSC-β cell aggregates stained for insulin (green), glucagon (red), and somatostatin (pink); nuclei were stained with DAPI (*n* = 6–7). (**G** and **H**) Proinsulin (**G**) and insulin (**H**) content normalized to total protein content of long-term culture iPSC-β cell aggregates differentiated from control and mutant iPSCs (*n* = 8). (**I**) Colocalization at each time point of the RUSH assay by i1 intersection coefficient, i.e., percentage of intersecting volume/GM130 volume (*n* = 3–4) in dispersed S7 iPSC-β cells. Triangles represent mother cell line 1.023, squares cell line 1.023 mutTMEM167A V59E.37, and circles cell line 1.023 mutTMEM167A V59E.48. The median is shown by horizontal lines in the box plots; 25th and 75th percentiles are at the bottom and top of the boxes; whiskers represent minimum and maximum values. Error bars represent SEM. Statistical significance was assessed in **C**–**E**, **G**, and **H** by 2-tailed unpaired *t* test, and in **B** and **I** by 2-way ANOVA followed by Bonferroni post hoc tests for pairwise comparison. *P < 0.05, **P < 0.01.

The yield of α, β, and δ cells, assessed by immunofluorescence staining of dispersed cells at the end of the differentiation (S7 and extended culture), was comparable between the wild-type and mutant cell lines (Figure 3, A and F, and Supplemental Figure 13). By 3D light sheet fluorescence microscopy, insulin-positive volume was reduced and *TMEM167A*-mutant aggregates had decreased sphericity, pointing to potential misorganization (Figure 3, B and C).

### Transcriptome signatures of cells carrying the TMEM167A variant p.Val59Glu during β cell development.

To chart putative developmental alterations, we performed bulk RNA sequencing along the differentiation from S4 to 4-week extended culture. At S7 and extended culture, the iPSC-β cell fraction was enriched by magnetic-activated cell sorting (MACS; Miltenyi Biotec) for CD49a (31).

The highest number of differentially expressed genes was found at S5 (*n* = 251) (Supplemental Figure 14A and Supplemental Table 4). Gene set enrichment analysis identified multiple deregulated pathways (false discovery rate < 0.05) in cells carrying the p.Val59Glu variant: β cell development, insulin processing, and insulin secretion genes were upregulated at S5, with insulin secretion genes still upregulated at S6 (Supplemental Figure 15, B–D, and Supplemental Tables 9 and 10). Unfolded protein response genes were downregulated in mutant cells at S4 (Supplemental Figure 14E and Supplemental Table 8), while the ER to Golgi trafficking pathway, in which *TMEM167A* is putatively involved, was upregulated at S5 and S6 (Supplemental Figure 15A and Supplemental Tables 9 and 10). Finally, cell cycle genes were downregulated in cells carrying the variant at S4, S5, and S6 (Supplemental Figure 15B and Supplemental Tables 8–10). The replication marker Ki67, assessed by immunofluorescence staining, was not different, however, in control and mutant β cells at S5 and S6 (Supplemental Figure 16).

### The TMEM167A p.Val59Glu variant slows ER to Golgi proinsulin trafficking in β cells.

The proinsulin and insulin content of *TMEM167A*-mutant iPSC-derived β cells was reduced to approximately half of that of wild-type aggregates at S7 (Figure 3, D and E). Differentiation into more mature long-term cultured β cells reduced the proinsulin content in the wild-type aggregates to levels similar to those in *TMEM167A*-mutant cells (Figure 3G), which remained unchanged. Insulin content of mutant aggregates remained halved compared with that of wild-type aggregates (Figure 3H). The proinsulin/insulin ratio was comparable between wild-type and mutant aggregates (Supplemental Figure 17).

To evaluate whether a putative impairment in ER to Golgi trafficking caused by the p.Val59Glu variant alters proinsulin folding, the presence of misfolded proinsulin monomers was assessed by Western blot in reducing and nonreducing conditions (which break and preserve disulfide bonds, respectively) (32). No difference in the folding status of proinsulin was detected in control and *TMEM167A*-mutant iPSC-derived β cells (Supplemental Figure 18).

Next, we evaluated the role of TMEM167A in the ER to Golgi trafficking of proinsulin employing the Retention Using Selective Hooks (RUSH) assay (16) in S7 cells. In the RUSH assay, proinsulin is tagged with streptavidin-binding protein and green fluorescent protein (GFP) and retained in the ER by a streptavidin-fused hook. Upon addition of biotin, proinsulin is released, and its trafficking from the ER through the secretory pathway can be tracked by fluorescence microscopy. In a biotin chase experiment, cells fixed at different time points were analyzed for colocalization between GFP-proinsulin and *cis*-Golgi marker GM130 (Figure 3I and Supplemental Figure 19). Proinsulin and GM130 colocalization increased in wild-type β cells to more than 60% 10 and 15 minutes after biotin addition and subsequently decreased. In mutant β cells, the trafficking dynamics were altered, with decreased (40%) colocalization at 10 minutes, showing that the *TMEM167A* p.Val59Glu variant slows ER to Golgi proinsulin trafficking in β cells.

### The TMEM167A p.Val59Glu variant impairs β cell function in vitro and in vivo.

**Figure 4:**
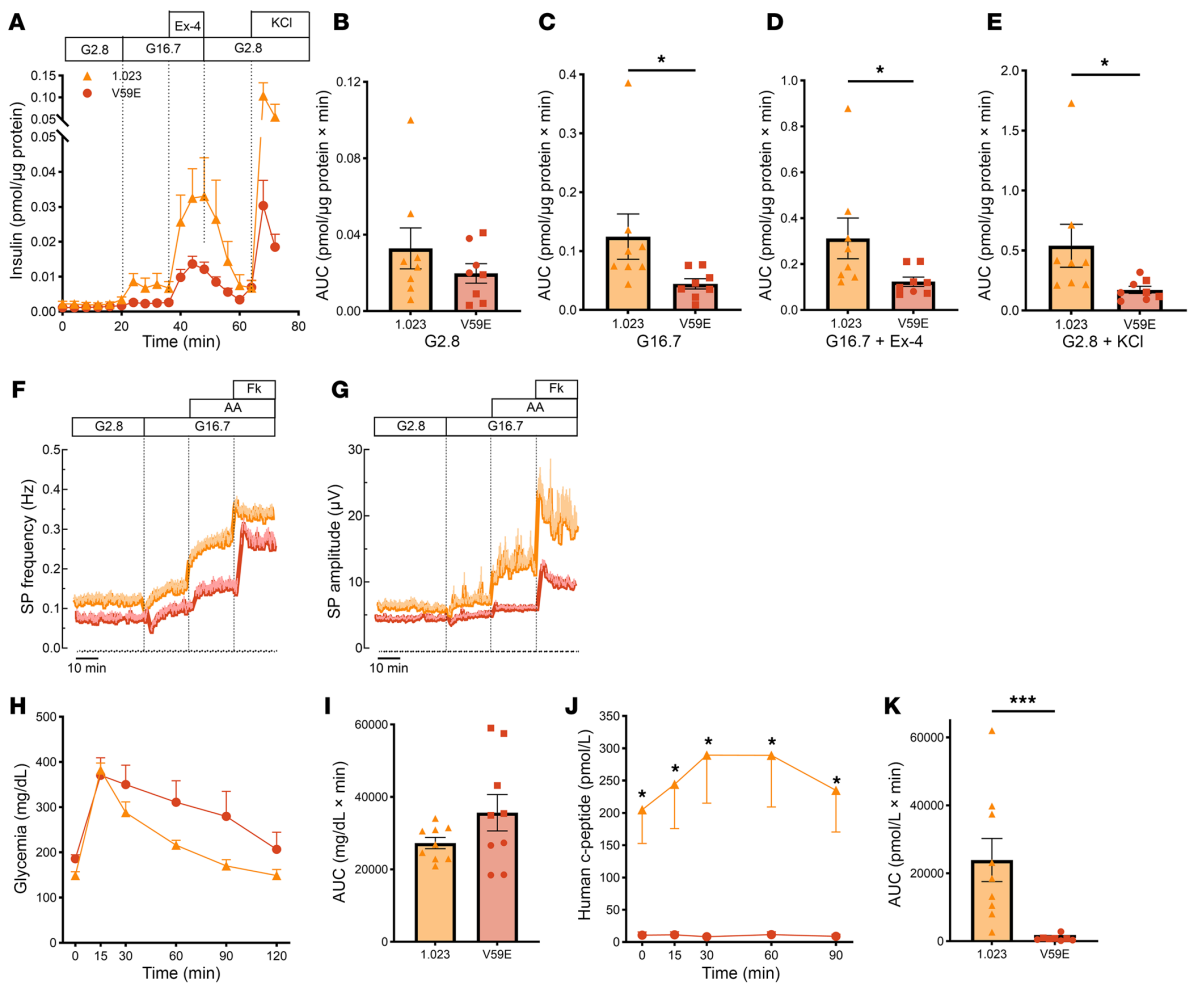
TMEM167A V59E β cells are dysfunctional. (**A**) Dynamic insulin secretion upon perifusion with 2.8 mM glucose (G2.8), followed by 16.7 mM glucose (G16.7) alone or with 12 nM exendin-4 and G2.8 plus 30 mM KCl (sampling every 4 minutes). Data are normalized to protein content (*n* = 8). (**B**–**E**) Quantification of AUC from **A** for each phase. (**F** and **G**) Kinetics of mean (± SEM) frequencies (**F**) and amplitudes (**G**) of β cell slow potentials (SPs) recorded with multi-electrode arrays, upon 2.8 mM glucose, 16.7 mM glucose, amino acid mix (AA), and forskolin (Fk; 1 μM). (**H** and **J**) Blood glucose (**H**) and plasma human C-peptide (**J**) levels during an intraperitoneal glucose tolerance test in mice 4 months after transplantation with S7 MACS-purified iPSC-β cell aggregates (*n* = 8–9). (**I** and **K**) AUC of **H** and **J**. Triangles represent mother cell line 1.023, squares 1.023 mutTMEM167A V59E.37, and circles 1.023 mutTMEM167A V59E.48. Each data point represents an independent experiment. Error bars represent SEM. Statistical significance was assessed in **C**–**E**, **I**, and **K** by Mann-Whitney test, and in **H** and **J** by 2-way ANOVA with Bonferroni’s correction. *P < 0.05, ***P < 0.001.

We next evaluated iPSC-β cell function at S7 and following long-term differentiation. In a static assay, insulin secretion stimulated by high glucose or by high glucose plus forskolin was not different between *TMEM167A*-mutant and control iPSC-derived β cells at S7 (Supplemental Figure 20). However, following long-term differentiation to generate more mature β cell aggregates, *TMEM167A*-mutant β cells showed impaired insulin secretion induced by high glucose and high glucose plus the GLP-1 receptor agonist exendin-4 in a dynamic perifusion setup. Maximal insulin secretion induced upon membrane depolarization by KCl was also significantly reduced (Figure 4, A–E).

Since mitochondria play a key role in glucose sensing, we evaluated mitochondrial function using Seahorse XFp Extracellular Flux Analyzer (Agilent). Oxygen consumption rate did not increase upon high-glucose stimulation, in keeping with earlier work showing that long-term-culture iPSC-β cells retain immature metabolic characteristics and respond better to amino acids and pyruvate than glucose (27). Control and *TMEM167A*-mutant β cells had similar basal respiration and ATP production, but cells with the variant had lower maximal respiration capacity (Supplemental Figure 21), pointing to mitochondrial dysfunction.

Downstream of metabolism, insulin secretion requires the generation of electrical signals by ion channels. We used noninvasive multi-electrode arrays to monitor at high temporal resolution the electrophysiological activity of iPSC-β cell aggregates. As a β cell–specific electrophysiological readout, we analyzed the kinetics of β cell slow potentials that reflect the synchronous activity of coupled β cells (33). Slow potentials of *TMEM167A*-mutant and control β cells had comparable frequency and amplitude at 2.8 and 16.7 mM glucose (Figure 4, F and G, and Supplemental Figure 22, A, B, E, and F). Upon stimulation with high glucose and a physiological amino acid mixture, electrical activity increased in control but not in *TMEM167A*-mutant iPSC-β cells (Figure 4F and Supplemental Figure 22C). Slow potential amplitude was lower in *TMEM167A*-mutant iPSC-β cells upon stimulation with forskolin (Figure 4G and Supplemental Figure 22H) that activates incretin-dependent pathways in β cells, in keeping with the dynamic perifusion data (Figure 4, A and D).

To achieve further functional maturation of iPSC-β cells in a permissive in vivo environment (24), we generated humanized mice by transplanting S7 iPSC-β cell aggregates under the kidney capsule of immunodeficient Rag2^–/–^ mice (34). To improve β cell function (31) and deplete non-endocrine cells with tumorigenic potential (35), we enriched β cells using CD49a MACS to 65% pure iPSC-β cell aggregates (31) before transplantation (Supplemental Figure 23). An intraperitoneal glucose tolerance test 4 months after transplantation showed a stark difference in human C-peptide secretion, which reflects insulin secretion by the human graft. While the humanized mice carrying wild-type iPSC-β cells showed glucose-stimulated human C-peptide secretion, it was barely detectable in mice with transplanted *TMEM167A*-mutant β cells (Figure 4, H–K).

### Single-cell transcriptome analysis reveals inhibited ER to Golgi trafficking and insulin secretion signatures in β cells carrying TMEM167A p.Val59Glu.

**Figure 5:**
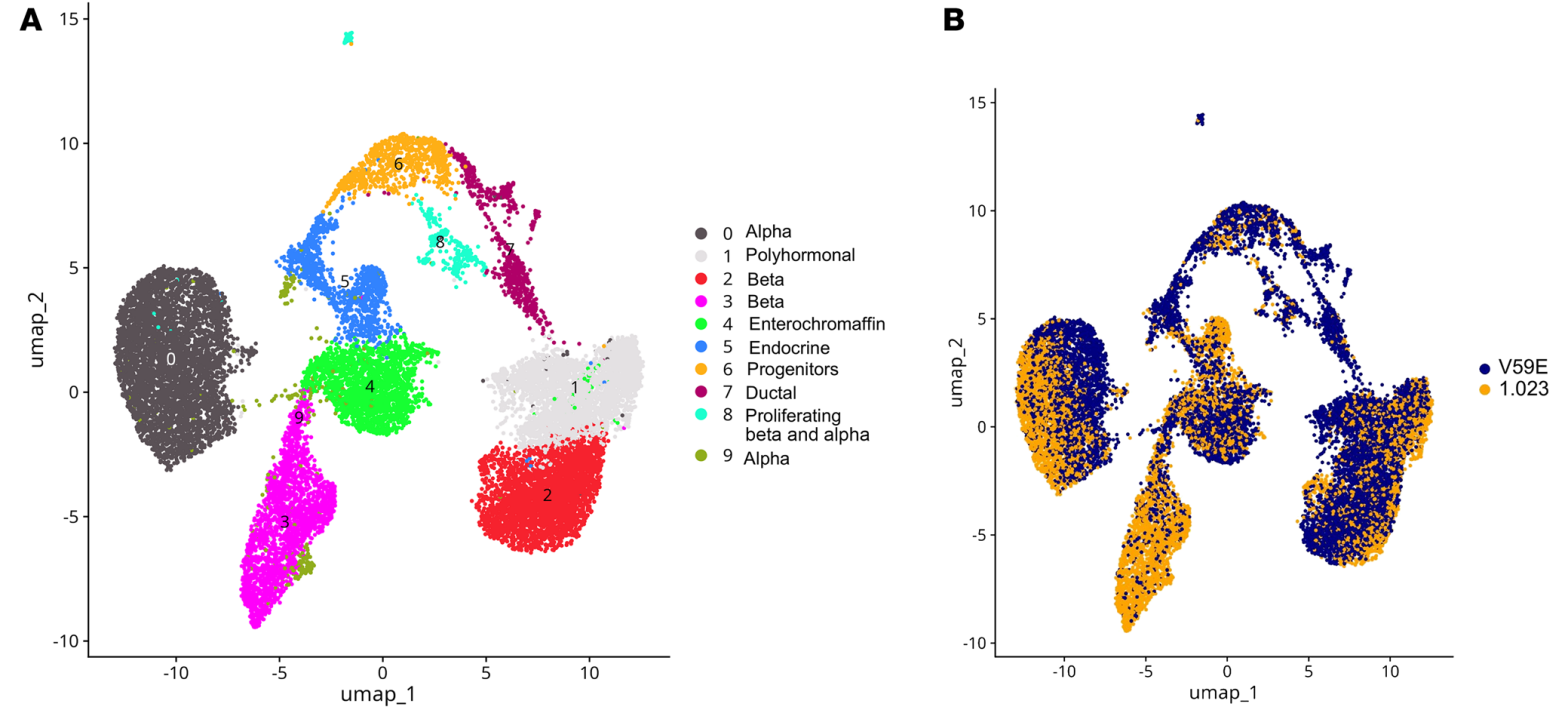
TMEM167A V59E β cells have inhibited ER to Golgi trafficking and insulin secretion gene signatures. (**A**) Clusters identified from single-cell data. 9,721 long-term culture iPSC-aggregate cells were sequenced for cell line 1.023, 9,291 cells for 1.023 mutTMEM167A V59E.37, and 4,910 cells for 1.023 mutTMEM167A V59E.48. (**B**) Fractions of wild-type and mutant cells populating different clusters.

To investigate how the *TMEM167A* p.Val59Glu variant affects maturing β cells, we performed single-cell RNA sequencing in iPSC-derived aggregates after extended culture. We identified 9 cell clusters (Figure 5A; Supplemental Figure 24 shows the genes used to annotate clusters). All pancreatic endocrine cell types were present in wild-type and mutant aggregates, suggesting that the p.Val59Glu variant does not impede islet cell fate decisions, in keeping with the α, β, and δ cell yield by immunostaining (Figure 3F). We interrogated *TMEM167A* expression in these single-cell data and in organ donor single-islet-cell data from the Human Pancreas Analysis Program (https://hpap.pmacs.upenn.edu/). *TMEM167A* expression was similar in iPSC-derived and primary β, α, and δ cells (Supplemental Figure 25). In the wild-type and mutant aggregates, however, 2 different β cell populations were detected, one mainly composed of wild-type iPSC-β cells and the other of mutant β cells (cluster 3 and cluster 2, respectively; Figure 5B). We performed an exploratory differential gene expression analysis of these 2 β cell populations (Supplemental Table 13) and examined the pathways modified in the bulk transcriptome study (Supplemental Tables 14–17). Mutant iPSC-β cells had downregulated *CREB3L2*, an ER stress transducer and regulator of protein trafficking (36), and an inhibited ER to Golgi trafficking signature, which might impair β cell function and insulin secretion. The transcription factors *RFX6* and *PAX6*, essential for β cell development and function (37–39), were also downregulated, as were the insulin processing genes *PCSK1*, *PCSK2*, and *CPE*, and genes involved in insulin secretion and exocytosis. Altogether, this transcriptomic signature points to a dysfunctional makeup of β cells carrying the *TMEM167A* p.Val59Glu variant.

### TMEM167A p.Val59Glu iPSC-β cells exhibit increased sensitivity to ER stress.

**Figure 6:**
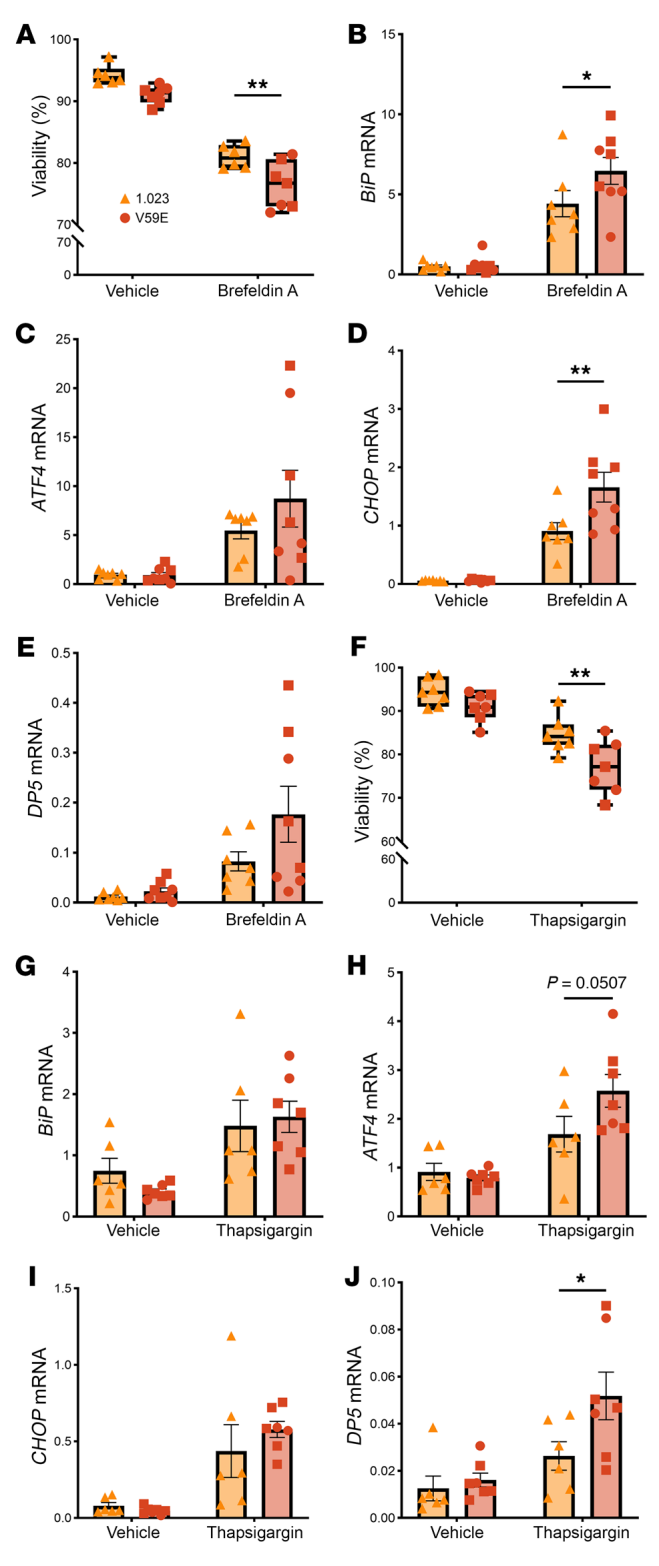
TMEM167A V59E variant–carrying β cells are sensitive to ER stress–induced apoptosis. S7 iPSC-β cell aggregates were exposed or not for 24 hours to brefeldin A (0.025 mg/dL; **A**–**E**) or for 48 hours to thapsigargin (1 μM; **F**–**J**). Viability was assessed by Hoechst 33342/propidium iodide staining (*n* = 6–7). Gene expression was assessed by qPCR, normalized to reference genes *ACTB* and *VAPA* (*n* = 6–8). Triangles represent mother cell line 1.023, squares 1.023 mutTMEM167A V59E.37, and circles 1.023 mutTMEM167A V59E.48. Error bars represent SEM. Extremities of box-and-whisker plots are maximal and minimal values; horizontal line shows median. Each data point represents an independent experiment. Statistical significance was assessed in **A**, **B**, **D**, **F**, **H**, and **J** by 2-way ANOVA with Bonferroni’s correction. *P < 0.05, **P < 0.01.

The *TMEM167A* p.Val59Glu variant did not affect basal cell viability nor increase ER stress signaling at the mRNA or protein level in S7 iPSC-β cells (Figure 6 and Supplemental Figure 26). Upon exposure to the ER stressors brefeldin A and thapsigargin, however, the viability of *TMEM167A*-mutant iPSC-β cells was significantly reduced (Figure 6, A and F). Signaling in the ER stress response was enhanced in stressed mutant cells, with increased mRNA expression of the ER stress markers *BiP*, *ATF4*, and *CHOP* and the proapoptotic BCL2 family member *DP5* (Figure 6, B–J); changes in ER stress markers were not investigated at the protein level.

Since the ER stress response is emerging as a potential therapeutic target in diabetes (40), we assessed the putative cytoprotective effects of integrated stress response inhibitor (ISRIB), imeglimin, and exendin-4. In both control and *TMEM167A*-mutant iPSC-β cells, ISRIB, imeglimin, and exendin-4 conferred protection from brefeldin A or thapsigargin (Supplemental Figure 27). ISRIB halved *CHOP* expression in brefeldin A– or thapsigargin-exposed mutant β cells (Supplemental Figure 28), in keeping with its inhibitory effect on this branch of the ER stress response.

## Discussion

We report the identification of biallelic variants in the *TMEM167A* gene in 6 individuals with clinical features of MEDS. We show that *TMEM167A* is widely expressed in developing and adult pancreas and brain (both in human and mouse). Functional studies suggest that the mechanism leading to neonatal diabetes is through β cell dysfunction and β cell sensitivity to ER stress, likely due to impaired ER to Golgi trafficking.

Our study identifies variants in *TMEM167A* as a genetic cause of MEDS, a congenital syndrome characterized by microcephaly, epilepsy, and neonatal diabetes. Identification of the genetic diagnosis in individuals with neonatal diabetes and additional features is essential to guide clinical management and inform family members of recurrence risk (41). We recommend inclusion of *TMEM167A* in gene panels used for genetic testing of neonatal diabetes and congenital microcephaly.

Pancreatic and neurological features were similar among the 6 individuals with recessive *TMEM167A* variants. All were diagnosed with neonatal diabetes and required full insulin replacement, consistent with complete β cell deficiency. All 6 individuals had microcephaly, with 5 also having epilepsy. These clinical features are similar to those reported in individuals with MEDS caused by recessive variants in *IER3IP1* and *YIPF5*. Similarly to patients with *TMEM167A* variants, individuals with MEDS caused by *IER3IP1* variants usually require lifelong insulin treatment. This was also the case for most described patients with *YIPF5*-MEDS; however, a recent report described an individual with a homozygous *YIPF5* missense variant who, in addition to microcephaly and epilepsy, had illness-induced hyperglycemia (42), with no insulin requirement between episodes. Identification of further individuals with *TMEM167A* variants will be essential to establish whether a variable diabetes phenotype is observed with this MEDS subtype.

Our results are consistent with the *TMEM167A* variants identified in our patients being loss-of-function. I-5 and I-6 were both homozygous for variants which, while not resulting in mRNA degradation through nonsense-mediated decay, were predicted to result in frameshifts and complete loss of TMEM167A function. The effect of the missense variants on TMEM167A function is difficult to predict, partly because of the poorly characterized protein structure. Topological domain predictions are ambiguous for TMEM167A and specifically for the first 30 amino acids of the 72-residue protein, where computational tools often fail to detect a signal peptide (which is essential for protein localization), predicting instead the presence of a second transmembrane domain. While the similarity in phenotype among patients in our series suggests that all *TMEM167A* variants identified in individuals with MEDS are loss-of-function, it is possible that some of the missense variants might result in a partially functional protein.

The similarity of phenotype between individuals with MEDS caused by *IER3IP1*, *YIPF5*, and *TMEM167A* variants suggests that these 3 genes may play a role in similar cellular functions. IER3IP1 and YIPF5 have been shown to be involved in protein trafficking between the ER and Golgi. *TMEM167A* encodes a small transmembrane protein localizing in the Golgi apparatus with a putative role in the secretory pathway (43). Consistent with this, in our study multiple lines of evidence support a defect in vesicular trafficking between the ER and Golgi in cells homozygous for the *TMEM167A* p.Val59Glu variant. Bulk transcriptome data showed an upregulated ER to Golgi gene signature in endocrine pancreas development (S5 and S6), while in more mature β cells single-cell RNA sequencing detected an inhibited ER to Golgi trafficking signature in cells with the p.Val59Gly variant. Furthermore, the RUSH assay directly showed impaired proinsulin trafficking in *TMEM167A*-mutant iPSC-β cells. The results of the RUSH assay are comparable to those in β cells carrying the *IER3IP1* p.Val21Gly MEDS variant (16), consistent with similar cellular functions and pathogenic mechanisms. Additional evidence comes from a recent study showing that IER3IP1 forms a complex with TMEM167A in B lymphocytes in vitro, further supporting involvement of these 2 proteins in the same cellular process (44). Overall, current evidence is consistent with a model in which variants in the 3 MEDS genes cause diabetes through impaired proinsulin trafficking due to disrupted ER to Golgi transport, resulting in sensitization of β cells to ER stress–induced apoptosis. Further studies are needed to pinpoint the exact role of each of the MEDS genes in vesicular transport and whether the transmembrane proteins encoded by *TMEM167A*, *IER3IP1*, and *YIPF5* directly interact.

Similarly to *IER3IP1* and *YIPF5*, *TMEM167A* mRNA is ubiquitously expressed in adult human tissues. The specificity of the MEDS phenotype, with only β cells and brain being overtly clinically affected, suggests that these organs are specifically sensitive to disruption of these genes and/or to impaired ER to Golgi protein trafficking. Cargo specificity has been previously suggested as a possible explanation why variants in the ubiquitously expressed *YIPF5* cause such a specific phenotype (15). The identification of a similar phenotype caused by recessive variants in *TMEM167A*, a gene encoding a protein similarly involved in the secretory pathway (43), further suggests that MEDS causative genes are involved in specific cargo transport in β cells and neurons.

*TMEM167A* is abundantly expressed in the developing brain and pancreas. This suggests that TMEM167A is required to build and maintain secretory capacity during organogenesis, working as a regulatory hub in the early secretory pathway. *IER3IP1* variants cause microcephaly by dysregulating the secretion of extracellular matrix proteins and key proteins for neuronal development and survival (45, 46). The extracellular matrix plays a crucial role in neuronal migration by providing essential signaling cues and defining the mechanical environment for neurons (47). In the brain, the extracellular matrix is primarily composed of hyaluronic acid and proteoglycans, and its composition varies across the cortical wall (48). These components contribute to progenitor self-renewal, neuronal migration, and cerebral cortex folding (49). One key extracellular matrix glycoprotein, Reelin (RELN), is crucial for proper cortical development. RELN is secreted by several cell types and orchestrates the migration and positioning of newborn projection neurons within the developing cortex. Disruption of RELN signaling causes lissencephaly, a cortical malformation characterized by an abnormal cortical lamination and a loss of the cortical folds due to defective neuronal migration and abnormal cortical layering (47, 48, 50). Interestingly, individuals I-3 and I-4 in our study were confirmed to have lissencephaly. Defects in neuronal migration are likely to also play a role in the phenotype of patients with *IER3IP1* variants resulting in a simplified gyral pattern (6) but have not been reported in individuals with *YIPF5* variants. Given TMEM167A’s involvement in early secretory trafficking, it is plausible that its dysfunction disrupts the Golgi-mediated processing or delivery of ECM-associated factors such as RELN or other guidance molecules. This may lead to impaired cortical lamination through defective migration, offering a potential mechanistic explanation for the lissencephaly phenotype. MRI scans were available only for I-3 and I4, the 2 individuals with lissencephaly. We are therefore unable to affirm or exclude whether other individuals with *TMEM167A* variants also had this brain phenotype. Further studies will be needed to confirm whether lissencephaly is a common clinical feature of MEDS caused by *TMEM167A* variants and to define the molecular mechanism.

In our model of in vitro differentiation of iPSC-derived β cells with the *TMEM167A* p.Val59Gly variant, we did not observe an obvious β cell differentiation defect. We cannot exclude that loss of *TMEM167A* could affect β cell development in vivo, since the lack of an obvious phenotype during the in vitro differentiation may be inherent to the protocols that may overly robustly drive differentiation, thereby masking developmental defects (51). It is possible that, similarly to what has been shown for *IER3IP1* in the developing brain, variants in *TMEM167A* may perturb the organization and composition of the extracellular matrix, which plays an important role in human pancreas development (52). Consistent with this, S5 bulk transcriptomes showed downregulated collagen biosynthesis and laminin and integrin interactions (Supplemental Table 9).

iPSC-derived β cells carrying the *TMEM167A* p.Val59Glu variant were dysfunctional and sensitive to ER stress, with induction of ER stress markers at the mRNA level. A limitation of the study is that this was not confirmed at the protein level. Investigation of ER stress marker protein expression will be important in future studies, as these are posttranslationally regulated in stress conditions (40). The *TMEM167A* p.Val59Glu variant halved the insulin content and insulin secretory capacity of iPSC-β cells in vitro. This may be related to impaired ER to Golgi trafficking and the direct consequence of reduced insulin content. The stimulation index of mutant and wild-type β cells was comparable, suggesting that TMEM167A does not alter insulin granule exocytosis. It is conceivable, however, that the expression of membrane proteins of the exocytotic machinery is impacted in *TMEM167A*-mutant cells; this will need to be further studied. The functional defect of the mutant β cells was exacerbated after transplantation, showing a profound deficit compared with wild-type β cells that further mature in the permissive in vivo environment. This shows the advantage of stem cell models that capture the full scale of β cell pathology. Our experiments do not disentangle whether the functional defect in vivo is due to differences in β cell maturity, failure due to increased metabolic demands on implantation caused by the higher blood glucose levels of mice compared with humans, β cell dedifferentiation, β cell death, or a combination of factors.

Both *TMEM167A* silencing and the p.Val59Glu *TMEM167A* variant sensitized β cells to ER stress–induced apoptosis in EndoC-βH1 cells and mature iPSC-β cells, respectively. ISRIB, imeglimin, and the GLP-1 receptor agonist exendin-4 improved iPSC-β cell survival, in both wild-type and mutant cells. This suggests they hold therapeutic potential for ER stress–related diabetes. This is important, since no disease-modifying treatment is currently approved for ER stress–related diabetes. While ISRIB reduced *CHOP* expression, imeglimin and exendin-4 conferred protection without altering ER stress markers, possibly owing to pleiotropic effects. Implementation of therapies aiming to pharmacologically modulate the ER stress response is currently in very early stages (40).

In conclusion, we report the identification of recessive *TMEM167A* variants as a cause of MEDS. Functional characterization of the role of *TMEM167A* in human β cells supports an essential role for this gene in ER to Golgi transport, further highlighting the importance of this process for β cell function and survival.

## Methods

### Sex as a biological variable.

Both female and male individuals were included in the genetic analyses. We used only male animals because they lack the beneficial effects of estrogens on glucose homeostasis, as we sought to evaluate the function of human iPSC-β cell grafts (53).

### Patient cohort.

Individuals with neonatal diabetes were recruited by their clinicians for genetic analysis in the Exeter Genomics Laboratory. The study was conducted following the principles of the Declaration of Helsinki with informed consent given by the patients or their parents/guardians.

### Genetic analysis.

DNA was extracted from peripheral blood leukocytes of 2 individuals diagnosed with MEDS. Pathogenic variants in known neonatal diabetes genes, including the 2 known MEDS causative genes (*IER3IP1* and *YIPF5*), were excluded. Genome sequencing was performed on an Illumina HiSeq 2500 with a mean read depth of 38.3 for I-1 and 34.2 for I-2. Genome sequencing analysis details are provided in Supplemental Methods.

Replication studies were performed in a cohort of 284 patients diagnosed with diabetes before age 6 months in whom the known genetic causes of neonatal diabetes had been excluded. This was carried out using a targeted next-generation sequencing assay (*n* = 198), which included baits for the *TMEM167A* gene, by exome sequencing (*n* = 9) or by whole-genome sequencing (*n* = 77). Variant confirmation and cosegregation in family members were performed by Sanger sequencing (Exon 1F- TAACCGCCCGTCTTGGTAG; Exon 1R-AGGGCGAGATAAGGAGTTGC, Exon2 F- TGCCAGACTTGTCATTCTGCT Exon2R- AGTAGGTTAAATCCCTCATGCGA, Exon 3F- TGCAAATTTTTGCCACTCTCAC, Exon 3R- GGAAGCCTGCCCTTTAAAAATTA, Exon 4F- TGGATCTCTGTCCTACATTCTGC, Exon 4R- CCTTTCCATTATTTTCTGCAGTCAG). Seven individuals in the replication cohort were reported to have microcephaly.

The bioinformatics tools MutationTaster (https://www.mutationtaster.org/), REVEL, and AlphaMissense (both from https://genome.ucsc.edu/) were used to predict the effect of variants on the TMEM167A protein. Variants were classified using the American College of Medical Genetics and Genomics (ACMG) guidelines (18–20).

### RNA and protein fluorescent staining, immunocytochemistry, and light sheet microscopy.

Mouse and human fetal brain, pancreas, and cerebral organoids were analyzed for *Tmem167*/*TMEM167A* mRNA expression as described in Supplemental Methods. Immunocytochemistry and light sheet microscopy of iPSC-β cell aggregates are also described in Supplemental Methods.

### iPSC CRISPR/Cas9 genome editing.

In the iPSC line 1.023, provided by D.M. Egli (Columbia University) (26), the *TMEM167A* p.Val59Glu variant was introduced using homology-directed repair, as described in Supplemental Methods.

### iPSC maintenance, differentiation into β cells, and purification by MACS.

iPSCs were cultured in Matrigel-coated plates (Corning) in E8 medium and passaged with 0.5 mM EDTA (Invitrogen Life Technologies) twice weekly. Cells were free from mycoplasma (MycoAlert, Lonza, and MycoStrip, InvivoGen), and karyotype was assessed by KaryoStat (Thermo Fisher Scientific). Pluripotency was assessed by immunocytochemistry and embryoid body assay as previously described (26). iPSCs were differentiated into β cell aggregates using a 7-stage protocol (15, 24, 26, 34). A long-term differentiation protocol was used to obtain more functionally mature β cells (27). Detailed stem cell culture, differentiation, and MACS purification protocols are provided in Supplemental Methods.

### RUSH assay.

iPSC-β cells were dispersed on Matrigel-coated glass-bottom plates (Ibidi) and transduced overnight with a lentivirus containing the RUSH construct, as previously described (16). Medium (supplemented with 15.2 nM avidin [Sigma-Aldrich] to ensure ER retention) was changed the day after. After 72 hours, 200 μM biotin was added to initiate trafficking, and cells were fixed with 4% paraformaldehyde at the indicated times. *Z*-stack images were taken with a spinning disk Aurox confocal microscope at high sectioning (0.9 μM) using a Plan-APO objective (Zeiss). Images were analyzed for co-alignment of channels, scaling, dynamic range, signal-to-noise ratio, (absence of) crosstalk and spherical aberrations, and colocalization using Huygens quality control tool (https://svi.nl/Huygens-Quality-Control) and Colocalization Analyzer.

### Cell death.

Cells were exposed to 1 μM thapsigargin or 0.025 μg/mL brefeldin A, respectively, for 24 and 16 hours for EndoC-βH1 cells, and for 48 and 24 hours for iPSC-β cells, with or without ISRIB (200 nM), imeglimin (1 mM), or exendin-4 (50 nM). Cell death was assessed by nuclear staining with Hoechst 33342 (10 μg/mL; Sigma-Aldrich) and propidium iodide (5 μg/mL; Sigma-Aldrich). Two investigators assessed cell death in at least 500 cells or 30 aggregates, one of them unaware of the experimental conditions.

### RNA extraction, qPCR, and bulk and single-cell RNA sequencing.

RNA was extracted for qPCR and bulk RNA sequencing according to details provided in Supplemental Methods. Cells were dissociated and prepped for single-cell RNA sequencing according to 10x Genomics instructions. Cell dissociation and data analyses are described in Supplemental Methods.

### Proinsulin folding, insulin content, and insulin secretion.

Proinsulin folding status was evaluated by Western blot, as described in Supplemental Methods.

Static insulin secretion by EndoC-βH1 cells was assessed as previously described (54) using Krebs solution containing 0 or 20 mM glucose or 20 mM glucose plus 10 μM forskolin.

Static insulin secretion by 50 iPSC-β cell aggregates was assessed in 24-well plates after 90-minute preincubation in Krebs solution containing 2.8 mM glucose. They were sequentially incubated for 30 minutes in Krebs solution containing 2.8 and 16.7 mM glucose and 16.7 mM glucose plus 10 μM forskolin. Dynamic insulin secretion by 50 aggregates was assessed with the PERI5LM perifusion system (Biorep Technologies) with a flow rate of 0.1 mL/min, sampling every 4 minutes. Aggregates were pre-perifused for 90 minutes with 2.8 mM glucose Krebs solution, and then perifused with Krebs solution containing 2.8 and 16.7 mM glucose and 16.7 mM glucose plus exendin-4 (12 nM) and KCl (30 mM).

Cells were lysed, insulin extracted using acid ethanol (95% ethanol, 5% 12N HCl), and protein content measured by Bradford assay. Insulin was measured by ELISA (Mercodia) and normalized to protein content.

### Electrophysiological analysis with multi-electrode arrays.

iPSC-β cell aggregates were dispersed on Matrigel-coated multi-electrode arrays. Cells were left to reaggregate, medium was changed after 3 days, and recordings were performed after 4–5 days. A detailed description of the method is available in Supplemental Methods.

### iPSC-β cell transplantation and metabolic studies in mice.

Mice housed in the animal facility on a 12-hour light/12-hour dark cycle had ad libitum access to regular chow. Eight- to twelve-week-old male B6.Cg-Rag2tm1.1Cgn/J mice (The Jackson Laboratory) were anesthetized by intraperitoneal injection of ketamine (100 mg/kg; Nimatek, Dechra) and xylazine (5 mg/kg; Rompun, Bayer). 1,200 to 1,800 MACS-purified iPSC-β cell aggregates were transplanted under the left kidney capsule, using a catheter and syringe or by insertion of the aggregates clotted with a blood drop from the recipient mouse. Intraperitoneal glucose tolerance tests were done after 6 hours of fasting. Glycemia was measured using a glucometer (Accu-Chek Aviva Nano, Roche) before and 15, 30, 60, 90, and 120 minutes after glucose injection (2 mg/g body weight). Blood was collected from the tail vein at 0, 15, 30, 60, and 90 minutes and plasma separated by centrifugation at 3,000*g* for 20 minutes at 4°C. Plasma C-peptide was quantified using human ultrasensitive C-peptide ELISA (Mercodia).

### Statistics.

Data are shown as bars, with the line representing the mean, or box and violin plots, with the line representing the median, 25th and 75th percentiles at the bottom and top, and whiskers representing minimum and maximum values. Individual points represent independent experiments. Comparisons between groups were performed by 1- or 2-way ANOVA with Dunnett’s, Tukey’s, or Bonferroni’s correction for multiple comparison, or by Kruskal-Wallis test. Comparison between columns was performed by 2-tailed unpaired *t* test or Mann-Whitney test. Normality was assessed by Shapiro-Wilk test. Statistical analyses were performed with GraphPad Prism 10.2.2. *P* values less than 0.05 were considered significant.

### Study approval.

The genetic studies were approved by the North Wales Research Ethics Committee (517/WA/0327). Human fetal pancreatic tissues (GW9 and GW13 + 5 days) were provided by the INSERM Human Developmental Cell Atlas Biobank. Maternal written consent was obtained and approved by the INSERM Ethics Committee, agreement IRB00003888 (Saint-Denis La Plaine, France). Human fetal brain experiments were approved by the Comité d’Ethique Hospitalo-Facultaire Universitaire de Liège (EudraCT: 2017-002366-45). Mouse experiments were approved by the Commission d’Ethique et du Bien Être Animal, Faculty of Medicine, Université Libre de Bruxelles, following the European Convention for the Protection of Vertebrate Animals used for Experimental and other Scientific Purposes (European Treaty Series No. 123). Human iPSCs were differentiated into β cells with approval of the Ethical Committee of Erasmus Hospital, Université Libre de Bruxelles (P2019/498, A2024/211).

### Data availability.

The Supporting data values file is provided together with the manuscript. Clinical and genotype data are available only through collaboration, as this can be used to identify individuals and so cannot be made openly available. Requests for collaboration will be considered following an application to the Genetic Beta Cell Research Bank (https://www.diabetesgenes.org/current-research/genetic-beta-cell-research-bank/). Contact by email should be directed to EDF. All requests for access to data will be responded to within 28 days. Single-cell transcriptomic data for wild-type and *TMEM167A-*mutant iPSC aggregates are available from the NCBI’s Gene Expression Omnibus under accession GSE302421; bulk transcriptomic data for wild-type and *TMEM167A*-mutant iPSC lines were deposited under accession GSE302570. This article used data from the database (https://hpap.pmacs.upenn.edu/) of the Human Pancreas Analysis Program (HPAP; RRID:SCR_016202). HPAP is part of a Human Islet Research Network (RRID:SCR_014393) consortium (UC4-DK112217, U01-DK123594, UC4-DK112232, and U01-DK123716).

## Author contributions

EDF, MC, and ATH designed the study. EV, ATH, MC, PA, MR, LN, and EDF planned the experiments. EV, TS, CV, HM, RL, AA, AL, FNDS, ML, FF, and SH performed the functional assessment in β cells. GB, MG, MBJ, SEF, and EDF analyzed the genetic data. MNW, JRS, and TWL performed bioinformatic analysis for genome sequencing and targeted next-generation sequencing data. TP, XY, and AP performed bioinformatic analyses for bulk and single-cell RNA sequencing. MIE and BJS established the genome editing on iPSCs. ST, FSN, RS, and LN planned and performed the expression analysis in human and mouse pancreas and brain. ATH, PB, JS, RG, DH, NS, SA, BÖD, BÖ, FM, IMT, MTA, OSA, HAA, and NK analyzed the clinical data. EV, EDF, and MC wrote the first draft of the manuscript. All authors reviewed and improved the manuscript.

## Funding support

This research was supported by the NIHR Exeter Biomedical Research Centre and the NIHR Exeter Clinical Research Facility. The views expressed are those of the authors and not necessarily those of the NIHR or the Department of Health and Social Care. ATH is employed as a core member of staff within the National Institute for Health Research–funded Exeter Clinical Research Facility and is an NIHR Emeritus Senior Investigator.

- National Institute for Health and Care Research (NIHR) Exeter Biomedical Research Centre.
- NIHR Exeter Clinical Research Facility.
- Wellcome Trust, 224600/Z/21/Z.
- Diabetes UK/Breakthrough T1D RD Lawrence Fellowship, 23/0006516, to MBJ.
- Wellcome Trust Senior Research Fellowship, 223187/Z/21/Z, to SEF.
- Diabetes UK RD Lawrence Fellowship, 19/005971, to EDF.
- European Foundation for the Study of Diabetes/Novo Nordisk Foundation Future Leader Award to EDF.
- Research Foundation Flanders (FWO) and Fund for Scientific Research–Fonds National de la Recherche Scientifique (FNRS) Excellence of Science project Pandarome to MC.
- Walloon Region strategic axis Fonds de la Recherche Scientifique–Walloon Excellence in Life Sciences and Biotechnology (WELBIO) to MC.
- FNRS to MC.
- FNRS-Weave to MC.
- Win4Excellence GT4Health to MC.
- Fondation ULB to MC
- Société Francophone du Diabète to AL.
- European Union Horizon Health project NEMESIS to MC.
- FNRS-Fund for Research Training in Industry and Agriculture (FRIA) fellowship to EV.
- Société Francophone du Diabète to MR and RL.
- French Ministry of Education and Research to MR and RL.

## Supplementary Material

Supplemental data

Unedited blot and gel images

Supplemental tables 1-17

Supporting data values

## Figures and Tables

**Table 1:**
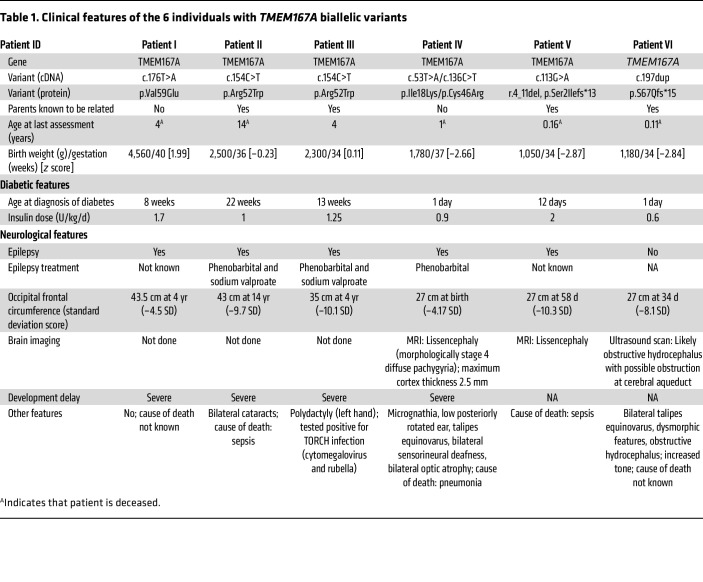
Clinical features of the 6 individuals with *TMEM167A* biallelic variants

## Version 1 09/09/2025

In-Press Preview

## Version 2 11/17/2025

Electronic publication
