# Supplementary material for: Recessive TMEM167A variants cause neonatal diabetes, microcephaly, and epilepsy syndrome
Source: J Clin Invest. 2025 Sep 9;135(22):e195756. doi: 10.1172/JCI195756 (PMC12618065; doi:10.1172/JCI195756)
Supplement: Supplemental data [file jci-135-195756-s251.pdf]

Supplemental Materials and Methods

Genome sequencing analysis

The sequencing data were analyzed using an approach based on the GATK best practice guidelines. GATK HaplotypeCaller (https://gatk.broadinstitute.org/hc/en-us/articles/360037225632-HaplotypeCaller) was used to identify variants that were annotated using Alamut batch version 1.8 (Interactive Biosoftware), and variants that failed the QD2 VCF filter or had less than 5 reads supporting the variant allele were excluded. SavvyCNV, which uses read depth to judge copy number states, was used to call copy number variants. SavvyVcfHomozygosity was used to identify large (>3 Mb) homozygous regions in the genome sequencing data (https://github.com/rdemolgen/SavvySuite).

Human ESC-derived cerebral organoids

The ESC line H9 was differentiated into cerebral organoids according to published protocols (1) (2). ESCs were dissociated using Accutase (Stem Cell Technologies). 9,000 single cells were plated per well of round-bottomed ultra-low attachment 96-well plates (Thermo Fisher Scientific) and cultured in embryoid body medium DMEM/F12 with GlutaMAX (Thermo Fisher Scientific, 10565018), 20% knockout serum replacement (Thermo Fisher Scientific, 10828028), 1% MEM non-essential amino acids (Thermo Fisher Scientific, 11140035), 0.1 mM β-mercaptoethanol (Thermo Fisher Scientific), 4 ng/ml bFGF (Thermo Fisher Scientific, 100-18B) and 50 μM Y-27632 (ROCK inhibitor, Selleck, S1049). On day 6, embryoid bodies were transferred into 6-well plates for neural induction in DMEM/F12 medium containing GlutaMAX supplement, 1% N-2 supplement (Thermo Fisher Scientific, 17502048), 1% MEM non-essential amino acids and 1 μg/ml heparin (Sigma-Aldrich, H3149). On day 11, embryoid bodies were embedded in Matrigel (Corning, cat. no. 354234) and maintained in neural induction medium for an additional 2 days. On day 13, the medium was replaced with differentiation medium, which consisted of DMEM/F12 with GlutaMAX supplement, 50% Neurobasal medium (Thermo Fisher Scientific, 21103049), 0.5% N-2 supplement, 1% B27 without vitamin A (Thermo Fisher Scientific, 12587010), 0.5% MEM non-essential amino acids, 2 μg/ml human insulin and 0.05 mM β-mercaptoethanol. On day 18, Matrigel-embedded organoids were transferred into a spinning bioreactor. On day 20, medium was replaced with differentiation medium containing 1% B27 with vitamin A (Thermo Fisher Scientific, 17504044).

Detailed β cell differentiation protocol

Prior to the differentiation of iPSCs, cells were expanded in 10-cm diameter Matrigel-coated dishes. When cells reached 80% confluency, SCs were washed with 0.5 mM EDTA, dissociated in single cells by incubation with Accutase (Capricorn Scientific) for 3-5 min, pelleted (250 g for 3-min centrifugation) and resuspended at a density of 0.8-1 million cells/mL in E8 medium supplemented with 5 μM ROCK inhibitor (Y-27632 dihydrochloride, StemCell technologies) prior to seeding in

Matrigel-coated 6-well plate (3 mL/well) or 10-cm dish (15 mL/dish). Twenty-four hours later, (day 0, definitive endoderm induction, 2D cell culture) when cells reached full confluency, they were rinsed once with PBS and 2 ml/12ml of day 0 medium was added per well/dish.

During days 1 to 12, comprising stages S1 (days 1-3), S2 (days 4-6), S3 (days 7-8), S4 (days 9-12), until pancreatic progenitor stage (2D cell culture), 2 mL/12 mL of medium (6-well plate or 10-cm dish) was refreshed every day according to the differentiation stage (Fig. S8). At days 3 and 12, cells were fixed with 4% paraformaldehyde to verify by immunohistochemistry the expression of endodermal (SOX17) and pancreatic progenitor (PDX1 and NKX6.1) markers, respectively. At day 0 (D0), day 8 (S3), and day 12 (S4), cell pellets were collected to assess the expression of beta cell differentiation markers by qPCR. Briefly, cells were rinsed twice with PBS, detached using a cell scraper and stored at -80°C until RNA extraction.

At day 13, cells were transferred to 3D culture. Cells were washed with 0.5 mM EDTA, incubated with Accutase for 6-8 min at 37°C, collected and pelleted by 3 min centrifugation at 250 g. Cells were resuspended in S5 medium (Supplementary Table S25) supplemented with 10 µM ROCK inhibitor and plated in 24-well microwell plates (2 mL/well, AggreWell400, StemCell technologies).

From day 13 to 16 (S5, until endocrine precursor stage, 3D culture), 1 mL medium/well was refreshed every day. A cell pellet was collected on day 16.

On the first day of S6, cells were transferred from microwell to suspension culture on ultralow attachment plates (Corning) placed on an orbital shaker (96 r.p.m. rotational speed).

From day 17 until the end of the differentiation, corresponding to stages S6 (7 days), S7 (7-8 days), and long-term culture (3-4 weeks), the medium (5 mL/suspension well) was refreshed every 2-3 days and cell pellets were collected at the end of each stage. On the last day of S7 and extended culture, islets were dispersed and seeded on a chamber slide to quantify the proportion of insulin-, glucagon-, and somatostatin-positive cells by immunocytochemistry.

Basal media composition and specific stage media are described in Supplementary Tables 25 and 26.

Assessment of the effect of the c.113G>A variant on splicing

A fragment containing *TMEM167A* exon 2, flanked by 945 bp of the upstream and downstream introns, was cloned into the pSPL3 vector by NovoPro Bioscience inc. The c.113G>A variant in Exon 2 was generated by site directed mutagenesis by NovoPro Bioscience inc. WT and mutant plasmid were transformed into DH5α Competent Cells (Thermo Fisher scientific) and grown on agar plates containing ampicillin (Sigma-Aldrich). Individual colonies were grown up in LB broth (Sigma-Aldrich) and plasmid DNA was extracted by the ZymoPURE II™ Plasmid Midiprep kit (Zymo Research). Sanger sequencing was performed to confirm the presence of WT/Mutant TMEM167a exon 2.

250,000 HEK 293 cells were seeded in a 6 well plate on day 0. 5 µg of WT/Mutant pSPL3 was transfected on day 1 using Opti-MEM and lipofectamine 3000 following the supplier instructions (Thermo Fisher Scientific). Total RNA was extracted 48 hours after transfection using Trizol and Chloroform (Thermo Fisher Scientific). Genomic DNA was digested using ezDNase according to the manufacturer's instructions (Thermo Fisher Scientific). RNA was reverse transcribed into cDNA using the SuperScript™ IV First-Strand Synthesis System kit (Thermo Fisher Scientific) with the SA2 primer. cDNA was amplified by PCR with the SD2 and SA4 primers. PCR products were run on a 2% agarose gel alongside a DNA ladder (Bench Top PCR Marker, Promega). Gel bands were extracted using the GenElute™ Gel Extraction Kit (Sigma-Aldrich) and fragments were Sanger sequenced.

EndoC-βH1 cell culture and RNA interference

EndoC-βH1 cells were kindly provided by R. Scharfmann (Université Paris Cité, Institut Cochin, INSERM) and cultured as described (3) (4). *TMEM167A* was silenced using 2 siRNAs targeting a different sequence (si2 SI04321751 and si4 SI404345747, Qiagen). Allstar Negative Control siRNA (Qiagen) was used as negative control. Transfection was performed using 30 nmol siRNA and Lipofectamine RNAiMAX (Thermo Fisher Scientific) as described (5). Transfection was performed overnight, and cells were cultured 48h before subsequent use.

RNA and protein fluorescent staining

Mouse and human fetal brain and pancreas and cerebral organoids were fixed with 4% PFA overnight at 4°C, dehydrated with 30% sucrose and cryosectioned at a thickness of 14 µm except for human brain (20 µm) and cerebral organoids (12 µm). RNA staining using a probe targeting mouse Tmem167/human TMEM167A RNA (Bio-Techne) was carried out following the RNAscope manufacturer's protocol. Primary antibodies were incubated overnight at 4°C. Antibodies are listed in Supplementary Table 20, and positive and negative control probes in Supplementary Table 21.

iPSC CRISPR/Cas9 genome editing

The guide RNA to generate the mutation (ATACAGCATACTGCAACATA) was designed using Benchling (https://benchling.com). Two silent mutations were introduced to reduce the likelihood of the gRNA binding after a successful editing. The first and third off-target predicted by CRISPOR were sequenced to rule out off-target editing (6); the second was not due to difficulties in generating adequate primers. Primers and the HDR template used in genome editing are provided in Supplementary Tables 18 and 19. Ribonucleoprotein components HiFi Cas9 protein, crRNA and tracrRNA (Integrated DNA Technologies) were prepared according to the manufacturer's instructions. Two million iPSCs were electroporated with the ribonucleoprotein complex (Neon Transfection System, 1100 mV, 20 ms, 2 pulses), plated on Matrigel-coated plates in StemFlex medium (Thermo Fisher Scientific) with 10 µM ROCK inhibitor Y-27632 Dihydrochloride (STEMCELL

Technologies), and cultured at 32°C for 48h. HDR enhancer (ready-to-use, 1.7 µl/ml, Integrated DNA Technologies) was added to the medium for the first 24h. Afterwards, cells were single-cell seeded and expanded in E8 medium (Thermo Fisher Scientific) at 37°C. Successful editing was confirmed by Sanger sequencing for 2 different clones V59E.37 and V59E.48.

Immunocytochemistry

Immunocytochemistry on single cells was performed as previously described (7). Whole iPSC-β cell aggregates were fixed in 4% paraformaldehyde for 1h, included in agarose and paraffinized. Sections were deparaffinized and incubated in Tris-citrate buffer plus 0.05% Tween-20 for 15 min at 90°C, blocked for 30 min in TBST plus 3% BSA and incubated with primary antibodies overnight at 4°C and secondary antibody incubation for 1h at room temperature. Slides were washed three times in TBST, the first wash containing DAPI. Antibodies are listed in Supplementary Table 23.

Western blot

iPSC-β cell aggregates were lysed in Laemmli buffer. Total protein concentration was determined by Bramhall assay, and ~5 µg was prepared in SDS buffer, divided in two equal portions, to which 200 mM DTT was added (reducing) or not (nonreducing). Samples were resolved on 4–12% Bis-Tris NuPAGE gel (Invitrogen) at 200 V for 35 min. Semi-dry electrotransfer of proteins to nitrocellulose membrane was done using a Hoefer semi-phor electrotransfer unit (150 mA for 60 min). Post-transfer, the nitrocellulose membrane was rinsed in TBST (15 mM Tris, 150 mM NaCl, 0.1% Tween-20), blocked using TBST plus 5% BSA for 1h at room temperature, and washed four times (5 min each) in TBST. This was followed by incubation with primary (proinsulin, insulin, β-actin, overnight) and secondary antibodies (1h, room temperature), with four TBST washes after each incubation. Immunoblots were developed using enhanced chemiluminescence (Bio-Rad Clarity) and images captured using a Fotodyne gel imager. The antibody list is available in Supplementary Table 22.

RNA extraction and qPCR

Total RNA was extracted from snap-frozen mouse and human fetal brain using the Trizol-chloroform method. Reverse transcription was performed using the RevertAid H Minus First Strand cDNA Synthesis Kit (Thermo Fisher Scientific) to generate cDNA. qPCR was done using BRYT Green dye and GoTaq 2X Master Mix (Promega) on the LightCycler 480 (Roche). Mouse Tmem167 mRNA was normalized against average expression of reference genes Gapdh, Ppia, 36b4, using $\Delta\Delta$Ct method. mRNA level at E12.5 was set as the reference (normalized to 1). Human TMEM167A was normalized against average expression of reference genes PPIA and HPRT using GW11 cortex as the reference (normalized to 1). For β cells, RNA was extracted with Poly(A)+-RNA oligo-dT 25-coated polystyrene Dynabeads (Life Technologies), following the manufacturer's instructions. RNA was reverse transcribed using Reverse Transcriptase Core Kit (Eurogentec). qPCR was performed using SYBR

Green Supermix on CFX Connect (Bio-Rad). Expression was normalized to the geometric mean of reference genes VAPA and ACTB. Primer sequences are provided in Supplementary Table 24.

Bulk RNA-sequencing

Total RNA was extracted from 1.023, TMEM167A V59E.37 and V59E.48 iPSC-β cell differentiation stages S4, S5, S6, S7 and long-term culture (n=4 per stage and per iPSC line) using RNeasy Plus Micro Kit (Qiagen, Venlo, Netherlands), according to the manufacturer's recommendation. For the library preparation, all samples had at least 200 ng high-quality RNA with RNA integrity number (RIN) higher than 7, as calculated by Agilent Bioanalyzer. The RNA-seq was performed on NovaSeq X Plus (Novogene GmbH).

Raw RNA-seq data were assessed for sequencing quality using fastp 0.20.1 (8) to trim adapters, correct low-quality bases, filter-out low-quality reads and infer sample contamination by recording the GC content for each read. Sequencing depth of filtered reads was between 190 and 300 million reads. Gene expression was quantified using Salmon version 1.4.0 (9) setting parameters to correct for sequence-specific and fragment-level GC biases and to validate the produced mapping. GENCODE version 38 (GRCh38.p13) (10) was selected as the reference human genome for the quasi-alignment and indexed with the default k-mer values.

Differential gene expression between TMEM167A mutant and 1.023 control cells was analysed with R package DESeq2 version 1.38.3 (11). Estimated gene abundance from Salmon was imported to DESeq2. For each gene comparison, a log2 fold change value was associated with a Wald test statistic and a p-value, corrected using false discovery rate (FDR) by Benjamini-Hochberg method (Supplementary Tables 3-7). Genes with an FDR <0.05 were considered significantly differentially expressed.

Functional enrichment analysis was conducted on the Wald test statistic for all genes after differential analysis using fGSEA version 1.29.1 (12) R package in combination with gene sets provided by REACTOME (13) (Supplementary Tables 8-12). Pathways with Benjamini-Hochberg FDR <0.05 were considered significant.

Bulk RNA-seq data was obtained from 16 non-diabetic donors (8F/8M; age 56.8 ±22.1 years) from (14). Transcripts per Million (TPM) for *TMEM167A* was extracted and normalized by the expression of reference genes *ACTB* and *VAPA*.

Raw RNA-seq FASTQ files from FACS-sorted human α and β cells were retrieved from the Human Pancreas Analysis Program portal (https://hpap.pmacs.upenn.edu) (15) (16). Quality control was performed with fastp v0.19.6 (17), including adapter trimming and removal of low-quality bases. To reduce inter-donor variability, we selected 12 non-diabetic donors with paired α and β cell samples (4F/8M; age 35.3 ±10.8 years). Cleaned reads were aligned to the human transcriptome (GENCODE v36, GRCh38.p13) using Salmon v1.4.0 (9), with --seqBias, --gcBias, and --validateMappings

enabled to correct for technical biases. Transcript-level estimates were imported into R via tximport (18) and summarized to gene-level TPM values using the summarizeToGene function. TPM for *TMEM167A* was extracted and normalized by the expression of reference genes *ACTB* and *VAPA*.

Single cell RNA-sequencing

Extended culture (S7 plus 5 weeks) iPSC-β cell aggregates were washed twice with EDTA and dissociated with Accumax (Sigma Aldrich) for 16 min at 37°C. Cells were washed twice in 1% BSA PBS, centrifuged at 300 g for 5 min, filtered using a cell strainer capped tube with 35 μM nylon mesh (Corning, US), counted and adjusted to a 1000 cell/μL concentration. Cell number and viability were evaluated by two researchers using a haemocytometer, after staining with 0.4% Trypan blue (Sigma Aldrich). Between 4910 to 9721 cells were loaded per experiment into the 10× Chromium Single Cell Platform (10X Genomics) using the Next GEM Single Cell 3′ library and Gel Bead Kit (v3.1 chemistry) according to manufacturer's instructions. The RNA-seq was performed on NovaSeq 6000 (ULB-VUB BRIGHTCore).

For the analyses, we used 10x Genomics Cell Ranger v9.0.1 (19) and Seurat v5.2.1 (20), to identify and annotate the clusters. scDblFinder v1.12.0 (17) was employed for doublet/multiplet handling, and SoupX v1.6.2 (21) for removing cell free RNA. Cluster' annotation was made using genes representing the major cell types and potential contaminants. To assess the effect of *TMEM167A* p.Val59Glu, differential gene expression analysis was performed using using logistic regression, implemented through Seurat's wrapper function (Supplementary Table 13).

Raw single-cell RNA-seq FASTQ files of islets from 14 non-diabetic donors (age 21.9 ±12.8 years) were obtained from the HPAP portal (15) (16). Sequencing reads were processed with the Cell Ranger count pipeline (19), including alignment to the GRCh38 human genome, barcode and UMI assignment, and generation of gene-by-cell expression matrices. To mitigate ambient RNA contamination, we applied SoupX v1.6.1 (21), leveraging marker genes (INS, GCG, SST, TTR, IAPP, PYY and KRT19) identified from initial clustering to estimate and correct for background RNA. The decontaminated count matrices were subsequently processed in Seurat v4.3.0 (22). Quality control retained cells meeting the following criteria: (1) ≥200 detected genes per cell, with each gene expressed in ≥3 cells; (2) exclusion of doublets identified using scDblFinder v1.12.0 (17); (3) <9,000 total gene counts and <10,000 UMI counts per cell; and (4) ≤5% mitochondrial gene content. Gene expression was normalized using Seurat's SCTransform, accounting for sequencing depth and regressing out mitochondrial gene proportion. The top 3,000 variable genes were selected for principal component analysis. To correct batch effects arising from donor identity and reagent kits, we integrated the datasets using Harmony v1.2.0 (23), based on the top 50 principal components. Subsequently, a, b, and δ cells were extracted for final analysis. To determine the optimal number of principal components for downstream projection, we applied an elbow plot approach, selecting

the top 10 principal components that accounted for ~80% of the variance. Uniform Manifold Approximation and Projection (UMAP) was then performed to visualize cellular heterogeneity.

Mitochondrial respiration

Oxygen consumption rate of iPSC-β cells was measured using XFp Extracellular Flux Analyzer (Seahorse Bioscience) (24) and normalized to basal level at 2.8 mM glucose.

Electrophysiological analysis with multi-electrode arrays

iPSC-β cell aggregates were were washed with Dulbecco's PBS -/- (17-516F; LONZA) and 0.5 mM EDTA (Life technologies). Aggregates were twice incubated with Accutase (A6964; Sigma) for 4 min at 37°C followed by gentle mechanical dissociation. The reaction was stopped with Knock-out Serum Replacement (Life technologies) supplemented with 10 µM Rock inhibitor (Y-27632 Dihydrochloride; STEMCELL Technologies). Cells were resuspended in culture medium supplemented with Rock inhibitor and seeded at 50 million cells/cm² on the 60 microelectrodes of 5% Matrigel-coated (BD Biosciences) multi- electrode arrays (60PedotMEA200/30iR-Au; MultiChannel Systems) containing medium supplemented with 2% Matrigel and 10% FBS. Cells reaggregated in 118±24 µm diameter clusters, medium was changed after 3 days, and recordings performed after 4-5 days.

Extracellular potentials were recorded at 37°C in buffer containing (in mM): NaCl 135, KCl 4.8, $MgCl_2$ 1.2, $CaCl_2$ 1.8 et HEPES 10 (pH 7.35). Electrical activity on each electrode was amplified and recorded using a USB-MEA60-Inv-System-E acquisition system (gain: 1200), with a sampling rate of 10 kHz and analogic bandwidth of 0.1 to 3000 Hz. Slow potentials (0.1-2 Hz) and action potentials (20-3000 Hz) were isolated and analysed using MC_Rack software (v4.6.2) (25). The physiological amino acid mixture was composed of 19 amino acids as in (26), containing 0.88 mM Ala, 0.38 mM Arg, 0.076 mM Asp, 0.19 mM Cit, 0.24 mM Glut, 0.6 mM Gly, 0.15 mM His, 0.19 mM Ile, 0.32 mM Leu, 0.74 mM Lys, 0.1 mM Met, 1.4 mM Orn, 0.16 mM Phe, 0.7 mM Pro, 1.14 mM Ser, 0.54 mM Thre, 0.15 mM Trp, 0.4 mM Val, 2 mM Glut, and was diluted to 10.4 mM before incubation.

Magnetic activated cell sorting

S7 iPSC-β cell aggregates were dissociated with Accumax, incubated at room temperature for 21 min with anti-human CD49a PE-conjugated antibody (BD Bioscience) (27), protected from light and agitated. After 2 washes with MACS buffer (PBS, 1% BSA, 2 mM EDTA), cells were incubated for 15 min at 4°C with anti-PE UltraPure MACS microbeads (Miltenyi Biotec) under constant agitation. Cells were washed, magnetically sorted on LS columns (Miltenyi Biotec) on a MACS separator, resuspended in 500 µl MACS buffer in a pre-washed column, washed 3 times with 500 µl MACS buffer, and removed from the separator. The first wash was sorted again on a second column, sorted cells pooled and plated in Aggrewell plates (Stemcell Technologies) to reaggregate.

Light-sheet microscopy

1-h 4% paraformaldehyde-fixed iPSC-β cell aggregates were permeabilized with WILD buffer (PBS, 5% DMSO, 5 µg/mL heparin, 0.05% Tween-20, 2% Triton X-100, 0.3 M Glycine) for 1h and then blocked with WILD buffer plus 3% donkey serum for 1 day. They were incubated with primary and secondary antibodies in blocking solution for 2-3 days at room temperature under agitation, with 3 washes with WILD buffer after each incubation. They were included in agarose, dehydrated, delipidated in dichloromethane twice for 15-30 min and cleared in dibenzyl ether at least overnight. Aggregate acquisition was done with an UltraMicroscope II Light Sheet Fluorescence Microscope magnification 3.2X and dipping cap 2X (final magnification of 6.4X). Aggregate volume image was acquired using tissue autofluorescence (488 nm laser excitation; 525/50 nm emission filter) with a z-step of 4 µm; endocrine marker acquisition was done with a z-step of 4 µm (561 nm laser excitation and 620/60 nm emission filter for glucagon; 640 nm excitation and 680/30 nm emission for somatostatin; 785 nm excitation and 845/55 nm emission for insulin). Tiff images were converted to Imaris compatible files using Imaris file Converter software. Each channel of the 3D image was segmented using Imaris Surface tool, using an intensity threshold adapted for each image depending on marker preservation, to ensure high-fidelity segmentation. Spurious objects were filtered out by rejecting objects smaller than 10 voxels. The 3D image was then smoothed using a surface detail of 2 µm. The contribution of each channel to total volume of reconstructed objects was calculated by normalizing the volume of each endocrine marker to the total aggregate volume (by autofluorescence). Antibodies are listed in Supplementary Table 23.

Table S18. Sequence of primers used for iPSC genome editing

Target	Forward primer (5'→3')	Reverse primer (5'→3')
TMEM167A p.Val59Glu mutation screening	TTCGTACAAAGGAAAGCATCCA	AGTACTGAGGTTGATTGTTACAGAC
Off-target number 1	TAAGTTATGTCTCATTTACCTTCCTCC	GTCTATTGACCATAATCTTGCTGCT
Off-target number 3	GCACTCAGATCAATTTCCTCAGC	GTGCCCTGTAAACTGCTAAACTC

Table S19. HDR donor template for iPSC genome editing

	Sequence
HDR	GTTCACAATCAAATCTGATGGCAACTACATTCTGGCATTTTCCCCAGCTACTGTATGAAGAGGATGCTGAAGGCCATTACTATACAGCATtCAGCGACATAAGGACTCTTCCGTTCACCTGTTGAAAAAAA

Table S20. Antibodies used for RNAscope and protein fluorescent staining

Antibody	Dilution	Reference
Goat anti-mouse SOX2	1:200	Santa Cruz Cat#sc-17320, RRID: AB_2286684
Mouse anti-mouse TBR2	1:200	Thermo FIsher Scientific Cat#14-4875-82, RRID: AB_11042577
Rabbit anti-mouse/human TBR1	1:200	Abcam Cat#ab31940, RRID: AB_2200219
Mouse anti-human SOX2	1:200	Santa Cruz Cat# sc-365823, RRID: AB_10842165
Rat anti-human CTIP2	1:200	Abcam Cat#ab18465, RRID: AB_2064130
Rabbit anti-mouse/human SOX9	1:200	Santa Cruz Cat# sc-20095, RRID: AB_661282
Mouse anti-mouse/human insulin	1:200	Sigma Aldrich Cat#I2018, RRID: AB_260137
Mouse anti-human SOX2	1:200	BD Biosciences Cat#561469, RRID: AB_10694256
Alexa Fluor™ 647 Donkey anti-Rabbit IgG (H+L) Highly Cross-Adsorbed Secondary Antibody	1:500	Thermo Fisher Scientific Cat#A-31573, RRID: AB_2536183
Alexa Fluor™ 555 Donkey anti-Mouse IgG (H+L) Highly Cross-Adsorbed Secondary Antibody	1:500	Thermo Fisher Scientific Cat#A-31570, RRID: AB_2536180

Alexa Fluor™ 647 Donkey anti-Mouse IgG (H+L) Highly Cross-Adsorbed Secondary Antibody	1:500	Thermo Fisher Scientific Cat#A-31571, RRID: AB_162542
Alexa Fluor™ 555 Donkey anti-Rabbit IgG (H+L) Highly Cross-Adsorbed Secondary Antibody	1:500	Thermo Fisher Scientific Cat#A-31572, RRID: AB_162543
Alexa Fluor™ Plus 647 Donkey anti-Rat IgG (H+L) Highly Cross-Adsorbed Secondary Antibody	1:500	Thermo Fisher Scientific Cat#A-48272, RRID: AB_2893138
Alexa Fluor™ 555 Donkey anti-Goat IgG (H+L) Cross-Adsorbed Secondary Antibody	1:500	Thermo Fisher Scientific Cat#A-21432, RRID: AB_141788
Alexa Fluor™ Plus 647 Donkey anti-Rat IgG (H+L) Highly Cross-Adsorbed Secondary Antibody	1:500	Thermo Fisher Scientific Cat#A48272, RRID: AB_2893138

Table S21. Negative and positive control probes for RNAscope

Probe	Reference
RNAscopeTM 3-plex Negative Control Probe - RNAscopeTM Negative control probe DapB (of Bacillus subtilis strain) for RNAscopeTM Multiplex Fluorescent Assay.	Advanced Cell Diagnostic, 320871
RNAscopeTM 3-plex Positive Control Probe - Mm - RNAscopeTM Mouse positive control probe for RNAscopeTM Multiplex Fluorescent Assay - Polr2a (C1 channel) and PPIB (C2 channel), UBC (C3 channel) Note: In terms of relative expression levels, UBC is the highest, PPIB is considered a moderate-high, POLR2A is moderate-to-low expressor target.	Advanced Cell Diagnostic, 320881
RNAscopeTM 3-plex Positive Control Probe - Hs - RNAscopeTM Human positive control probe for RNAscopeTM Multiplex Fluorescent Assay-Polr2a (C1 channel) and PPIB (C2 channel), UBC (C3 channel) In terms of relative expression levels,UBC is the highest, PPIB is considered a moderate-high, POLR2A is moderate-to-low expressor target.	Advanced Cell Diagnostic, 320861

Table S22. Antibodies used for western blot

Antibody	Dilution	Reference
Guinea pig anti-human insulin	1:1000	Covance, RRID: AB_10013624

Rat anti-human proinsulin	1:1000	CCI-17, Novus, RRID :AB_1107982
Mouse anti-beta actin	1:1000	Proteintech, RRID: AB_2883475
HRP-goat anti-guinea pig	1:5000	BioRad, RRID: AB_2535546
HRP-goat anti-mouse	1:5000	BioRad, RRID: AB_1706516
HRP-goat anti-rabbit	1:5000	BioRad, RRID: AB_1721079

Table S23. Antibodies used for immunocytochemistry and light sheet microscopy

Antibody	Dilution	Reference
Rabbit anti-human OCT4	1:400	Cell Signaling Technology Cat# 2840 RRID: AB_2167691
Goat anti-human SOX17	1:500	R and D systems Cat# AF1924 RRID: AB_355060
Mouse anti-human NKX6.1	1:250	BD Biosciences Cat#563022 RRID: AB_2737958
Goat anti-human PDX1	1:500	R and D Systems Cat# AF2419, RRID: AB_355257
Guinea pig anti-human insulin	Ready to use	Agilent Cat# A056401-2, RRID: AB_2617169
Mouse anti-human glucagon	1:1000	Sigma-Aldrich Cat# G2654, RRID: AB_259852
Rabbit anti-human somatostatin	1:1000	Abcam Cat#ab108456, RRID: AB_11158517
Rabbit anti-human BiP	1:500	Cell Signaling Technology Cat#3177S, RRID: AB_2119845
Rabbit anti-human vimentin	1:800	Abcam Cat#137321, RRID: AB_2921312
Mouse anti-human betaIII tubulin	1:500	Promega Cat#G1721, RRID: AB_430874
Mouse anti-human SSEA-4	1:500	Thermofisher Scientific, Cat#MA1-021, RRID: AB_2536687
Mouse anti-human TRA-1-60	1:100	Thermofisher Scientific, Cat#MA1-023, RRID: AB_2536699
Rabbit anti-human Nanog	1:400	Cell Signalling, Cat#4903, RRID: AB_10559205
Mouse anti-human glucagon	1:800	Sigma-Aldrich Cat# G2654, RRID: AB_259852
Goat anti-GFP	1:400	Abcam Cat#ab6673, RRID: AB_305643
Mouse anti-human GM130	1:750	BD Biosciences Cat#610822, RRID: AB_398142
Rabbit anti-human PDI	1:250	Cell Signaling Cat#3501, RRID: AB_2156433
Rabbit anti-human somatostatin	1:800	Abcam Cat#ab108456, RRID: AB_11158517

Alexa Fluor® 488 AffiniPure™ Donkey Anti-Rabbit IgG (H+L)	1:1000	Jackson ImmunoResearch Cat#711-545-152, RRID: AB_2313584
Alexa Fluor® 488 AffiniPure™ Donkey Anti-Mouse IgG (H+L)	1:1000	Jackson ImmunoResearch Cat#715-545-151, RRID: AB_2340846
Rhodamine Red™-X (RRX) AffiniPure™ Donkey Anti-Mouse IgG (H+L)	1:1000	Jackson ImmunoResearch Cat#715-295-151, RRID: AB_3095481
Rhodamine Red™-X (RRX) AffiniPure™ Donkey Anti-Goat IgG (H+L)	1:1000	Jackson ImmunoResearch Cat#705-295-147, RRID: AB_3095466
Alexa Fluor® 488 AffiniPure™ Donkey Anti-Guinea Pig IgG (H+L)	1:1000	Jackson ImmunoResearch Cat#706-545-148, RRID: AB_2340472
Alexa Fluor® 647 AffiniPure™ Donkey Anti-Rabbit IgG (H+L)	1:1000	Jackson ImmunoResearch Cat#711-605-152, RRID: AB_2492288
Rhodamine Red™-X (RRX) AffiniPure™ Donkey Anti-Rabbit IgG (H+L)	1:1000, 1:500	Jackson ImmunoResearch Cat#711-295-152, RRID: AB_3095472
Alexa Fluor® 488 AffiniPure™ Donkey Anti-Goat IgG (H+L)	1:500	Jackson ImmunoResearch Cat#705-545-147, RRID: AB_2336933
Alexa Fluor® 647 AffiniPure™ Donkey Anti-Mouse IgG (H+L)	1:500	Jackson ImmunoResearch Cat#715-605-151, RRID: AB_2340863
Alexa Fluor® 790 AffiniPure™ $F(ab')_2$ Fragment Donkey Anti-Guinea Pig IgG (H+L)	1:400	Jackson ImmunoResearch Cat#706-656-148, RRID: AB_2922884
Donkey F(ab')2 Anti-Mouse IgG H&L (Alexa Fluor® 568)	1:400	Abcam Cat#ab175699, RRID: AB_3094502
Alexa Fluor® 647 AffiniPure™ $F(ab')_2$ Fragment Donkey Anti-Rabbit IgG (H+L)	1:400	Jackson ImmunoResearch Cat#711-606-152, RRID: AB_2340625

Table S24. Sequence of mouse (m) and human (h) primers used for qPCR

Gene	Forward primer (5’→3’)	Reverse primer (5’→3’)
mGapdh	AGGTCGGTGTGAACGGATTTG	TGTAGACCATGTAGTTGAGGTCA
m36b4	ATGGGTACAAGCGCGTCCTG	GCCTTGACCTTTTCAGTAAG
mPpia	CCACTGTCGCTTTTCGCCGC	TGCAAACAGCTCGAAGGAGACGC

mTmem167	ACCGGCCTCACTATGTCTGC	GGTGCCAGGGATCGGATATAAG
hPPIA	CCAACACAAATGCTTCCCAGT	CCATGGCCTCCACAATATTCA
hHPRT	ATGGACAGGACTGAACGTCTT	CTTGAGCACACAGAGGGCTA
hTMEM167A (1)	GTGTGCCAGAATTGGTGAACGG	CTGGCATTTTCCCCAGCTACT
hACTB	CTGTACGCCAACACAGTGCT	GCTCAGGAGGAGCAATGATC
hVAPA	TACCGAAACAAGGAAACTAATGGAA	GCCTTAAACCTTCATCTCTCAGGT
hCHOP	Qiagen quantitect primer 0002278	Qiagen quantitect primer 0002278
hBiP	Qiagen quantitect primer 00096404	Qiagen quantitect primer 00096404
hDP5	GAGCCCAGAGCTTGAAAGG	CCCAGTCCCATTCTGTGTTT
hPUMA	TTGTGCTGGTGCCCGTTCCA	AGGCTAGTGGTCACGTTTGGCT
hATF4	GGCCAAGCACTTCAAACCTC	GAGAAGGCATCCTCCTTGCT
hPDX1	AAAGCTCACGCGTGGAAA	GCCGTGAGATGTACTTGTTGA
hNKX6.1	GGGCTCGTTTGGCCTATT	CGTGCTTCTTCCTCCACTT
hINS	CCAGCCGCAGCCTTTGTGA	CCAGCTCCACCTGCCCCA
hGCG	GCTAAACAGAGCTGGAGAGTAT	AAGCCCTCTTTGGGAACTT
hSST	GTTTGACCAGCCACTCTCCAG	TACTTGGCCAGTTCCTGCTTCC
hTMEM167A (2)	TATTCGATCCTTGGCACCCA	TCCGTTCACCAATTCTGGCA
hSOX9	ATCAAGACGGAGCAGCTGAG	GGCTGTAGTGTGGGAGGTTG
hNGN3	GACGACGCGAAGCTCACCAA	TACAAGCTGTGGTCCGCTAT

Table S25 Basal media composition

**Basal 1**	MCDB131 (Gibco), 2 mM GlutaMAX (Gibco), 1.5 g/L NaHCO3 (Merck Millipore), 0.5% BSA fraction V (Sigma-Aldrich), 10 mM glucose (Sigma-Aldrich)
**Basal 2**	MCDB131 (Gibco), 2 mM GlutaMAX (Gibco), 2.5 g/L NaHCO3 (Merck Millipore), 2% BSA fraction V (Sigma-Aldrich), 10 mM glucose (Sigma-Aldrich), 1:200 ITS-X (Thermo Fisher Scientific)

**Basal 3**	MCDB131 (Gibco), 2 mM GlutaMAX (Gibco), 1.5 g/L NaHCO3 (Merck Millipore), 2% BSA fraction V (Sigma-Aldrich), 20 mM glucose (Sigma-Aldrich), 1:200 ITS-X (Thermo Fisher Scientific), 10 µg/mL heparin (STEMCELL Technologies), 10 µM zinc sulfate (Sigma-Aldrich), 1X penicillin streptomycin (Lonza)
**Basal long-term culture**	CMRL1066 (Gibco), 2 mM GlutaMAX (Gibco), 2% BSA fraction V (Sigma-Aldrich), 1:200 ITS-X (Thermo Fisher Scientific), 10 µg/mL heparin (STEMCELL Technologies), 10 µM zinc sulfate (Sigma-Aldrich), 1X penicillin streptomycin (Lonza), 0.5 mM Sodium Pyruvate (Gibco), 1:2000 Trace elements A (Corning), 1:2000 Trace elements B (Corning), 1:2000 CD Lipid concentrate (Gibco)

Table S26. Stage-specific media composition

**D0**	Basal 1, 100 ng/mL Activin A (PreproTech), 5 µM CHIR-99021 (Axon Medchem)
**D1**	Basal 1, 100 ng/mL Activin A (PreproTech), 0.5 µM CHIR-99021 (Axon Medchem)
**D2**	Basal 1, 100 ng/mL Activin A (PreproTech)
**S2**	Basal 1, 0.25 mM L-ascorbic acid (Sigma-Aldrich), 50 ng/mL FGF7 (PreproTech)
**S3**	Basal 2, 0.25 mM ascorbic acid (Sigma-Aldrich), 50 ng/mL FGF7 (PreproTech), 0.25 µM SANT-1 (Sigma-Aldrich), 1 µM retinoic acid (Sigma-Aldrich), 100 nM LDN-193189 (Selleckchem), 200 nM TPB (Santa Cruz)
**S4**	Basal 2, 0.25 mM L-ascorbic acid (Sigma-Aldrich), 50 ng/mL FGF7 (PreproTech), 0.25 µM SANT-1 (Sigma-Aldrich), 0.1 µM retinoic acid (Sigma-Aldrich), 200 nM LDN-193189 (Selleckchem), 100 ng/mL EGF (STEMCELL Technologies), 10 mM nicotinamide (Sigma-Aldrich), 10 ng/mL Activin A (PreproTech)
**S5**	Basal 3, 0.05 µM retinoic acid (Sigma-Aldrich), 0.25 µM SANT-1 (Sigma-Aldrich), 100 nmM LDN-193189 (Selleckchem), 1 µM GC-1 (Tocris), 100 nM GSiXX (Merck Millipore), 10 µM ALK5 inhibitor II (ENZO), 20 ng/mL betacellulin (PreproTech)
**S6**	Basal 3, 100 nM LDN-193189 (Selleckchem), 10 µM ALK5 inhibitor II (ENZO), 1 µM GC-1 (Tocris), 100 nM GSiXX (Merck Millipore)
**S7**	Basal 3, 1 µM GC-1 (Tocris), 10 µmM Trolox (Sigma-Aldrich), 20 µM SP600125 (Selleckchem), 75 µM resvetratrol (Sigma-Aldrich), 2 µM R428 (STEMCELL Technologies), 1 mM N-acetyl-cysteine (Sigma-Aldrich)
**Long term culture**	Basal long-term culture, 0.5 µM ZM447439 (Selleckchem), 10 nM Tri-iodothyronine (T3), 1 mM N-acetyl-cysteine (Sigma-Aldrich)

Supplementary References

1. Lancaster MA, and Knoblich JA. Generation of cerebral organoids from human pluripotent stem cells. *Nature Protocols.* 2014;9(10):2329-40.
2. Giandomenico SL, Sutcliffe M, and Lancaster MA. Generation and long-term culture of advanced cerebral organoids for studying later stages of neural development. *Nature Protocols.* 2021;16(2):579-602.
3. Ravassard P, Hazhouz Y, Pechberty S, Bricout-Neveu E, Armanet M, Czernichow P, et al. A genetically engineered human pancreatic beta cell line exhibiting glucose-inducible insulin secretion. *J Clin Invest.* 2011;121(9):3589-97.
4. Coomans de Brachene A, Castela A, Op de Beeck A, Mirmira RG, Marselli L, Marchetti P, et al. Preclinical evaluation of tyrosine kinase 2 inhibitors for human beta-cell protection in type 1 diabetes. *Diabetes Obes Metab.* 2020;22(10):1827-36.
5. Moore F, Colli ML, Cnop M, Esteve MI, Cardozo AK, Cunha DA, et al. PTPN2, a candidate gene for type 1 diabetes, modulates interferon-gamma-induced pancreatic beta-cell apoptosis. *Diabetes.* 2009;58(6):1283-91.
6. Haeussler M, Schonig K, Eckert H, Eschstruth A, Mianne J, Renaud JB, et al. Evaluation of off-target and on-target scoring algorithms and integration into the guide RNA selection tool CRISPOR. *Genome Biol.* 2016;17(1):148.
7. De Franco E, Lytrivi M, Ibrahim H, Montaser H, Wakeling MN, Fantuzzi F, et al. YIPF5 mutations cause neonatal diabetes and microcephaly through endoplasmic reticulum stress. *J Clin Invest.* 2020;130(12):6338-53.
8. Chen S, Zhou Y, Chen Y, and Gu J. fastp: an ultra-fast all-in-one FASTQ preprocessor. *Bioinformatics.* 2018;34(17):i884-i90.
9. Patro R, Duggal G, Love MI, Irizarry RA, and Kingsford C. Salmon provides fast and bias-aware quantification of transcript expression. *Nature Methods.* 2017;14(4):417-9.
10. Frankish A, Carbonell-Sala S, Diekhans M, Jungreis I, Loveland Jane E, Mudge Jonathan M, et al. GENCODE: reference annotation for the human and mouse genomes in 2023. *Nucleic Acids Research.* 2023;51(D1):D942-D9.
11. Love MI, Huber W, and Anders S. Moderated estimation of fold change and dispersion for RNA-seq data with DESeq2. *Genome Biology.* 2014;15(12):550.
12. Sergushichev AA. An algorithm for fast preranked gene set enrichment analysis using cumulative statistic calculation. *bioRxiv.* 2016:060012.
13. Jassal B, Matthews L, Viteri G, Gong C, Lorente P, Fabregat A, et al. The reactome pathway knowledgebase. *Nucleic Acids Research.* 2020;48(D1):D498-D503.
14. Marselli L, Piron A, Suleiman M, Colli ML, Yi X, Khamis A, et al. Persistent or Transient Human beta Cell Dysfunction Induced by Metabolic Stress: Specific Signatures and Shared Gene Expression with Type 2 Diabetes. *Cell Rep.* 2020;33(9):108466.
15. Kaestner KH, Powers AC, Naji A, Consortium H, and Atkinson MA. NIH Initiative to Improve Understanding of the Pancreas, Islet, and Autoimmunity in Type 1 Diabetes: The Human Pancreas Analysis Program (HPAP). *Diabetes.* 2019;68(7):1394-402.
16. Shapira SN, Naji A, Atkinson MA, Powers AC, and Kaestner KH. Understanding islet dysfunction in type 2 diabetes through multidimensional pancreatic phenotyping: The Human Pancreas Analysis Program. *Cell Metabolism.* 2022;34(12):1906-13.
17. Germain PL, Lun A, Garcia Meixide C, Macnair W, and Robinson MD. Doublet identification in single-cell sequencing data using scDblFinder. *F1000Res.* 2021;10:979.
18. Soneson C, Love MI, and Robinson MD. Differential analyses for RNA-seq: transcript-level estimates improve gene-level inferences. *F1000Res.* 2015;4(2046-1402 (Print)):1521.
19. Zheng GXY, Terry JM, Belgrader P, Ryvkin P, Bent ZW, Wilson R, et al. Massively parallel digital transcriptional profiling of single cells. *Nature Communications.* 2017;8(1):14049.

20. Hao Y, Stuart T, Kowalski MH, Choudhary S, Hoffman P, Hartman A, et al. Dictionary learning for integrative, multimodal and scalable single-cell analysis. *Nature Biotechnology.* 2024;42(2):293-304.
21. Young MD, and Behjati S. SoupX removes ambient RNA contamination from droplet-based single-cell RNA sequencing data. *GigaScience.* 2020;9(12):giaa151.
22. Hao Y, Hao S, Andersen-Nissen E, Mauck WM, Zheng S, Butler A, et al. Integrated analysis of multimodal single-cell data. *Cell.* 2021;184(13):3573-87.e29.
23. Korsunsky I, Millard N, Fan J, Slowikowski K, Zhang F, Wei K, et al. Fast, sensitive and accurate integration of single-cell data with Harmony. *Nature Methods.* 2019;16(12):1289-96.
24. Gorgogietas V, Rajaei B, Heeyoung C, Santacreu BJ, Marin-Canas S, Salpea P, et al. GLP-1R agonists demonstrate potential to treat Wolfram syndrome in human preclinical models. *Diabetologia.* 2023;66(7):1306-21.
25. Jaffredo M, Bertin E, Pirog A, Puginier E, Gaitan J, Oucherif S, et al. Dynamic Uni- and Multicellular Patterns Encode Biphasic Activity in Pancreatic Islets. *Diabetes.* 2021;70(4):878-88.
26. Zhu L, Dattaroy D, Pham J, Wang L, Barella LF, Cui Y, et al. Intraislet glucagon signaling is critical for maintaining glucose homeostasis. *JCI Insight.* 2019;4(10).
27. Veres A, Faust AL, Bushnell HL, Engquist EN, Kenty JH, Harb G, et al. Charting cellular identity during human in vitro beta-cell differentiation. *Nature.* 2019;569(7756):368-73.

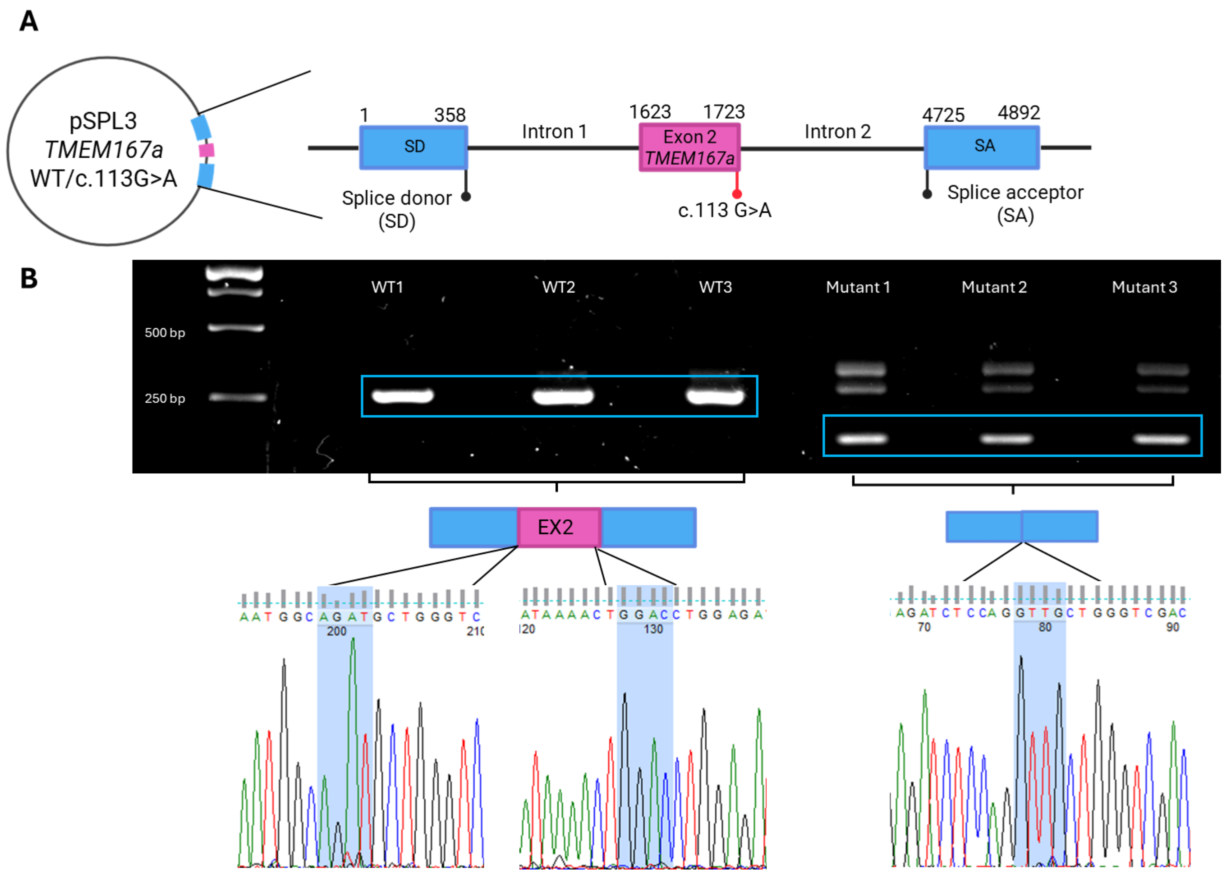

Figure S1: Assessment of the effect of the *TMEM167A* c.113G>A variant on splicing.

(A) Schematic representation of the pSPL3 vector used for the exon trapping assay. The number of nucleotides in each exon are indicated above with the splice donor and splice acceptors labelled below. (B) 2% agarose gel showing the splice products obtained from HEK293 cells transfected with the WT or c.113G>A pSPL3 vector. Underneath is a schematic representation of what exons are presents within each band indicated within the blue boxes confirmed by Sanger sequencing. Highlighted regions within the sanger trace represent the Exon-Exon junction of each band.

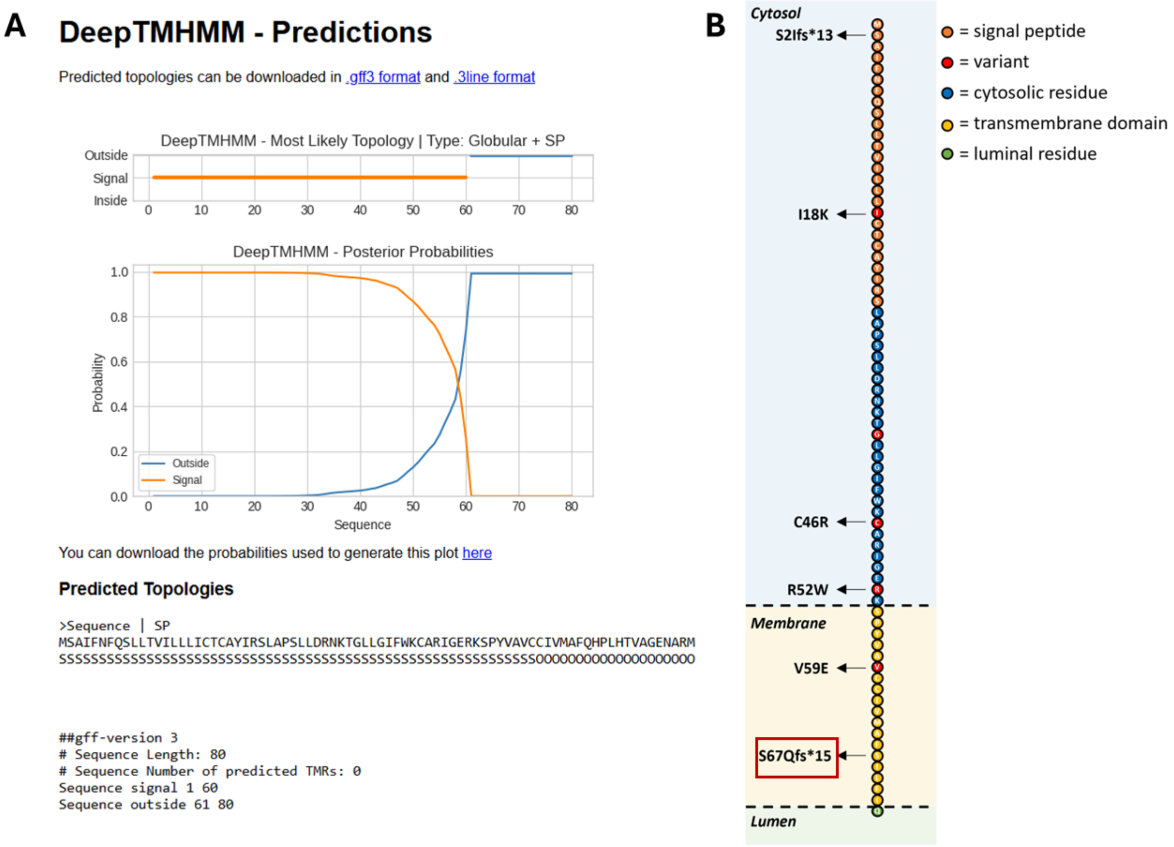

Figure S2: Predicted effect of the *TMEM167A* p.S67Qfs*15 variant according to deepTMHMM.

(A) DeepTMHMM (https://dtu.biolib.com/DeepTMHMM) output for the protein sequence resulting from the *TMEM167A* p.S67Qfs*15 variant. The resulting protein is predicted to not localise to the membrane. (B) Schematic representation of the TMEM167A transmembrane protein with variant positions indicated by black arrows. Domains are represented as predicted in UniProtKB (https://www.uniprot.org/). The red rectangle highlights the position of the p.S67Qfs*15 variant.

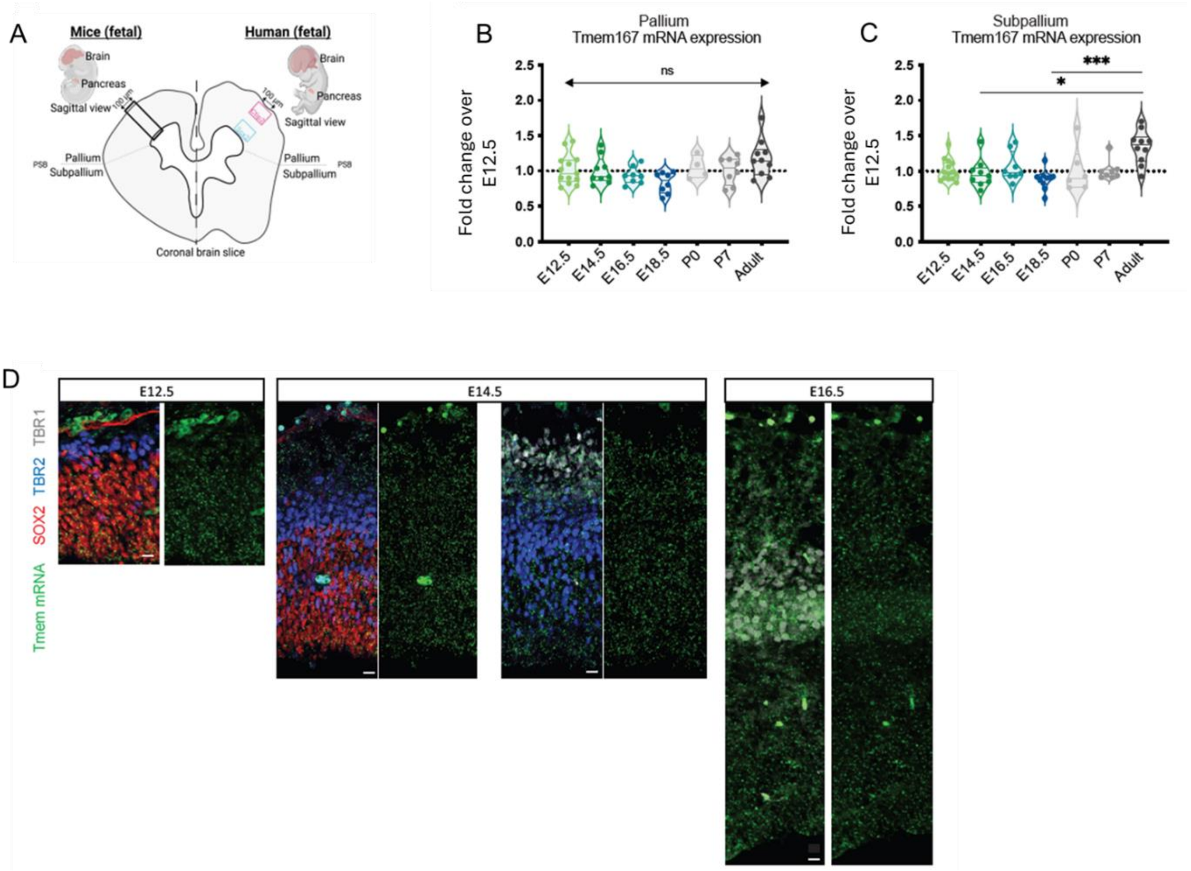

Figure S3: Expression pattern of *Tmem167* in mouse brain

(A) Schematic representation of a sagittal view of mouse and human embryo and coronal mouse and human fetal brain section, with corresponding 100 µm bin used for further immunolabeling. (B, C) Quantification of *Tmem167* mRNA expression of pallium and subpallium at different embryonic (E) and postnatal (P) ages in the mouse brain. The expression level at E12.5 was set as 1.0 and other ages are expressed relative to this stage. Violin plots show median ± quartiles in between four and fourteen pallium and subpallium biological replicates. (D) Immunolabeling of E12.5, E14.5 and E16.5 mouse cortex showing expression of *Tmem167* mRNA (green), Tbr1 (grey), Tbr2 (blue) and Sox2 (red). Scale bars, 10 µm. Statistical significance was assessed by Kruskal-Wallis ANOVA test in B and C

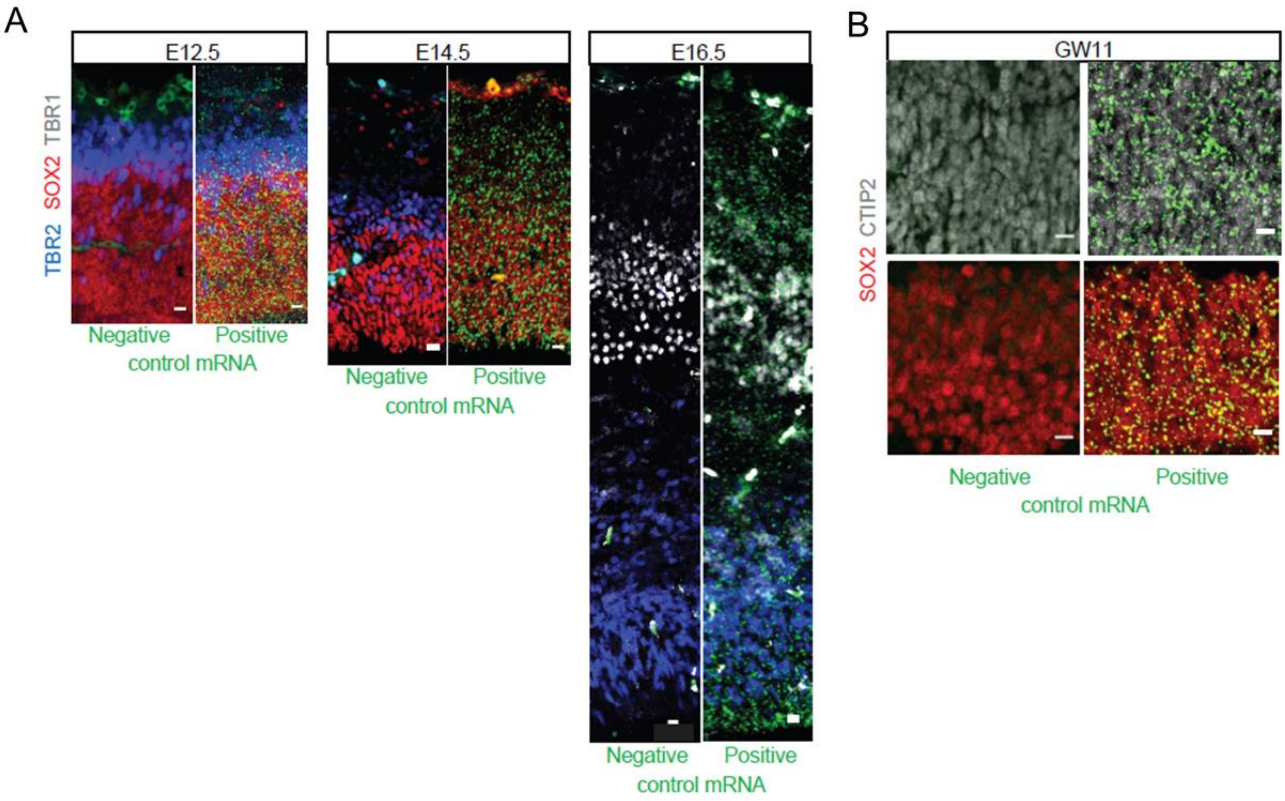

Figure S4: Expression patterns of positive and negative control probes in mouse and human fetal brain tissue.

(A) Immunolabeling of E12.5, E14.5 and E16.5 mouse cortex showing expression of negative or positive control mRNA (green), TBR1 (grey), TBR2 (blue) and SOX2 (red). Scale bars 10 µm. (B) Immunolabeling of GW11 cortex showing expression of negative or positive control mRNA (green), CTIP2 (grey) and SOX2 (red). Scale bars 10 µm. Negative and positive control probes are described in Supplementary Table 21.

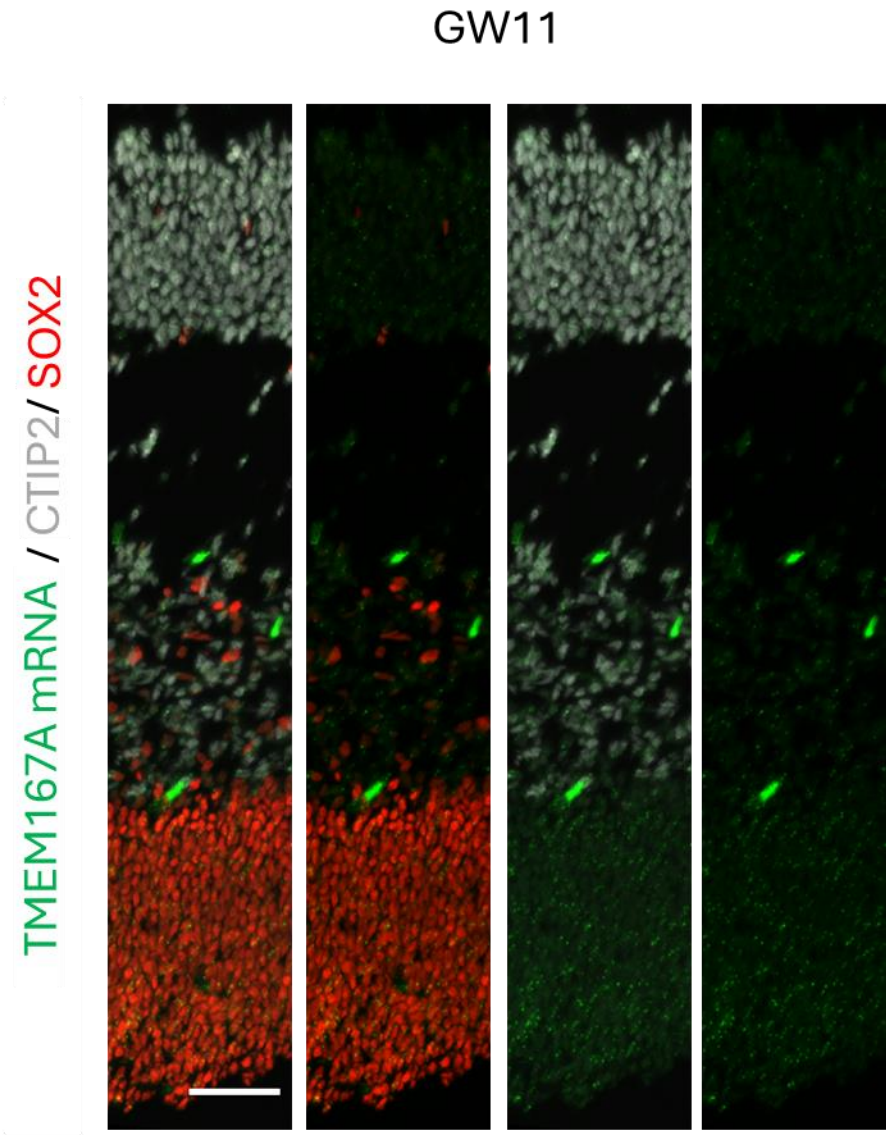

Figure S5: ***TMEM167A*** **expression in CTIP2 and SOX2 positive cells in GW11 human brain.**

RNAscope and immunolabeling of GW11 cortex showing expression of *TMEM167A* mRNA (green), CTIP2 (grey) and SOX2 (red). Scale bars 50 µm. Data shown here represent a different visualization of those presented in Figure 2C.

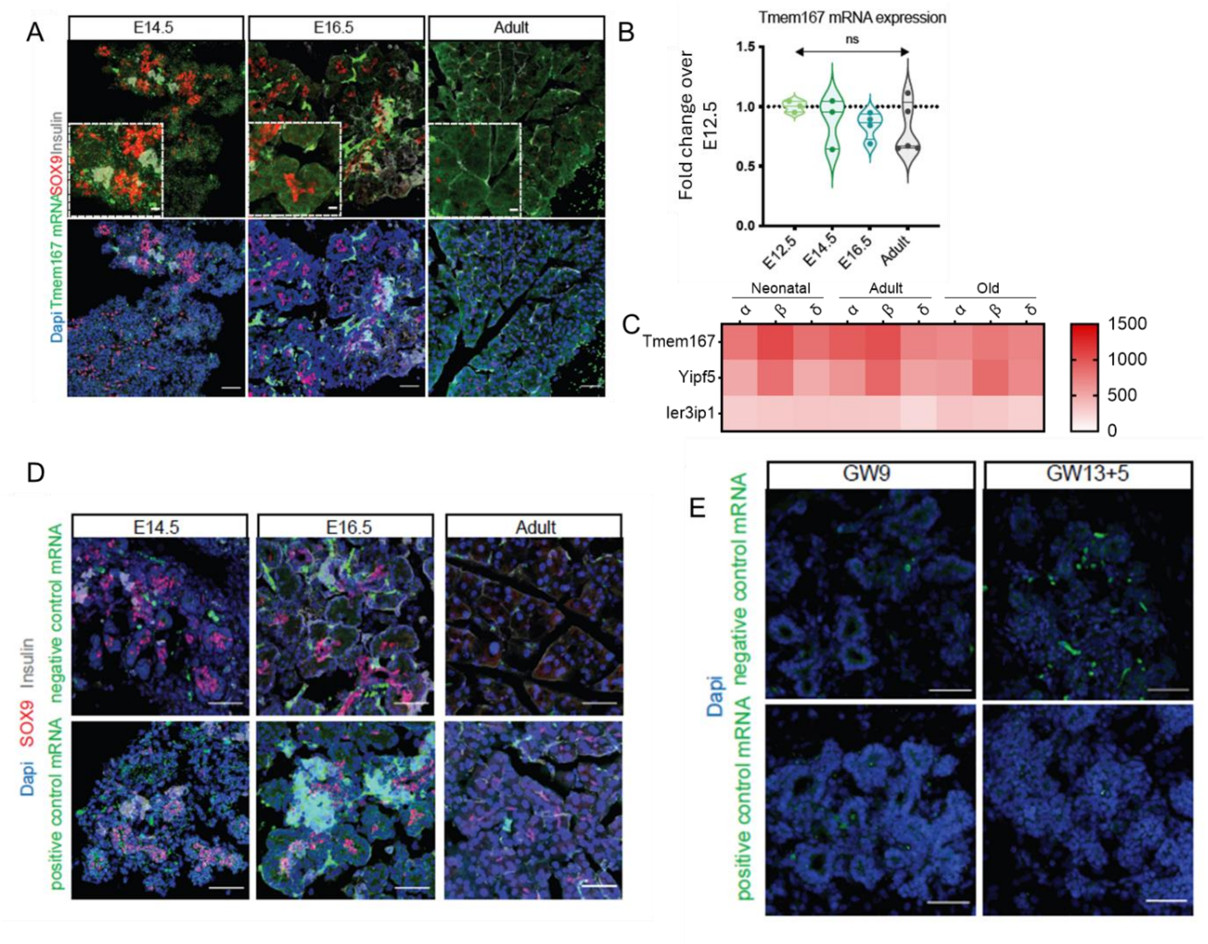

Figure S6: Expression pattern of Tmem167 in mouse pancreas

(A) RNAscope and immunolabeling of E14.5, E16.5 and adult mouse pancreas showing expression of *Tmem167* mRNA (green), insulin (grey) and Sox9 (red) and nuclear counterstaining (Dapi, blue). Scale bars 50 µm and 10 µm in the close up. (B) Quantification of *Tmem167* mRNA expression of E12.5, E14.5, E16.5 and adult mouse pancreas by qPCR. The expression level at E12.5 was set as 1.0 and other ages are expressed relative to this stage. Violin plots show median ± quartiles from 3-5 samples. (C) Heatmap generated from bulk RNA-seq data of FACS-sorted neonatal (1-week-old), adult (12-week-old) and old (18-month-old) mouse α, β, and δ cells showing expression of Tmem167, Yipf5 and Ier3ip1 (D) Control images of E14.5, E16.5 and adult mouse pancreas with positive and negative control mRNA (green), insulin (grey) and Sox9 (red) and nuclear counterstaining (Dapi, blue). Scale bars 50 µm. (E) Control images of GW9 and G13+5 human pancreas with positive and negative control mRNA (green, described in Supplementary Table 21) and nuclear counterstaining (Dapi, blue) Scale bars 50 µm.

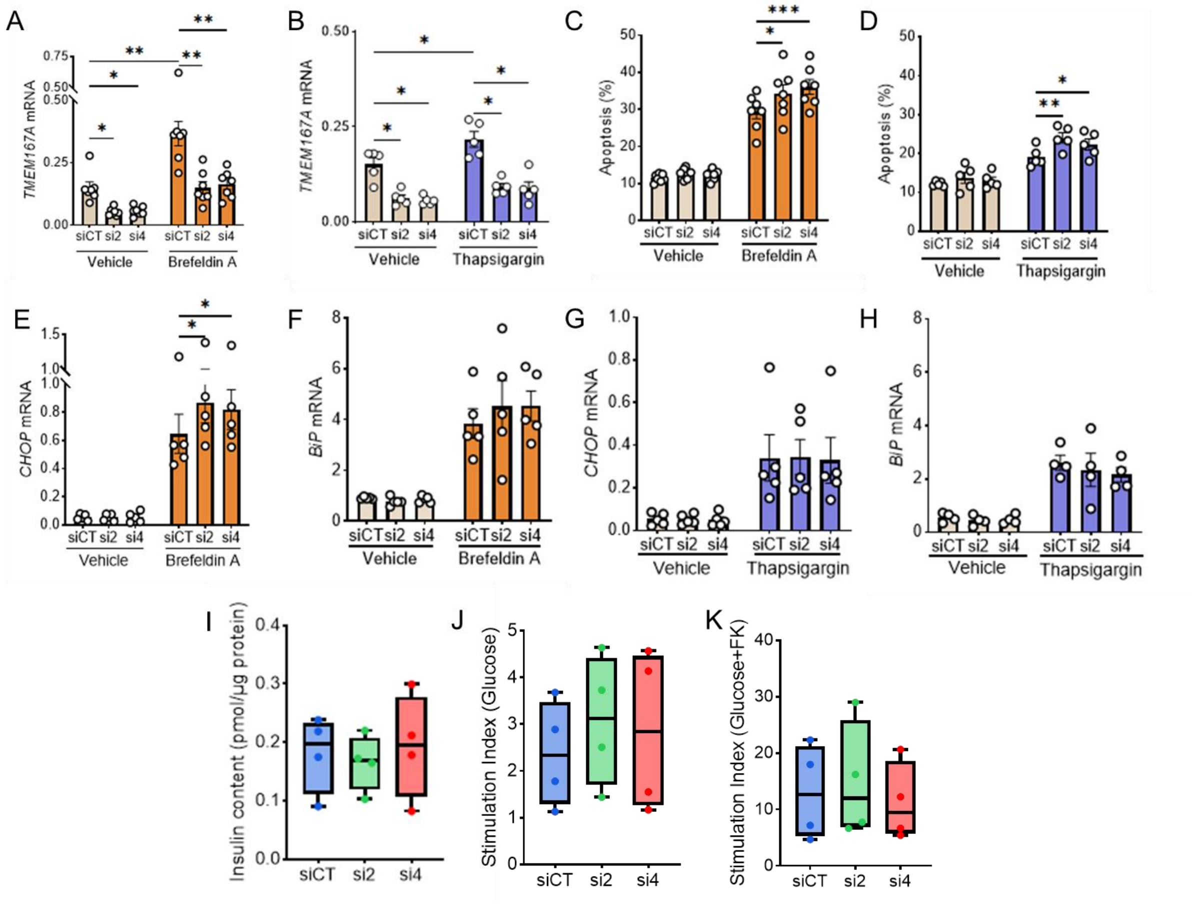

Figure S7: *TMEM167A* depletion sensitizes β cells to ER stress-induced apoptosis

EndoC-βH1 cells were transfected with 2 *TMEM167A* siRNAs (si2 or si4) or control siRNA (siCT). The cells were exposed for 16h to brefeldin A (0.025 mg/dL, orange bars) or for 24h to thapsigargin (1 µM, blue bars) (n=6-8). Gene expression was assessed by qPCR, normalized to the geometric mean of reference genes *ACTB* and *VAPA* (n=5-7). Apoptosis was assessed by Hoechst 33342/propidium iodide staining (C-D). (I) Insulin content normalized to total protein content after *TMEM167A* silencing (n=4). (J-K) Stimulation index of insulin secretion elicited by high glucose (20 mM) or high glucose (20 mM) plus forskolin (FK, 10 µM), calculated as stimulated insulin secretion divided by insulin secretion at 0 mM glucose (n=4). Insulin secretion data were normalized to total protein content. Individual data points represent independent experiments. Statistical significance was assessed in A and B by 2-way ANOVA with Tukey's correction, in C, D and E by 2-way ANOVA with Dunnett's correction. *p<0.05, **p<0.01, ***p<0.001. Error bars represent SEM from the mean. The median is shown by horizontal lines in the box plots; 25th and 75th percentiles are at the bottom and top of the boxes; whiskers represent minimum and maximum values.

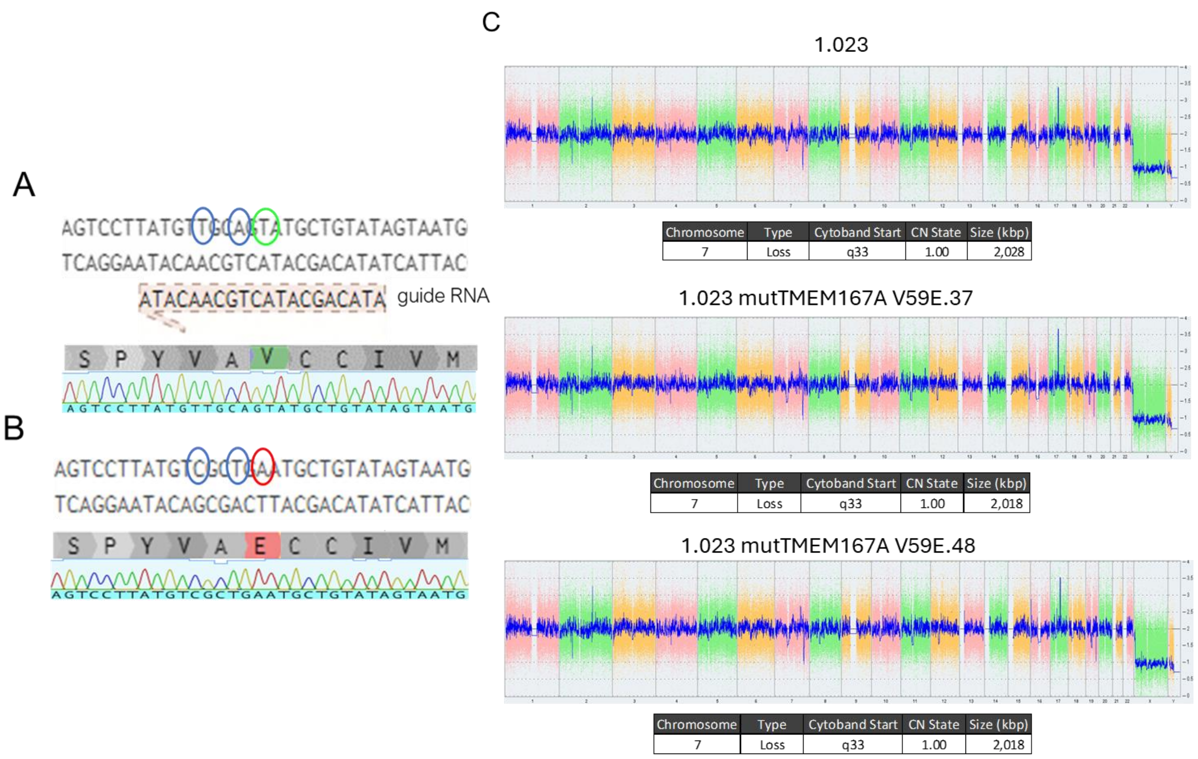

Figure S8: *TMEM167A* c.176T>A genome editing to generate V59E iPSC lines

(A) Wild type *TMEM167A* sequence in 1.023 iPSCs assessed by Sanger sequencing. gRNA sequence (brown frame) targeting exon 4 of *TMEM167A*; grey bar with letters shows amino acids within position 59 valine (green) encoded by GTA. (B) Successful genomic edit of c.176T>A in *TMEM167A* exon 4, where GAA generates the p.Val59Glu *TMEM167A* variant in position 59 (glutamate, red). Two silent edits (blue) were introduced to reduce the likelihood of gRNA binding after successful editing. (C) KaryoStat whole genome view of somatic and sex chromosomes of the 1.023 mother cell line, 1.023 mutTMEM167A V59E.37 and 1.023 mutTMEM167A V59E.48 cell lines. A value of 2 represents normal copy number state, 3 represents chromosomal gain and 1 chromosomal loss. Pink, green and yellow colours indicate the raw signal for individual chromosome probes; the blue signal represents normalized probe signal used to identify copy number and aberrations. The 1.023 iPSCs have a known loss in chromosome 7 that is also present in the V59E iPSCs. No differences between the mother and CRISPR/Cas9 edited cell lines were found.

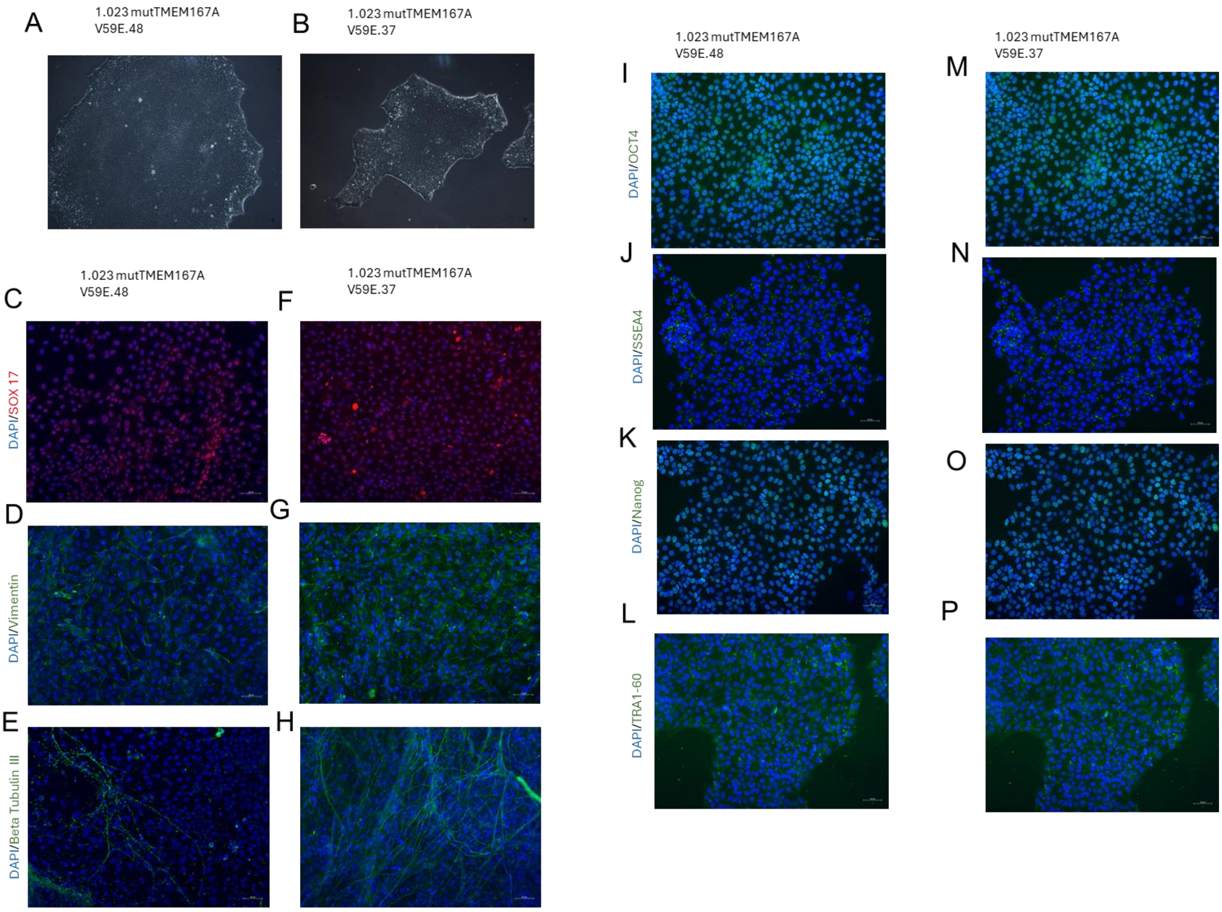

Figure S9: Validation of the pluripotency of mutTMEM167A V59E iPSCs

(A, B) iPSC morphology by bright field microscopy. (C-H) Immunostaining of iPSC-derived embryoid bodies for SOX17, vimentin and β-tubulin III, used as markers of endoderm, mesoderm and ectoderm, respectively. Scale bars 20 µM. (I-P) Immunostaining of iPSCs for pluripotency markers NANOG, OCT4, SSEA4 and TRA-1-60.

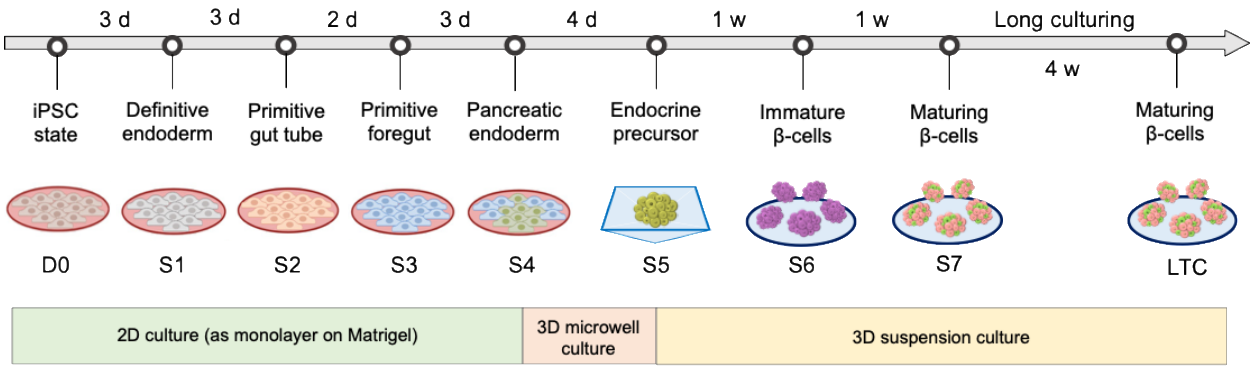

Figure S10: Overview of iPSC-islet differentiation protocol.

S1–S4 in 2D culture as monolayer on Matrigel coated plate, S5 in 3D microwells and S6–S7 and long-term culture (LTC) in 3D suspension culture.

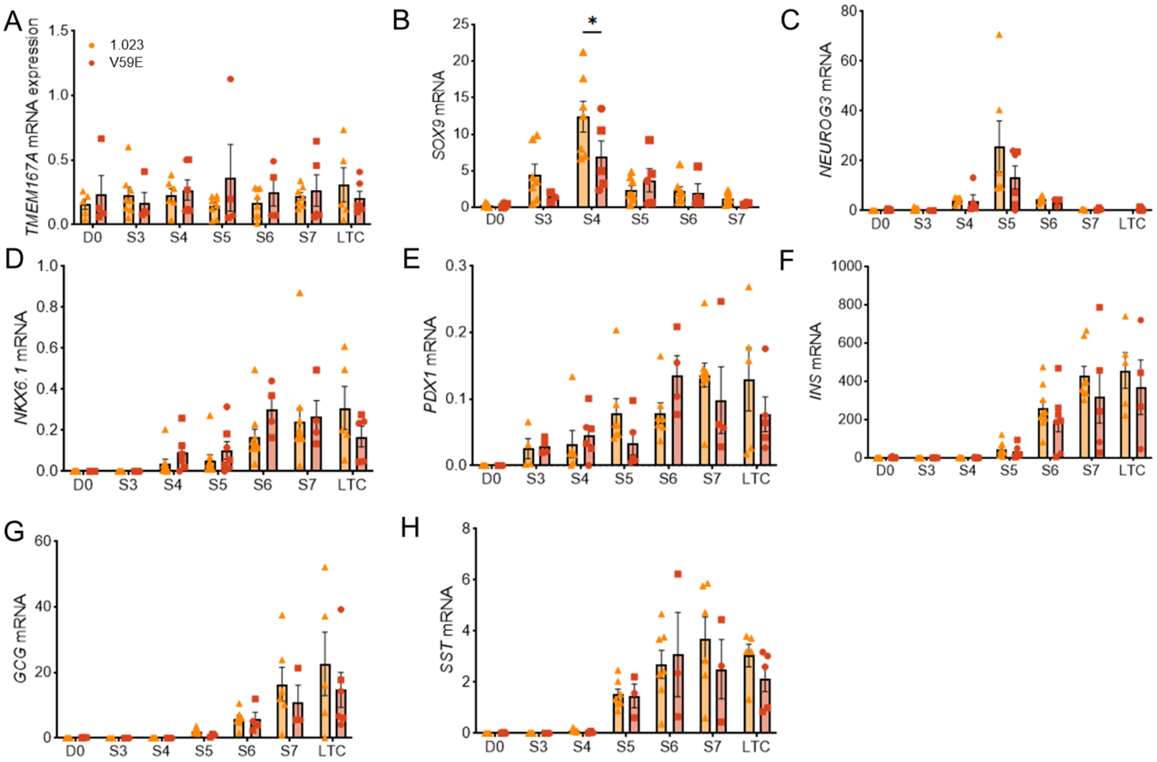

Figure S11: Gene expression along the iPSC differentiation into pancreatic β cells

mRNA expression in 1.023 (triangles), 1.023 mutTMEM167A V59E.37 (squares) and 1.023 mutTMEM167A V59E.48 (circles) iPSCs (D0) and along the differentiation from stage 3 to stage 7 and after long-term culture (LTC, n=3-12). mRNA levels, expressed as copies/µL, were assessed by qPCR and normalized to the geometric mean of reference genes ACTB and VAPA, expressed as copies/µL. Individual data points represent independent experiments. Statistical significance was assessed by 2-way ANOVA with Bonferroni's correction, *p<0.05. Error bars represent SEM.

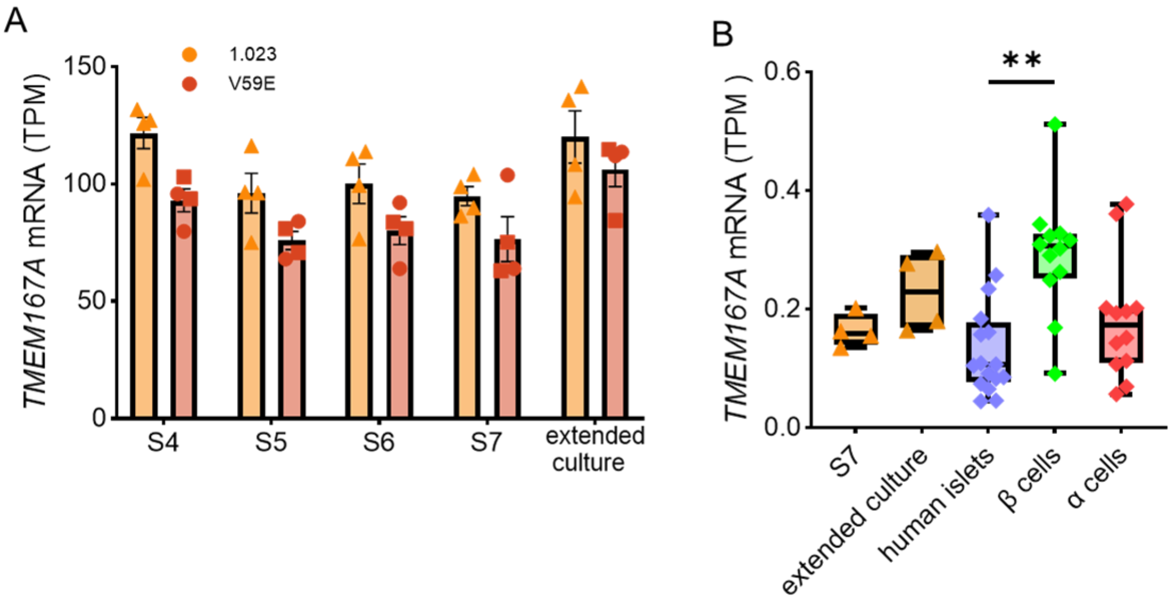

Figure S12: *TMEM167A* expression is stable along iPSC-β cell differentiation and similar in primary human islets

(A) *TMEM167A* mRNA expression in wild type and mutant cells along the β cell differentiation from S4 to extended culture stage based on bulk RNA-seq (n=4). (B) *TMEM167A* mRNA expression at the end of the differentiation (S7 and extended culture) in wild type iPSC-derived β cell aggregates, human islets from organ donors and FACS-sorted β and α cells from organ donors (n=4-16). In (B) TPM values from the different RNA sequencing studies have been normalized to expression levels of reference genes *ACTB* and *VAPA*. Triangles represent mother iPSC line 1.023, squares iPSC line 1.023 mutTMEM167A V59E.37, circles iPSC line 1.023 mutTMEM167A V59E.48 and diamonds human islets. Individual data points represent independent experiments. In (B) the median is shown by horizontal lines in the box plots; 25th and 75th percentiles are at the bottom and top of the boxes; whiskers represent minimum and maximum values. Error bars represent SEM. Statistical significance was assessed in (A) by Wald test corrected using false discovery rate by Benjamini-Hochberg method. The adjusted p value was >0.6. In (B) significance was assessed by Kruskal-Wallis test. **p<0.01.

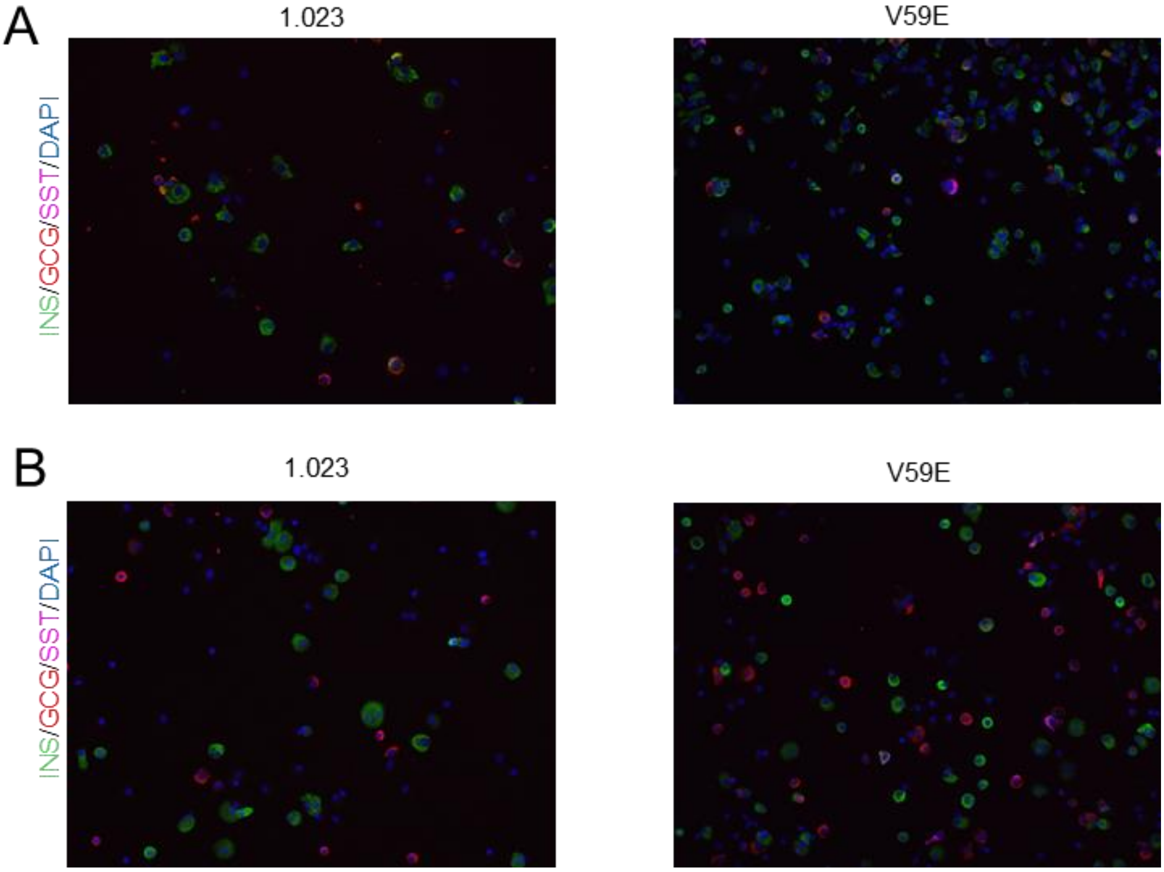

Figure S13: The *TMEM167A* V59E variant does not impact β cell differentiation

Representative immunocytochemistry images of dispersed S7 (A) and long-term culture (B) iPSC-β cell aggregates stained for insulin (green), glucagon (red) and somatostatin (pink). Nuclei were stained with DAPI.

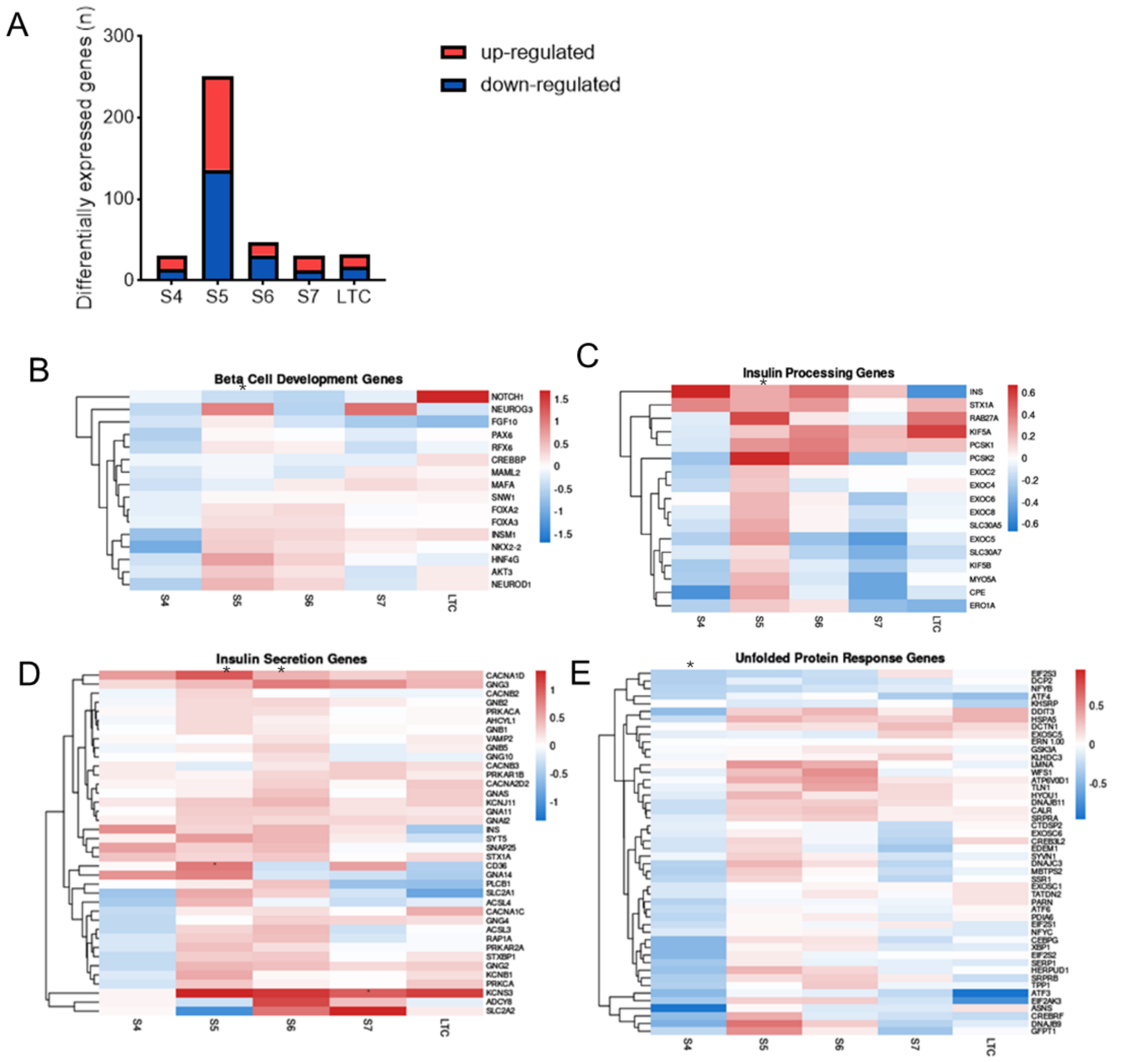

Figure S14: Enriched pathways along the differentiation into pancreatic β cells

(A) Quantification of differentially expressed genes comparing TMEM167A mutant with the 1.023 control iPSC lines across differentiation stages 4, 5, 6, 7 and long-term culture (LTC). (B-E) Heatmaps of the magnitude of change in gene expression comparing TMEM167A mutant vs the 1.023 iPSC control lines at S4, S5, S6, S7 and LTC. Colors represent log 2-fold change after differential analysis using DESeq2. Pathways displayed were significantly enriched or depleted in at least one stage of differentiation (marked by an asterisk). The genes shown for each pathway represent the union of leading-edge genes identified in stages with significant pathway enrichment. Significantly differentially expressed genes and deregulated pathways are marked with an asterisk. Pathway enrichment analysis was performed using fGSEA against the REACTOME database.

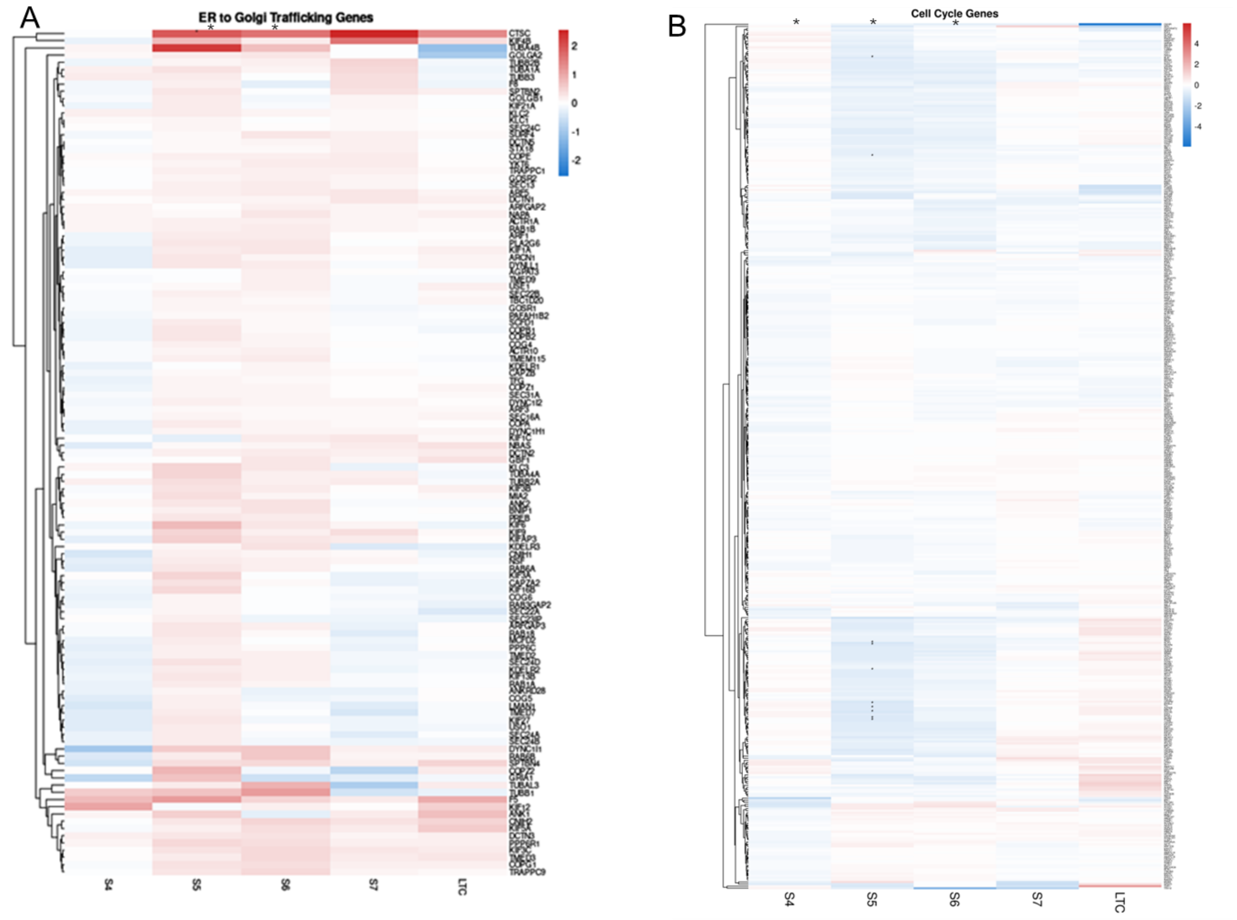

Figure S15: Enriched pathways along the differentiation into pancreatic β cells

Heatmaps of the magnitude of change in gene expression comparing the TMEM167A mutant vs the 1.023 iPSC lines at S4, S5, S6, S7 and long-term culture (LTC). Colours represent log 2-fold change after differential analysis using DESeq2. Pathways displayed were significantly enriched or depleted in at least one stage of differentiation (marked by an asterisk). The genes shown for each pathway represent the union of leading-edge genes identified in stages with significant pathway enrichment. Significantly differentially expressed genes and deregulated pathways are marked with an asterisk. Pathway enrichment analysis was performed using fGSEA against the REACTOME database.

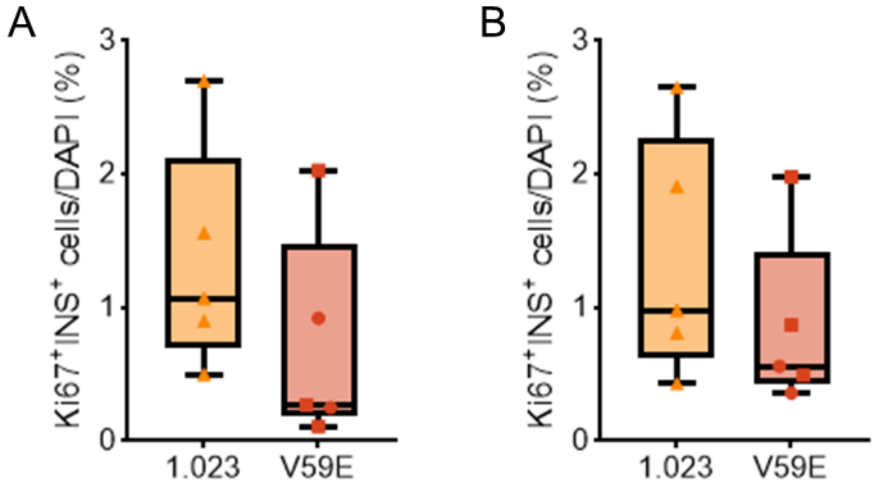

Figure S16: No alteration in the replication capacity of immature β cells

Quantification of dispersed stage 5 (A) and stage 6 (B) iPSC-β cells, double stained for insulin and Ki67 (n=5). Individual data points represent independent differentiations. Triangles represent mother iPSC line 1.023, squares iPSC line 1.023 mutTMEM167A V59E.37 and circles iPSC line 1.023 mutTMEM167A V59E.48. The median is shown by horizontal lines in the box plots; 25th and 75th percentiles are at the bottom and top of the boxes; whiskers represent minimum and maximum values.

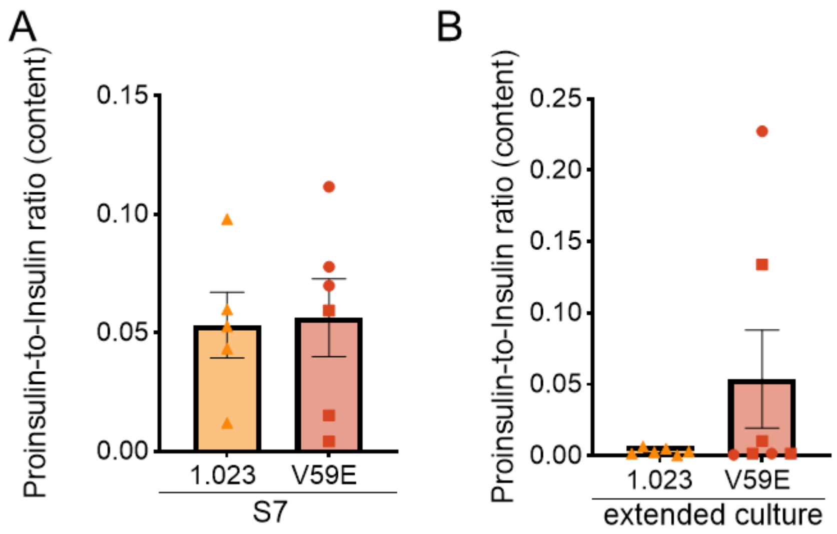

Figure S17: Conversion of proinsulin into insulin in TMEM167A V59E β cells

Proinsulin-to-insulin ratio in S7 (A) and extended culture (B) β cell aggregates differentiated from mother iPSC line 1.023 (triangles), 1.023 mutTMEM167A V59E.37 (squares) and cell line 1.023 mutTMEM167A V59E.48 (circles) (n=5-7). Individual data points represent independent experiments. Error bars represent SEM.

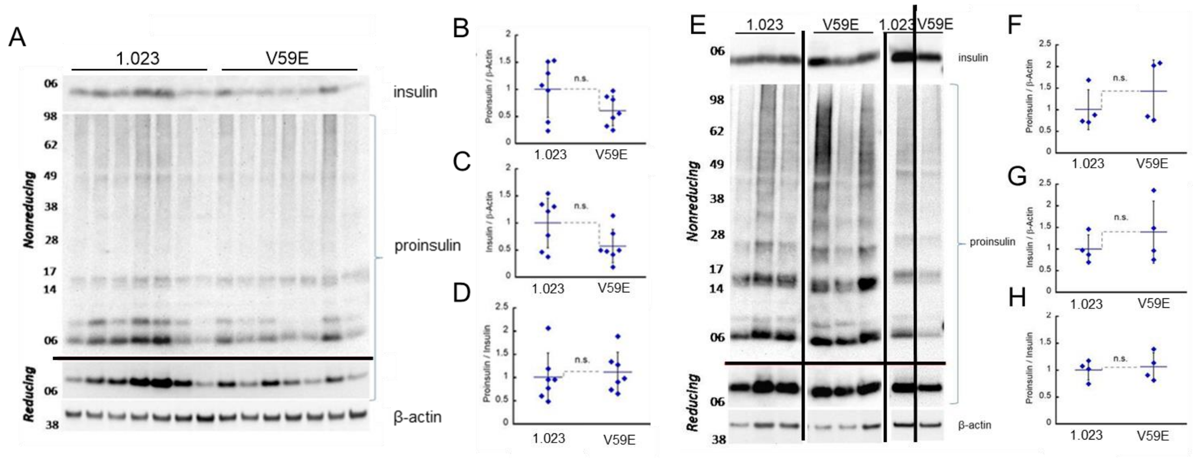

Figure S18: No alteration of proinsulin folding status in TMEM167A V59E β cells

Lysates of (A-D) stage 7 iPSC-β cells (n=7) and long-term differentiated iPSC-β cells (n=4) of mother iPSC line 1.023 and iPSC lines 1.023 mutTMEM167A V59E.37 and 1.023 mutTMEM167A V59E.48 were analysed under nonreducing or reducing conditions. (B-C, F-G) Quantification of proinsulin and insulin bands, normalized to loading control β-actin. (D, H) Proinsulin-to-insulin ratio. Individual data points represent independent differentiations. Statistical significance was assessed in B-D and F-H by two-tailed unpaired t-test. Error bars represent SD.

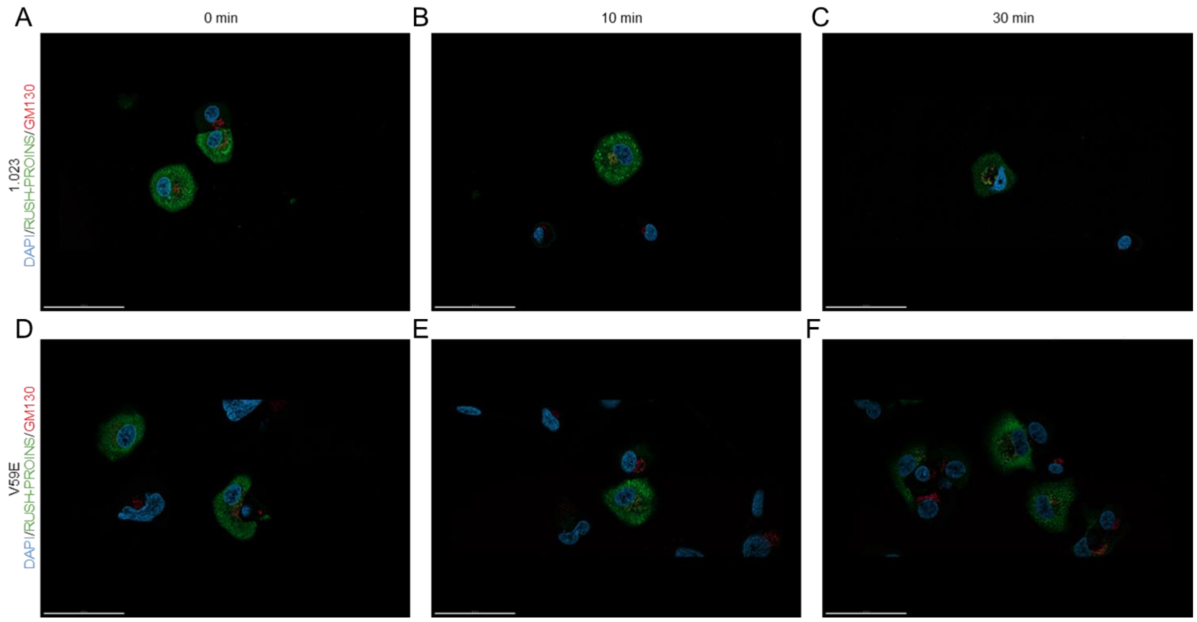

Figure S19: Impaired trafficking dynamics in TMEM167A V59E β cells

Representative images of dispersed stage 7 iPSC-β cell aggregates expressing GFP-proinsulin (green) and stained for Golgi marker GM130 (red) in a RUSH experiment at 0 and 10 and 30 minutes after biotin addition (A-C wild type cells, D-F mutant cells). Nuclei were stained with DAPI. Scale bars 50 μM.

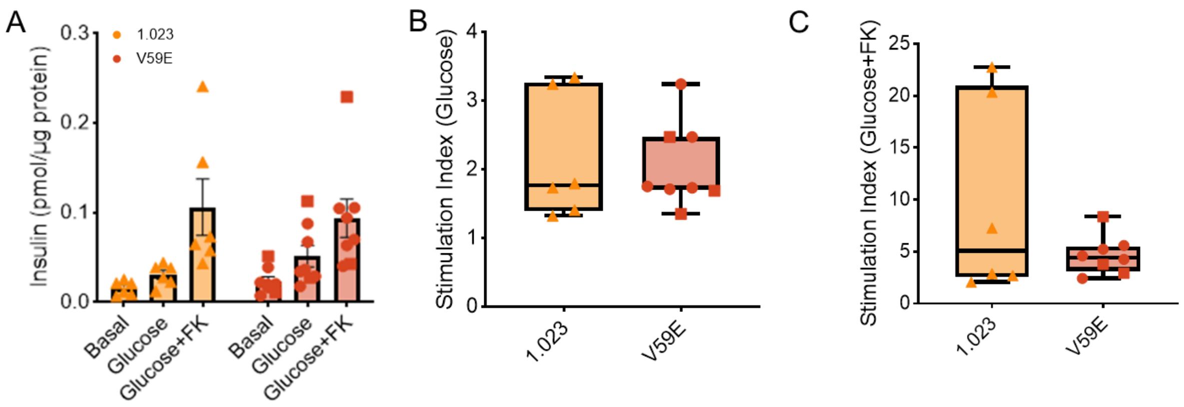

Figure S20: Intact insulin secretion in TMEM167A V59E β cells

(A) Insulin secretion elicited by low glucose (2.8 mM), high glucose (16.7 mM) or high glucose plus forskolin (FK, 10 µM) in stage 7 β cell aggregates differentiated from mother iPSC line 1.023 (triangles), 1.023 mutTMEM167A V59E.37 (squares) and cell line 1.023 mutTMEM167A V59E.48 (circles) (n=6-8). (B-C) Stimulation index at high glucose or high glucose plus forskolin, calculated as stimulated insulin secretion divided by insulin secretion at low glucose. Insulin secretion data were normalized to total protein. Individual data points represent independent experiments. Error bars represent SEM. The median is shown by horizontal lines in the box plots; 25th and 75th percentiles are at the bottom and top of the boxes; whiskers represent minimum and maximum values.

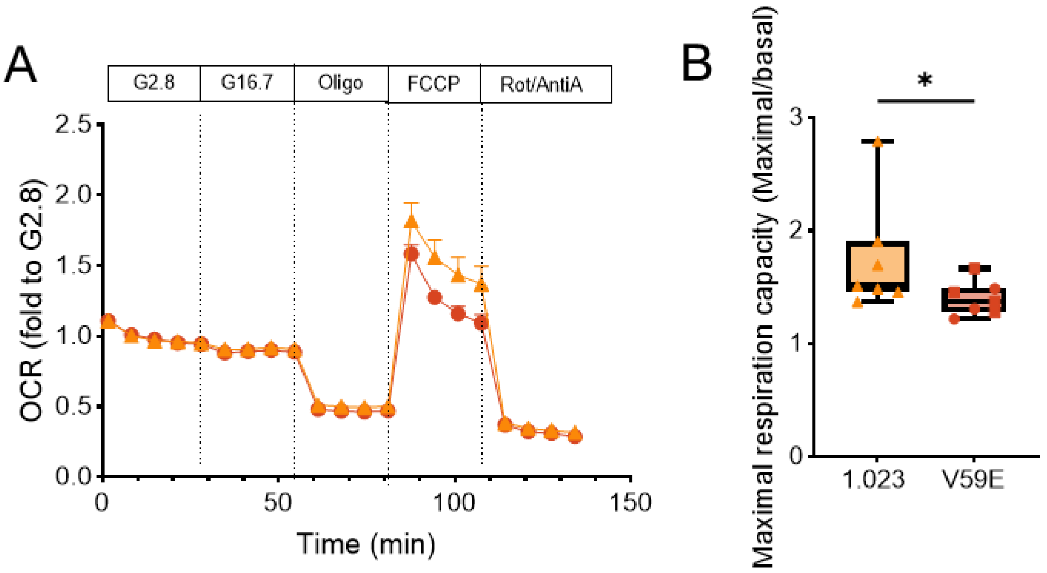

Figure S21: TMEM167A V59E β cells display reduced mitochondrial maximal respiration capacity

(A) Mitochondrial function by Seahorse measured in control and TMEM167A mutant β cells, normalized to G2.8. N=7 for control and TMEM167A mutant lines. Mitochondrial respiration was measured basally and after sequential injection of 16.7 mM glucose, ATP synthase inhibitor oligomycin (5 µM), oxidative phosphorylation uncoupler FCCP (4 µM), and electron transport chain inhibitors rotenone and antimycin (1 µM). (B) Maximal respiration capacity expressed as the ratio between maximal and basal respiration. Statistical significance was assessed in (B) by Mann-Whitney test. *$p<0.05$. The median is shown by horizontal lines in the box plots; 25th and 75th percentiles are at the bottom and top of the boxes; whiskers represent minimum and maximum values.

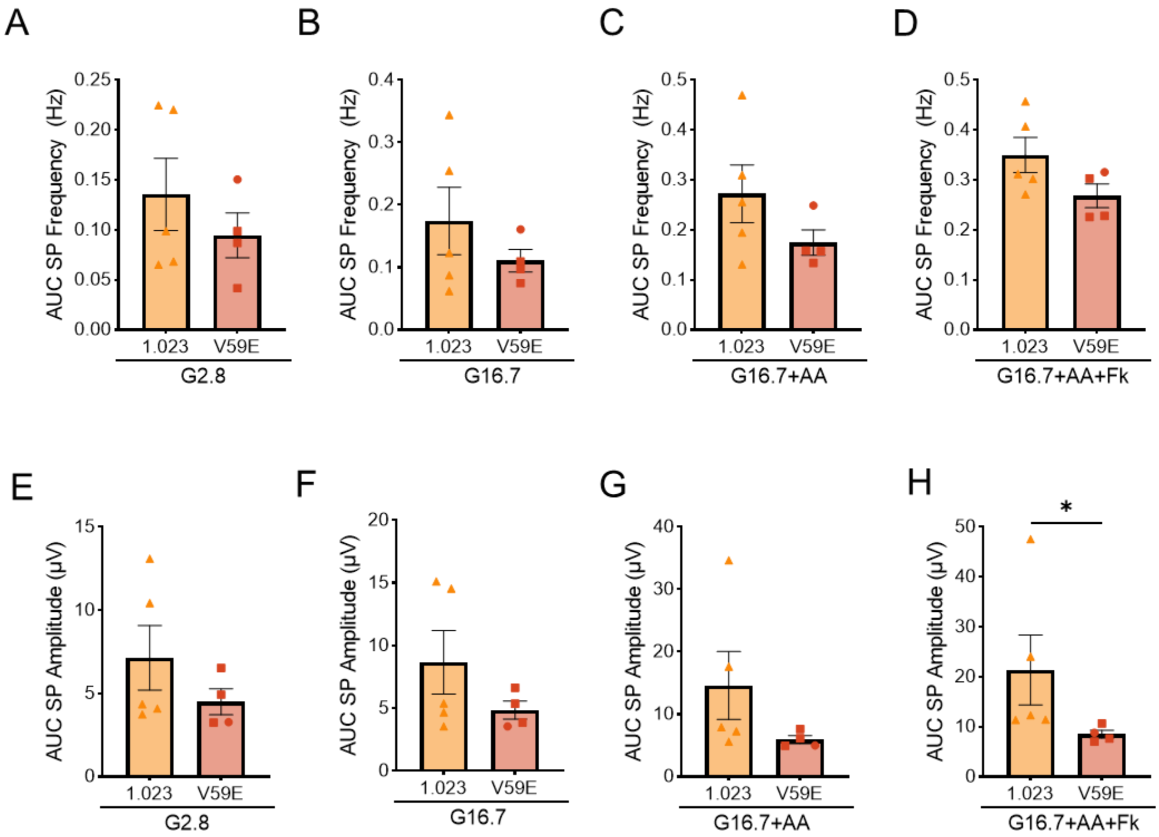

Figure S22: TMEM167A V59E β cells display reduced amplitude of slow potentials

Quantification of area under the curve of traces in Figure 4F, G for each phase. N=5 and n=4 independent differentiations for control and TMEM167A mutant cells, respectively. N=165 and n=121 electrodes for control and TMEM167A cells, respectively. Triangles represent mother cell line 1.023, squares 1.023 mutTMEM167A V59E.37 and circles 1.023 mutTMEM167A V59E.48. Statistical significance was assessed by Mann-Whitney test. *$p<0.05$. Error bars represent SEM.

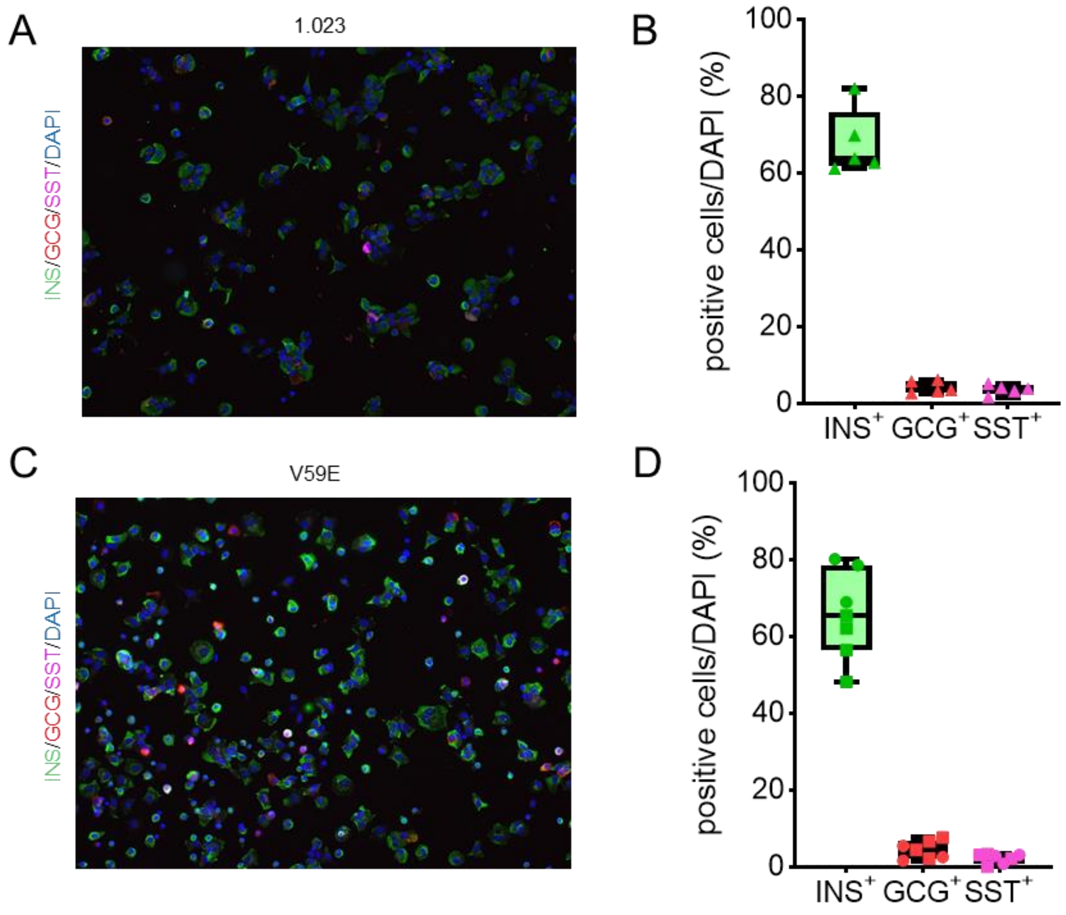

Figure S23: β cell enrichment by MACS

(A and C) Representative Immunofluorescence images and (B and D) quantification of dispersed stage 7 iPSC-β cell aggregates of mother iPSC line 1.023 (triangles), iPSC line 1.023 mutTMEM167A V59E.37 (squares) and iPSC line 1.023 mutTMEM167A V59E.48 (circles) that were enriched in β cells by MACS for the iPSC-β cell marker CD49a. Individual data points represent independent differentiations. The median is shown by horizontal lines in the box plots; 25th and 75th percentiles are at the bottom and top of the boxes; whiskers represent minimum and maximum values.

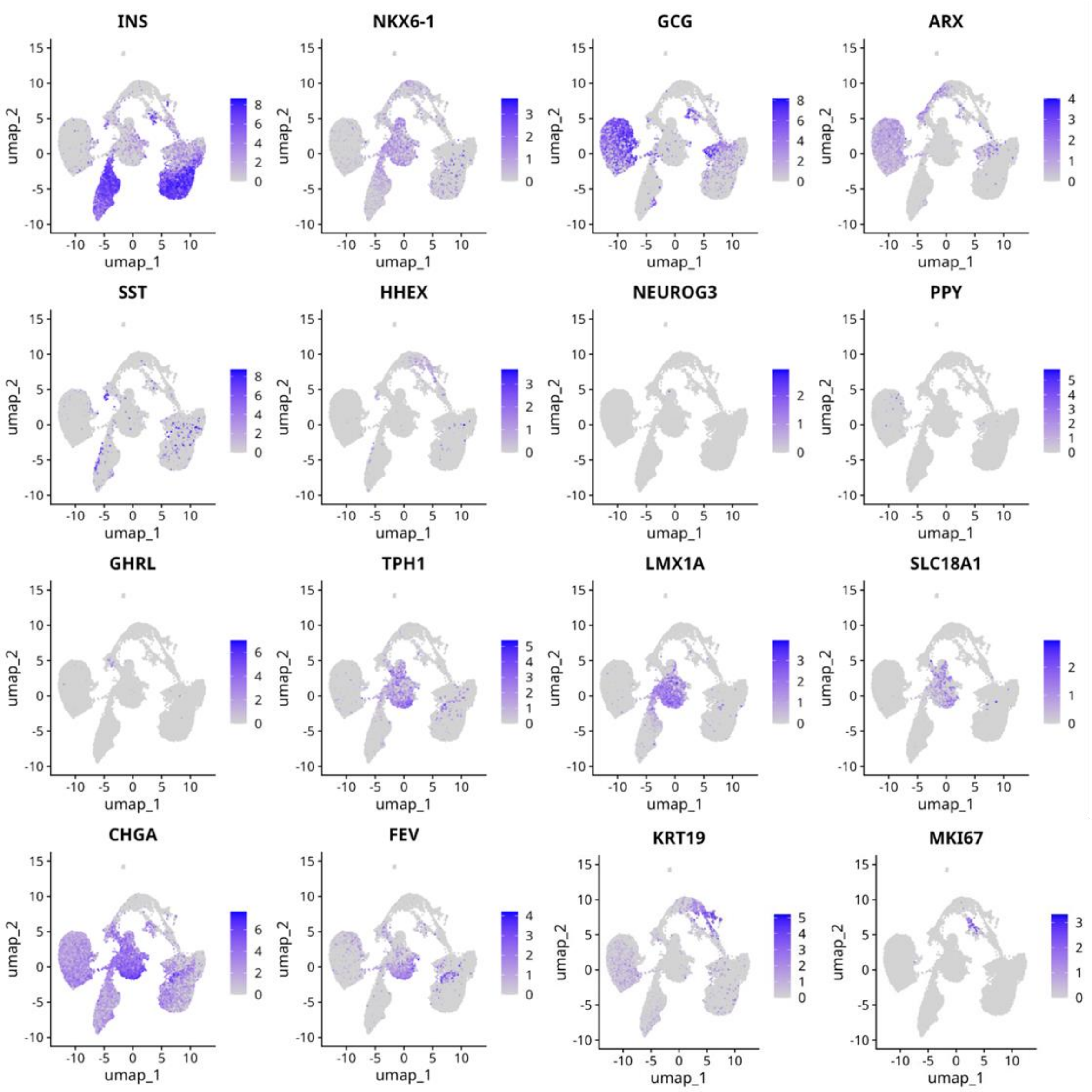

Figure S24: Gene markers for single cell clustering

Expression of the markers used to annotate the clusters in Figure 5A.

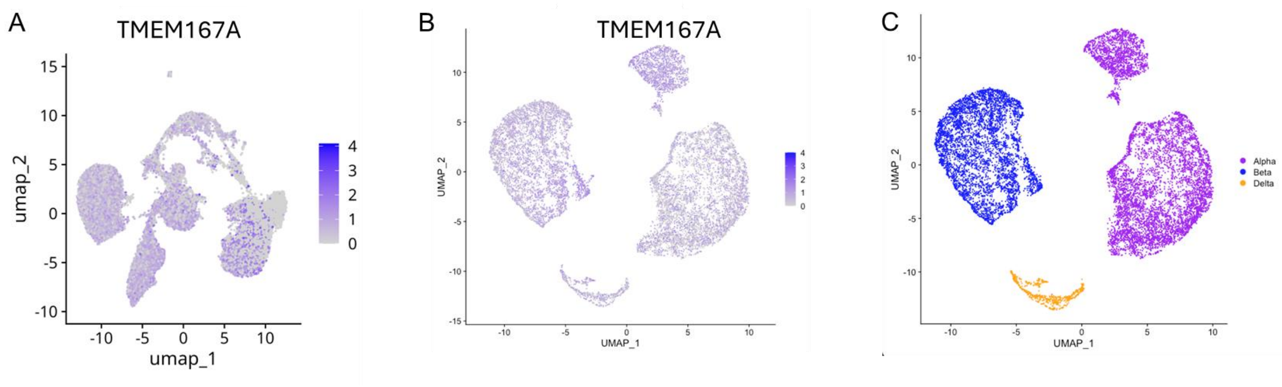

Figure S25: TMEM167A expression is ubiquitous in islet cells

(A) *TMEM167A* expression from single cell RNA-seq data of long-term cultured iPSC-aggregates of cell lines 1.023, mutTMEM167A V59E.37 and mutTMEM167A V59E.48. (B) *TMEM167A* expression in single cell RNA-seq of islet cells from organ donors from the HPAP collection, showing expression in α, β and δ cell clusters (C) α, β and δ cell clusters from organ donors from the HPAP collection.

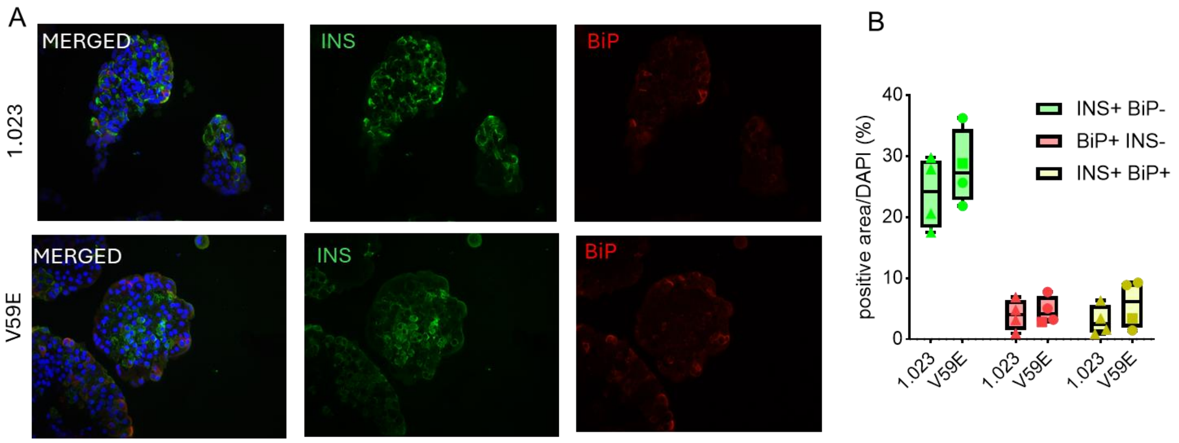

Figure S26: TMEM167A V59E β cells do not display overt ER stress

(A) Representative immunocytochemistry images and (B) quantification of stage 7 iPSC-β cell aggregates stained for insulin (green) and BiP (red); nuclei were stained with DAPI (n=4). Double positive cells are shown in yellow. Individual data points represent independent differentiations. Triangles represent mother iPSC line 1.023, squares iPSC line 1.023 mutTMEM167A V59E.37 and circles iPSC line 1.023 mutTMEM167A V59E.48. The median is shown by horizontal lines in the box plots; 25th and 75th percentiles are at the bottom and top of the boxes; whiskers represent minimum and maximum values.

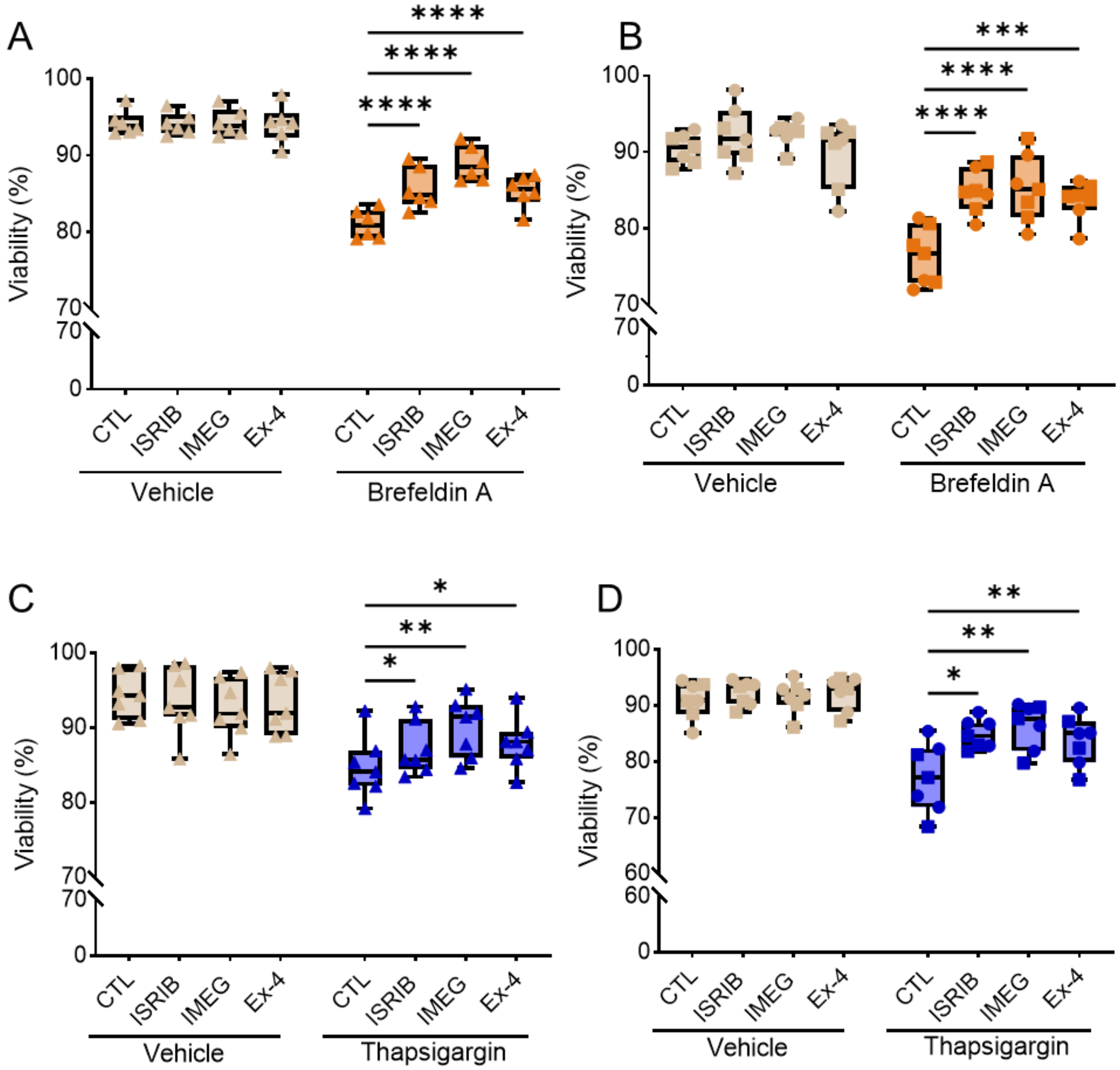

Figure S27: ISRIB, imeglimin and exendin-4 protect β cells from ER stress-induced apoptosis

Stage 7 iPSC-β cell aggregates were exposed or not for 24h to brefeldin A (0.025 mg/dL, A-B) or for 48h to thapsigargin (1 µM, C-D), alone or in combination with 200 nM ISRIB,1 mM imeglimin (IMEG) or 50 nM exendin-4 (Ex-4). Viability was assessed by Hoechst 33342/propidium iodide staining. Individual data points represent independent differentiations. Triangles represent mother iPSC line 1.023, squares iPSC line 1.023 mutTMEM167A V59E.37 and circles iPSC line 1.023 mutTMEM167A V59E.48. Statistical significance was assessed by 2-way ANOVA with Dunnett's correction. *$p<0.05$, **$p<0.01$, ***$p<0.001$, ****$p<0.0001$. The median is shown by horizontal lines in the box plots; 25th and 75th percentiles are at the bottom and top of the boxes; whiskers represent minimum and maximum values.

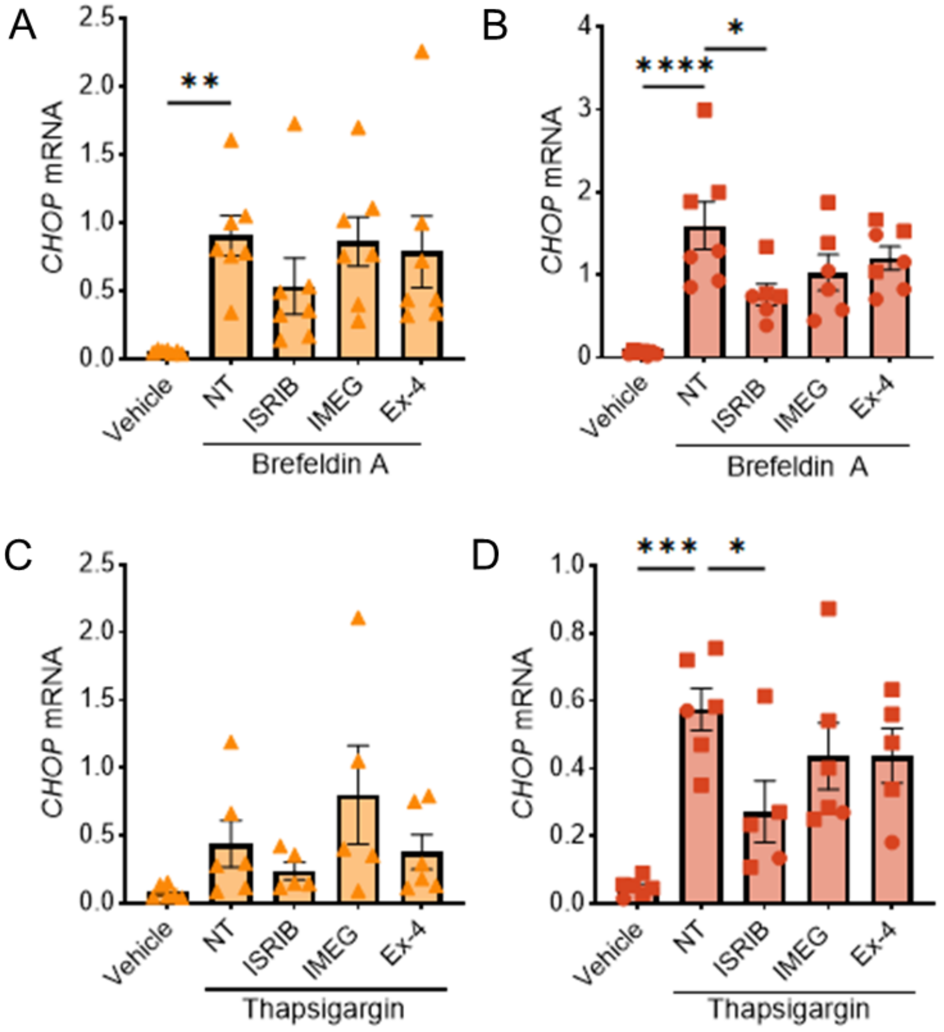

Figure S28: ISRIB reduces *CHOP* induction in TMEM167A V59E β cells

Stage 7 iPSC-β cell aggregates of mother iPSC line 1.023 (triangles), iPSC line 1.023 mutTMEM167A V59E.37 (squares) and iPSC line 1.023 mutTMEM167A V59E.48 (circles) were exposed or not for 24h to brefeldin A (0.025 mg/dL) or for 48h to thapsigargin (1 µM), alone (nontreated, NT) or in combination with 200 nM ISRIB, 1 mM imeglimin (IMEG) or 50 nM exendin-4 (Ex-4) (n=6-7). Expression of ER stress marker CHOP was assessed by qPCR and normalized to the geometric mean of reference genes ACTB and VAPA. Individual data points represent independent differentiations. Statistical significance was assessed by one-way ANOVA with Dunnett's correction. *p<0.05, **p<0.01, ***p<0.001, ****p<0.0001. Error bars represent SEM.
