# Supplementary figures and images for: Recessive TMEM167A variants cause neonatal diabetes, microcephaly, and epilepsy syndrome
Source: J Clin Invest. 2025 Sep 9;135(22):e195756. doi: 10.1172/JCI195756 (PMC12618065; doi:10.1172/JCI195756)

Unedited/uncropped gel used in supplementary figure 1

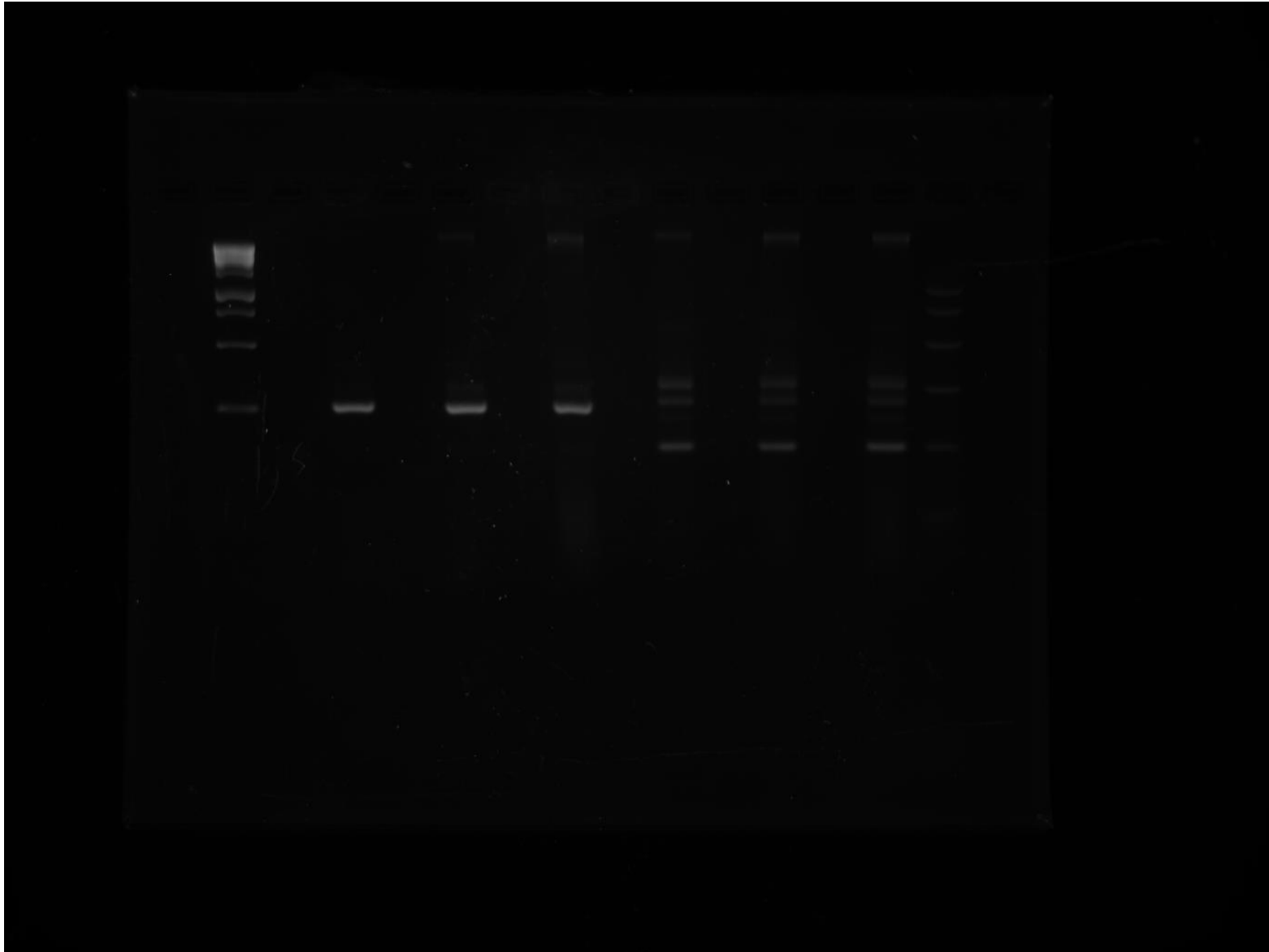

Supplement: Unedited blot and gel images [file jci-135-195756-s252.pdf]
